# Synergistic effect of natural chickpea leaf exudates acids in heterocyclization: a greener protocol for benzopyran synthesis

**DOI:** 10.1098/rsos.170333

**Published:** 2018-02-07

**Authors:** Snehali Mali, Sachin Shinde, Shashikant Damte, Suresh Patil

**Affiliations:** Synthetic Research Laboratory, PG Department of Chemistry, PDVP College, Tasgaon, Sangli district, 416312, Maharashtra, India

**Keywords:** benzopyran, *Cicer arietinum*, chickpea exudates, bio**-**catalyst, natural catalyst

## Abstract

Without using any toxic or hazardous reagent, ligand, acid, transition metal catalyst, additives/promoters and organic solvent, green Knoevenagel condensation and tandem Knoevenagel–Michael reactions have been successfully carried out by using *chickpea leaf exudates* as a naturally sourced Bronsted acid type bio-catalyst. The reaction proceeds in neat *chickpea leaf exudates* at room temperature in aqueous conditions in very short reaction times, and therefore, it is an evergreen and environmentally sound alternative to the existing protocols for benzopyran synthesis. In comparison to the conventional methods, this synthetic pathway complies with several key requirements of green chemistry principles such as the utilization of biodegradable catalyst obtained from renewable feedstock, auxiliary aqueous conditions, along with waste prevention. The same protocol was also extended to the synthesis of 2*H*-xanthene-1,8-diones by condensation of aromatic aldehydes with dimedone achieving excellent yields. Thus, the reported protocol offers an attractive option because of its ecological safety, environmental acceptance, sustainability, low-cost straightforward work-up procedure and with excellent values of green chemistry metrics as compared with other reported methods.

## 1. Introduction

While considering the increasing environmental pollution and its intensive impact on living systems, developing chemical processes using more environmentally acceptable chemicals, catalysts, solvents, atom-efficient methods and energy-efficient technologies eliminating waste production as well as employing renewable raw materials are experiencing a profound challenge to meet sustainability criteria [1,2]. In addition, the environmental risks posed by the toxic and volatile organic solvents have become a major concern. The reason is that the organic transformations employ higher consumption of solvents than the reagents and the employed solvents are difficult to recycle [3]; for the process to be in line with the green chemistry principles, the first task is to replace the toxic catalysts and hazardous organic solvents with green ones.

Performing organic reactions in water has attracted much attention over the past decades due to its numerous advantages such as being considerably safe, nontoxic, environmentally friendly and cheap [4–7]. Nowadays, biosynthetic processes involving bio-based solvents or catalysts such as Cyrene [8,9], glucose [10], dihydrolevoglucosenone (Cyrene) [11] and agro-waste based solvents or catalysts like metal based [12,13], Kolakhar [14], WEB [15–17], H_2_O_2_ in WERSA [18], water extract of papaya bark [19], have received much attention as a viable alternative for the development of green protocols for organic transformations [20–25].

In this regard, naturally sourced material as part of the chemical process offer excellent alternatives to harmful and toxic chemicals in being more environmentally friendly technologies due to their ease of abundance in availability, ability to act as catalysts, low or negligible toxicity, biodegradability, and non-hazardous properties as compared to chemical catalysts. Again, due to the high natural abundance, their production is potentially less expensive. While taking into account these views in the light of green chemistry, the use of naturally occurring materials play an important role in the development of environmentally benign chemical processes. Different natural materials are used as solid support as well as catalysts in a large number of reactions promoting the formation of final products. Natural materials such as different plant parts such as fruits, root extracts [26–32], clays [33,34], zeolites [35,36] and enzymes [37,38] are used effectively in numerous chemical transformations.

Therefore, the aim of the present work is to explore the synthetic utility of naturally sourced acid catalyst in organic transformations. The catalytic medium is sourced directly from the *chickpea leaf exudates* (CLE). From literature records it is well-known that the *Cicer arietinum* locally known as chana (or harbhara) in India; it is a plant species commonly cultivated on a farm. Chickpea (*Cicer arietinum* L.) is a grain legume adapted to dry and cool environments in southern and western Asia, northern and eastern Africa, Central and South America, and southern Europe. India produces more than 65% of the world's chickpeas (FAO, 1994), and the crop is an important source of protein for the local population.

**Figure 1.**
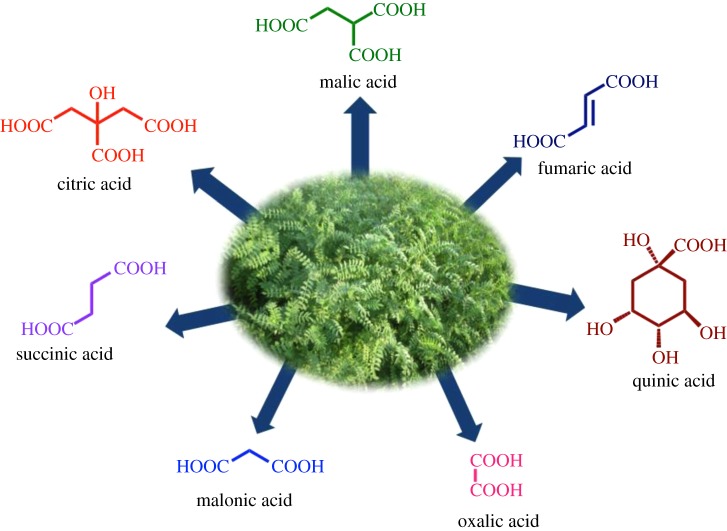
Structures of organic acids reported in CLE.

It has been reported that all external surfaces of the chickpea plants secrete organic acids [39,40], which consist almost exclusively of malic and succinic acids with small amount of quinic, citric and oxalic acids (figure 1) responsible for highly acidic character (1.1 pH) and has been correlated with reduced pod damage [41].

In view of these data and in continuation of our ongoing research in the development of green synthetic protocols [42–45], we thought that this amazing medium may serve as acidic catalyst, a better alternative to reported harmful corrosive acids for organic transformations.

Considering the significance of naturally sourced catalysts, in this work, we explore the catalytic activity of CLE as natural green acidic medium in the synthesis of benzopyranes starting from readily available dimedone and salicylaldehydes in presence of aqueous medium and at ambient temperature. To the best of our knowledge, the pronounced catalytic effect of CLE as natural acid catalyst is being described for the first time for organic synthesis.

Benzopyran derivatives act as anti-*Helicobacter pylori* agents [46], reductase inhibitors [47], selective thrombin (THR) inhibitors [48] PPAR *α*/*γ* agonists [49], potassium channel opener [50]. Benzopyrans also exhibit anti-inflammatory [51], insulin-sensitizing [52], antibacterial [53] and antimicrobial activities [54]. Recently, some benzopyran derivatives have been synthesized by the reaction of substituted salicylaldehydes with dimedone using different catalysts such as 2,4,6-trichloro-1,3,5-triazine [55], p-TSA [56], KF/Al_2_O_3_ [57], triethylbenzylammonium chloride [58], ionic liquid [59,60] and cellulose sulphonic acid [61].

Although a majority of these techniques were found to be effective in carrying out this cyclization, most of these protocols are associated with several drawbacks such as the use of expensive ligands, use of toxic or hazardous reagents, the requirement of a large amount of catalysts, use of transition metal complex catalysts often requiring activation by elevated temperatures, use of organometallic compounds, expensive and harsh reaction conditions, longer reaction times, use of organic solvents, and the generation of toxic by-products. Therefore, there is a continued interest in developing an efficient and safer protocol employing mild and green reaction conditions. The designer of a chemical process must choose from alternative raw materials, auxiliary materials such as solvents and catalysts, reaction pathways and reaction conditions and these design choices can have a very significant impact on the overall environmental performance of a chemical process [62–65].

## 2. Experimental

### 2.1. Materials and methods

All commercial reagents and solvents were obtained from Sigma-Aldrich and used without purification. Melting points (mp) were determined with DBK-programmable melting point apparatus and are uncorrected. Infrared (IR) spectra were recorded in potassium bromide pellets on a Bruker ALPHA FT-IR spectrometer. The ^1^H NMR spectra (300 MHz) and ^13^C NMR spectra (75.5 MHz) were measured with Avance-300 instrument and chemical shifts (*δ*) are reported in parts per million in CDCl_3_ as a solvent with tetramethylsilane (TMS) as an internal standard. Desired products were obtained only by stirring reaction mixture on magnetic stirrer (Remi, Model No. 10 ML DX Stirrer). The purity of products and completion of reaction was checked by thin layer chromatography (TLC) on Merck silica gel (60 F_254_) plates. Spots on the TLC plates were visualized using ultraviolet (UV) light. Microanalyses of C, H and N were obtained on a Carl Erba EA 1108. All spectral data (FT-IR, ^1^H and ^13^C) are given in the electronic supplementary material. The pH of *Cicer arietinum* exudates and aqueous solutions of organic acids were measured using Pro-Lab 3000 laboratory pH-meter.

### 2.2. General procedure for collection of chickpea leaf exudates

For quantitative collection of catalyst, *chickpea* crops cultivated on various lands were selected and then exudates were collected manually using clean cotton napkin by absorption-wringing process. The turbid exudate collected was then purified by filtration through activated charcoal and obtained exudate was clear and denominated as CLE-catalyst. The collected catalyst was stored several days at 5°C and used as a catalyst for benzopyran synthesis.

### 2.3. Typical procedure for synthesis of 9-(2-hydroxy-4,4-dimethyl-6-oxo-cyclohex-1-enyl)-3,3-dimethyl-2,3,4,9-tetrahydroxanthen-1-one (3a)

A 25 ml round bottom flask was charged with salicylaldehyde (1.0 mmol), 5,5-dimethyl 1,3-cyclohexanedione (2.0 mmol), CLE-catalyst (0.3 ml) and water (5 ml), and reaction mixture was stirred at room temperature on magnetic stirrer till the completion of reaction as indicated by TLC (ethylacetate : hexane 4 : 6). The solid products was separated by simple filtration through a Buckner funnel, washed with cold water and recrystallized from 96% ethanol (5 ml). The identity of the compound was ascertained on the basis of FT-IR, ^1^H NMR and ^13^C NMR spectroscopy (figure 3*a*–*c*).

For the preparation of compounds 3a-s, 5a-c and 6a-k the same procedure was followed, by changing respective reactants, and synthesized compounds were characterized by physical and spectral analysis. The physical and spectroscopic data are consistent with the proposed structure and in harmony with the literature values.

## 3. Results and discussion

**Scheme 1.**
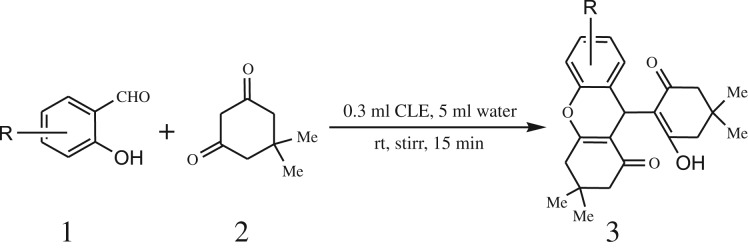
General reaction of salicylaldehyde with 1, 3-dimedone.

A simple, green, efficient and convenient method for the synthesis of benzopyran derivatives 3a-s from different 2-hydroxy aldehydes and 1,3-dimedones is presented (scheme 1).

**Figure 2.**
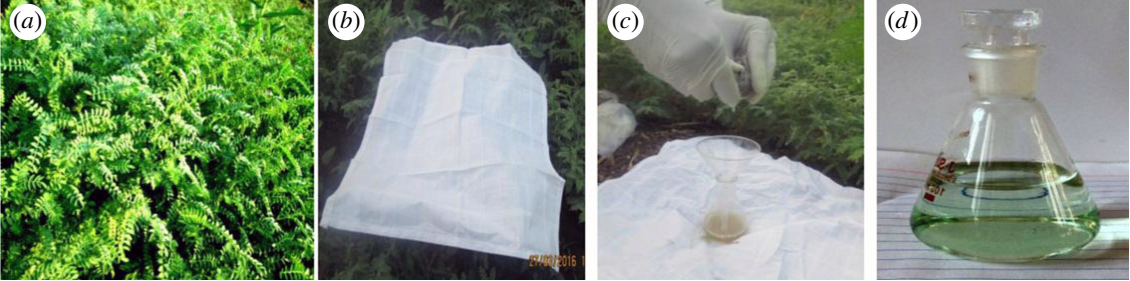
(*a*) Chickpea leaves, (*b*) absorption of exudates by cotton napkin, (*c*) wringing of exudates from cotton napkin, (*d*) collected clear CLE-catalyst after filtration.

At the beginning, we focused our attention on quantitative collection of exudates and for this, growing period of chickpea plant from time of cultivation was considered. For this purpose different varieties of cultivated crops on various lands were selected and then exudates were collected manually using clean and soft cotton napkin by absorption-wringing process. The good quantity of exudates was obtained when it was collected from leaves of plants which were in the stage just before flowering (7–8 weeks from cultivation) at early morning (05.00 to 06.00). The turbid exudates obtained were then filtered through activated charcoal to remove impurities to get clear exudates (figure 2). The pH of exudates was measured using pH-meter and it was found to be 1.1, and therefore, it was used as Bronsted acid type bio-catalyst for this protocol.

The urgent need for the development of green and sustainable processes for the use of natural ‘feedstocks’ in chemical synthesis as an alternative to hazardous organic solvents or other metal-based catalysts is widely recognized [66–71] and the minimization of energy requirements in chemical processes presents an important principle in green chemistry [72].

Inspired by this fact and continuing our research with the aim of lowering the environmental impact of organic reactions, we turned our attention to *chickpea leaf exudates*, as easily available natural feedstock. To the best of our knowledge this is the first report of a simple protocol for the benzopyran synthesis at room temperature without using any transition metal catalyst, ligand, base, toxic or hazardous reagent, additives/promoters and organic solvent. In this communication, we report on the use of *chickpea leaf exudates* as an all-in-one mixture of solvent, catalyst, promoters etc. for heterocyclization.

To optimize the reaction conditions, 25 ml round bottom flask was charged with salicylaldehyde **1** (1.0 mmol), 1,3-dimedone **2** (2.0 mmol), *chickpea leaf exudates* (0.3 ml) and the reaction mixture was stirred at room temperature as a model reaction. After 1 h low yield (30%) of corresponding product was observed on TLC (table 1, entry 3). On increasing or decreasing catalytic amount (0.1–1 ml), no significant improvement in the result was obtained after prolonged reaction time (table 1, entries 1–6). We continued our efforts for improvement in the result; we decided to evaluate model reaction in different organic solvents as well as in aqueous medium. To our outmost expectations, the reaction to perform in aqueous media, the reaction proceeded very well, and 96% yield was obtained when *chickpea leaf exudates* (0.3 ml) were employed with water (5 ml) under identical conditions (table 1, entry 7). In order to check and verify further the effect of the organic solvent on the yield of the product, the model reaction was performed in methanol, ethanol, DCM, acetonitrile, toluene (table 1, entry 8) which afforded product in moderate yields. Furthermore, we also estimated the catalyst–solvent proportion for better conversion of reactants into products by changing catalyst–solvent ratio. The result showed that, 0.3 : 5 catalyst : solvent ratio was suitable (0.15 N, 0.045 mmol acid content determined by titration of catalyst with standard alkali) medium for smooth conversion of reactant to the product with respect to time and yield. From these results, it was also revealed that further decreasing or increasing the catalyst : solvent proportion reduces the yield of desired product dramatically (table 1, entry 9). Moreover, the catalyst-free condition was also examined; the result observed was viscous reaction system and no yield, which indicates that the role of catalyst is decisive for benzopyran 3 formation (table 1, entry 10).

**Table 1.** Optimization of reaction conditions for model reaction.^a^ 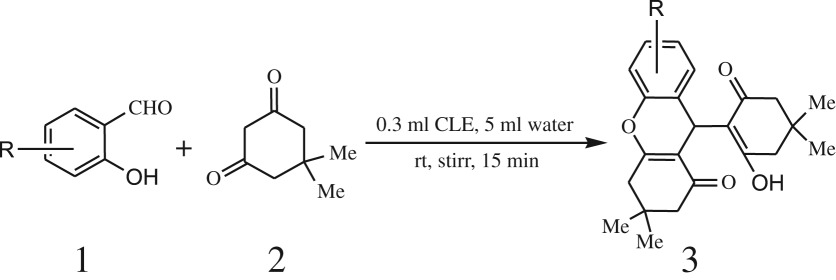

entry	catalyst amount (ml)	solvent 5 ml	temp (°C)	time (min)	yield^b^ (%)
1	0.1	—	RT	60	10
2	0.2	—	RT	60	23
3	0.3	—	RT	60	30
4	0.4	—	RT	60	30
5	0.5	—	RT	60	30
6	1.0	—	RT	60	31
7	0.3	water	RT	15	96
8	0.3	ethanol	RT	15	94 (88, 34, 43, 29)^c^
9	0.3	water	RT	15	(65, 93, 90, 34)^d^
10	—	water	Rt	15	no reaction

^a^Reaction conditions: salicylaldehyde **1** (1.0 mmol) and 1,3-dimedone **2** (2.0 mmol), catalyst, water (5 ml), room temperature.

^b^Isolated yield based on salicylaldehyde.

^c^Yields with organic solvents: methanol, DCM, acetonitrile, toluene.

^d^Isolated yield when 1, 3, 7 and 10 ml water was used.

**Figure 3.**
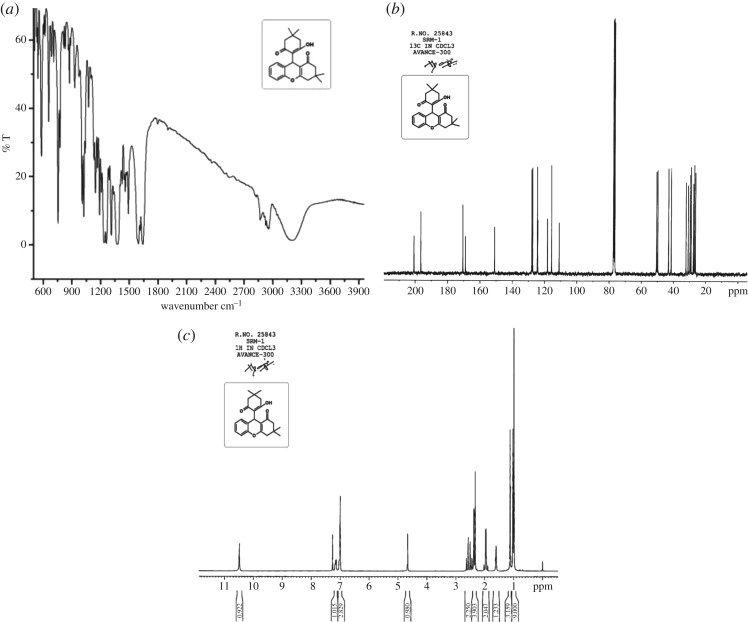
(*a*) FT-IR spectrum of compound 3a. (*b*) ^1^H NMR (CDCl_3_) spectrum of compound 3a. (*c*) ^13^C NMR (CDCl_3_) spectrum of compound 3a.

On the completion of the reaction as monitored by TLC, the product was separated out by simple filtration, successively washed with cold water, and recrystallized from ethanol (96%) which afforded the corresponding product of high purity. Pure products obtained by recrystallization from ethanol were characterized by their physical constants and spectral techniques. In ^1^H NMR spectrum (figure 3*b*) of the product of the model reaction (table 3, entry 1), observation of sharp singlet at *δ* 10.50 due to enolic proton, and at *δ* 4.65 corresponding to tertiary C-H proton as well as incorporation of 23 signals in ^13^C NMR spectrum (figure 3*c*) confirms its formation. Further, in FT-IR spectrum (figure 3*a*), observation of broad band due to enolic -OH at 3207 cm^−1^ and at 1628 cm^−1^ (two should be there) corresponding to *α*, β-unsaturated cyclic carbonyl group supports its formation.

As mentioned earlier, the acidic nature of *chickpea leaf exudates* due to presence of several organic acids. For comparison purpose, we carried out the model reaction under same reaction conditions using aqueous solution (5%) of various organic acids (table 2, entries 1–4). From results, it reveals that catalyst obtained from *chickpea plant* was found to be excellent with respect to time as well as yield of the product (table 2, entry 5) suggesting that the synergetic effect of several organic acids in *chickpea leaf exudates* showed superior catalytic activity for smooth and rapid conversion reactant into desired product with excellent yield in shorter reaction time.

**Table 2.** Comparison of efficiency of different organic acids with CLE catalyst for benzopyran synthesis.^a^ 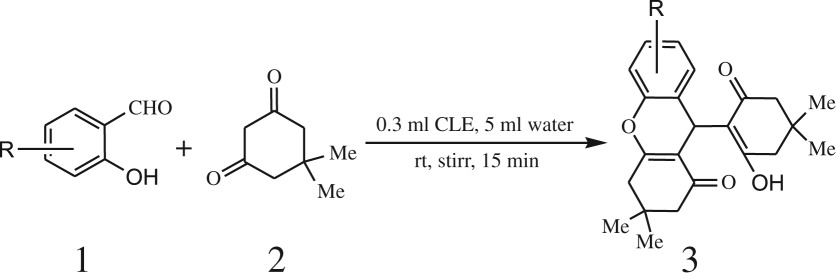

entry	catalyst (ml)	pH^b^	time (min)	yield^c^ (%)
1	succinic acid	2.86	60	n.a.
2	citric acid	2.35	60	n.a.
3	malic acid	2.12	60	41
4	oxalic acid	1.95	60	56
5	CLE	1.10	15	96

^a^Reaction conditions: salicylaldehyde **1** (1.0 mmol) and 1,3-dimedone **2** (2.0 mmol), catalyst (0.3 ml), water (5 ml) room temperature.

^b^pH of 5% aqueous solution of carboxylic acids.

^c^Isolated yield based on salicylaldehyde.

**Scheme 2.**
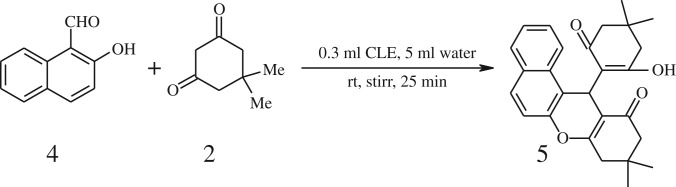
Reaction of 2-hydroxy naphthaldehyde with 1, 3-dimedone.

After optimization of reaction condition, the condensation reactions were carried out in *chickpea leaf exudates* (0.3 ml) at ambient temperature using a series of structurally diverse salicylaldehydes with 1,3-ketones (scheme 2, table 3). On the completion of reactions as monitored by TLC, the reaction mixtures were filtered to isolate products and purified by recrystallization from ethanol (96%). The reactions of salicylaldehydes, bearing electron-donating (-OCH_3_) as well as electron-withdrawing groups (-NO_2_, -Cl, -Br) underwent successfully.

**Table 3.** CLE-catalysed synthesis of benzopyrans.^a^ 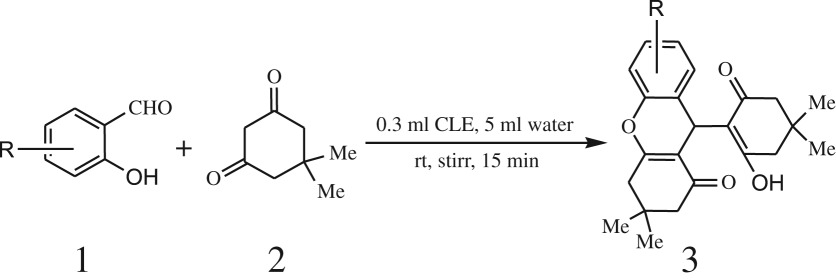

entry	aldehydes	1,3-diketones	products	time (min)	yield^b^ (%)	M.P°C (observed)	M.P°C (reported)
1	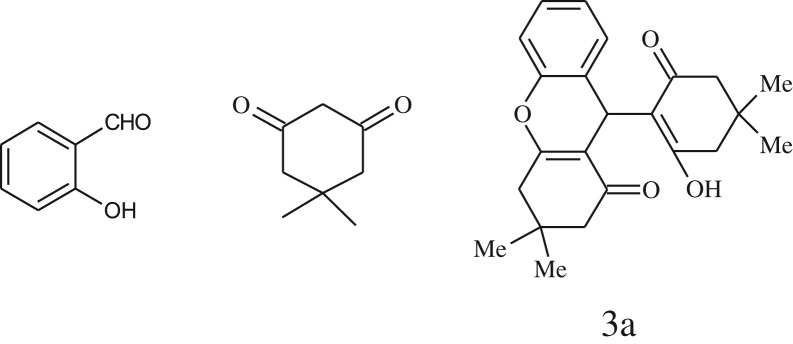			15	96	210–212	211–214 [73]
2	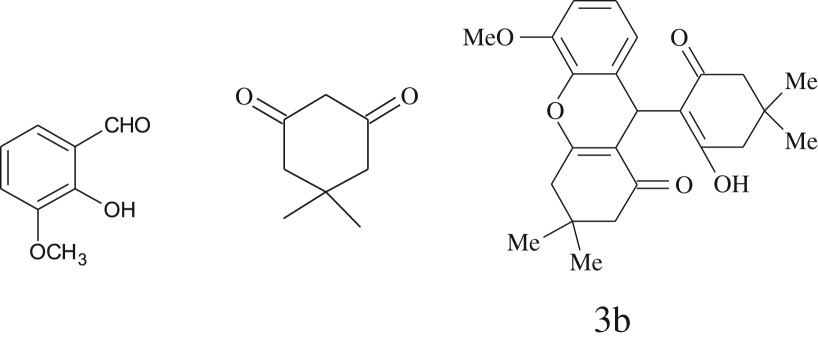			25	89	228–230	229–231 [73]
3	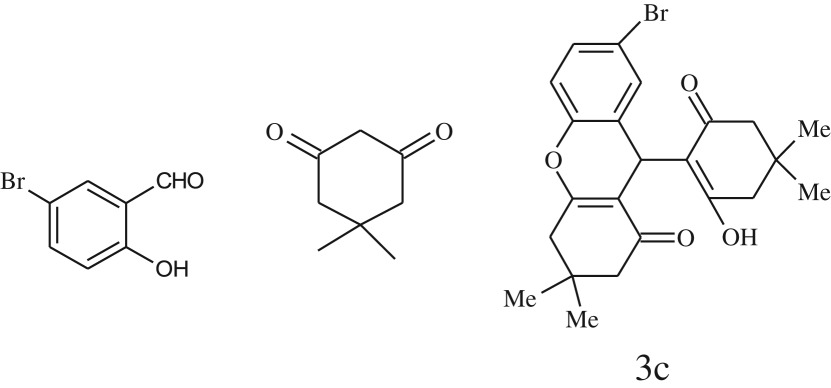			20	92	252–254	251–253 [73]
4	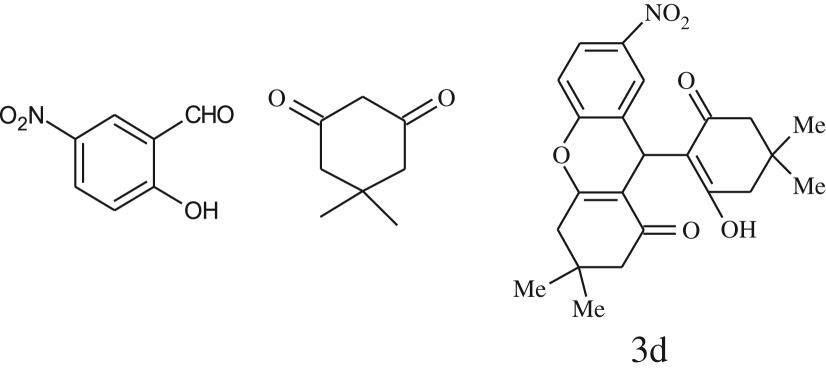			15	94	204–205	203–205 [73]
5	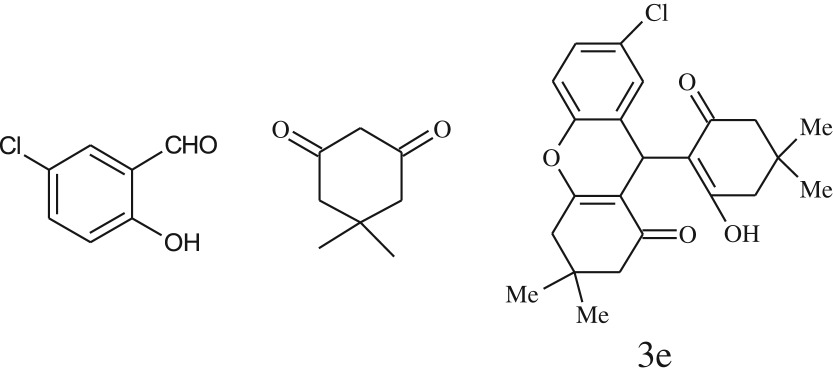			20	92	232–234	236–238 [73]
6	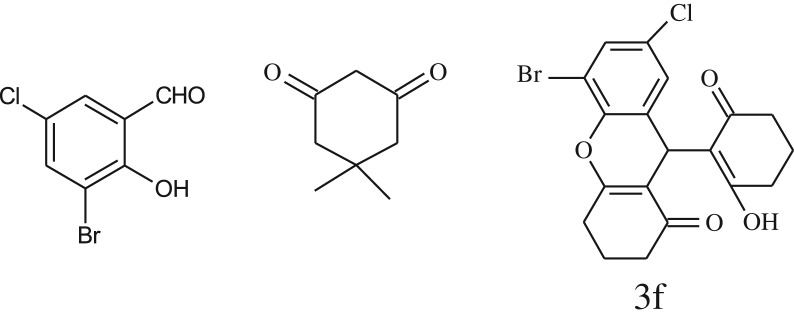			25	84	241–243	240–242 [45]
7	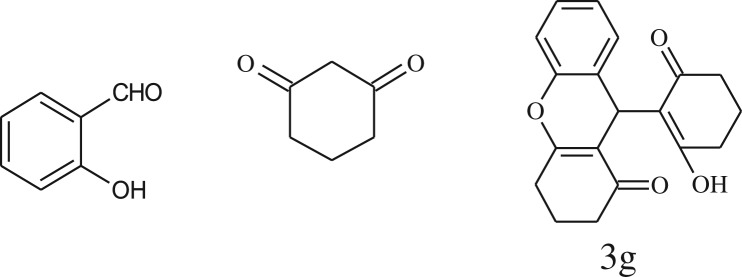			25	90	244–246	245–247 [55]
8	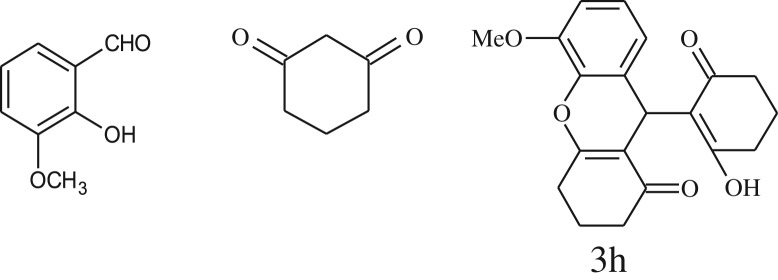			25	87	238–240	238–240 [45]
9	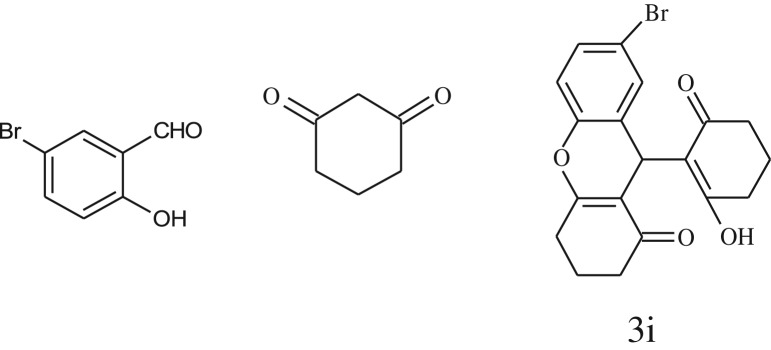			25	88	235–236	234–236 [55]
10	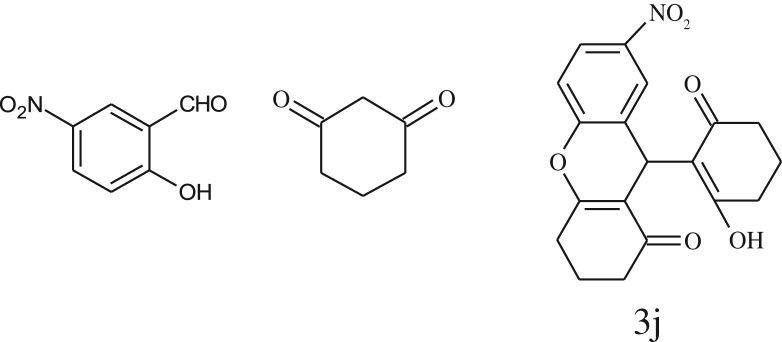			20	93	245–246	242–244 [73]
11	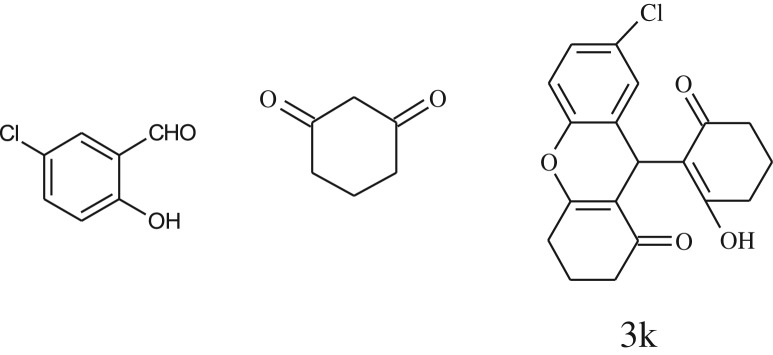			25	92	245–247	244–246 [55]
12	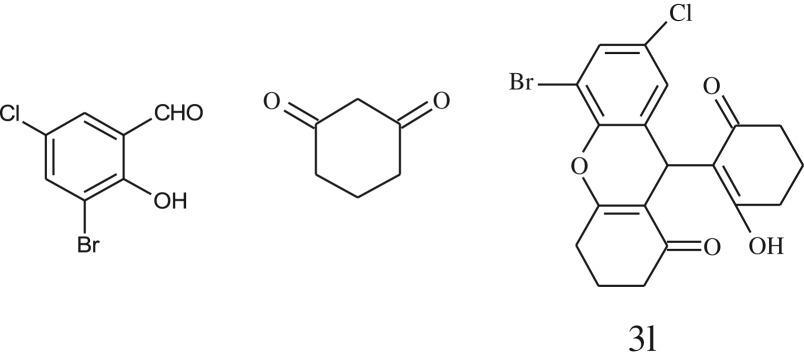			20	91	238–240	238–240 [45]
13	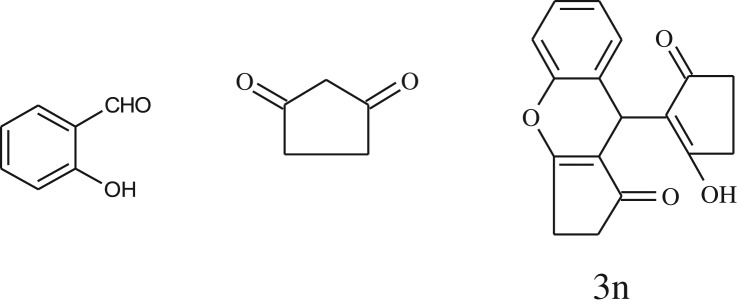			25	89	253–255	254–255 [59]
14	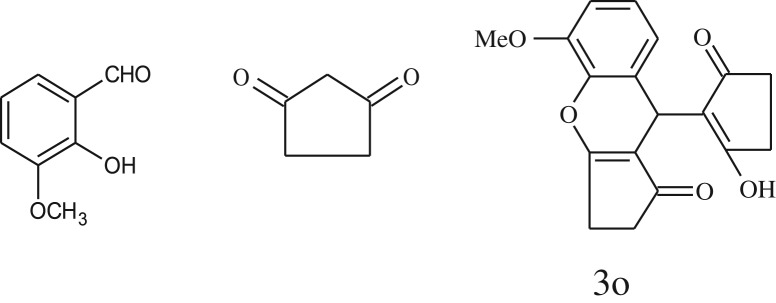			30	87	255–257	255–257 [59]
15	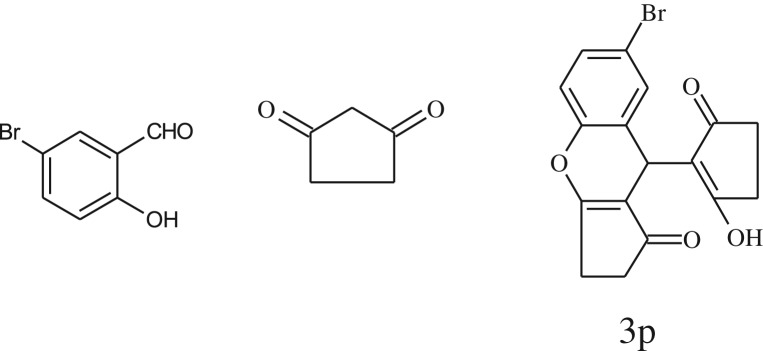			30	92	273–275	272–275 [59]
16	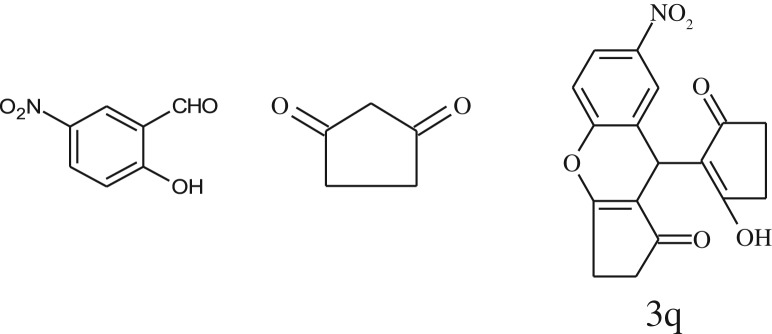			25	93	268–270	266–268 [59]
17	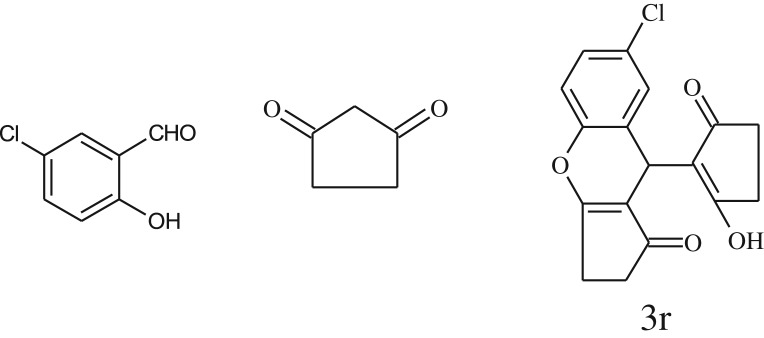			30	91	271–273	271–273 [59]
18	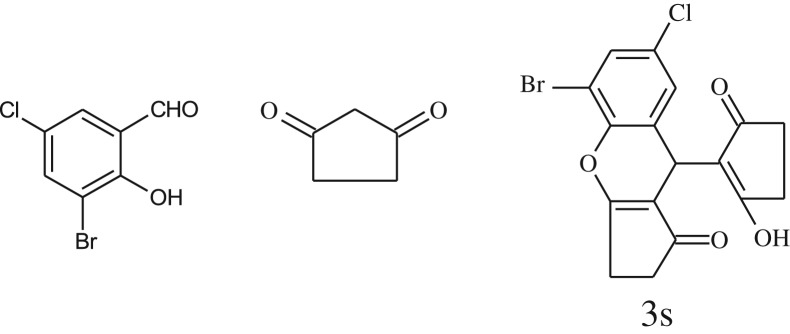			30	84	278–281	277–280 [45]
19	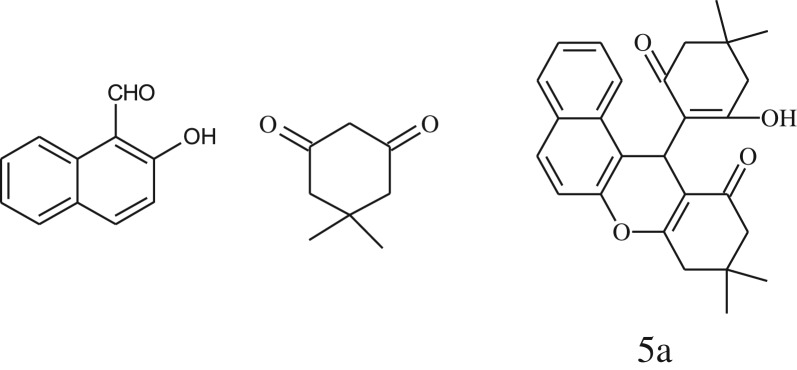			30	92	245–248	246–248 [55]
20	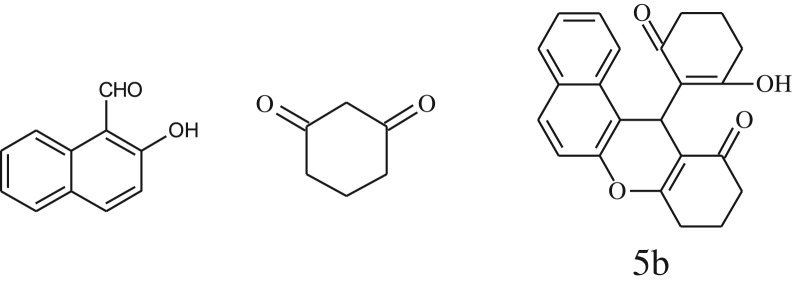			35	91	216–218	216–218 [55]
21	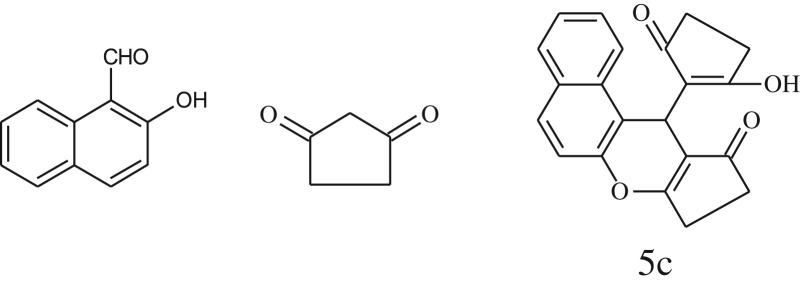			35	90	269–271	270–271 [45]

^a^All reactions were performed by taking salicylaldehydes/2-hydroxy naphthaldehyde (1.0 mmol), 1,3-diketones (2.0 mmol) and CLE-catalyst (0.3 ml), water (5 ml) room temperature.

^b^Isolated yield.

Inspired by these tempting results obtained for cyclocondensation of benzopyran, we extended the same protocol for treating various 1,3-diketones with 2-hydroxy naphthaldehyde (table 3, entry 19–21) and we found that these substrates also worked very efficiently under this catalytic system. As discussed before, in the absence of the catalyst, the reaction proceeded sluggishly, which explains the role of catalytic activity of CLE in product formation.

**Scheme 3.**
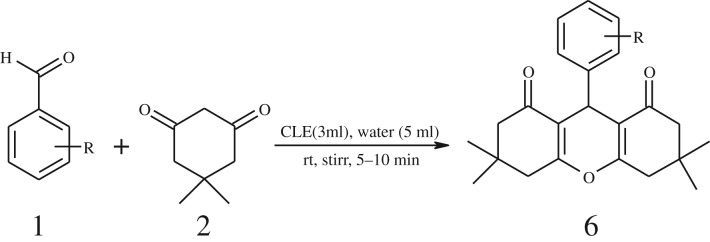
Reaction of aryl aldehydes with 1, 3-dimedone.

Furthermore, when aromatic aldehydes other than 2-hydroxy benzaldehydes were reacted with dimedone in 1 : 2 molar proportion for synthesis of 9-aryl-3,3,6,6-tetramethyl-3,4,5,6,7,9-hexahydro-2*H*-xanthene-1,8-diones under similar reaction conditions (scheme 3), excellent results were obtained in shorter reaction time than previously reported methods. The result of the study is incorporated in table 4.

**Table 4.** Recyclable properties of CLE-catalyst of model reaction. 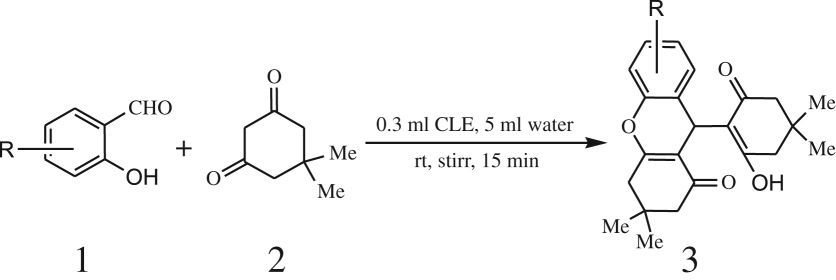

runs	1	2	3	4	5	6	7
yield %	96	96	95	93	92	82	78

### 3.1. Recycle of catalyst

In order to investigate the possibility of recycling this CLE-catalyst, a recycling experiment was conducted using the above-mentioned model reaction (scheme 1). After the separation of the products (see Experimental), the catalyst was treated with activated charcoal (0.05 g) and, after filtration, was used in the next run for synthesis of 3a. Synthesis was performed seven times and the influence of recycling catalyst on yields of 3a is shown in table 4. As can be seen, the CLE as catalyst for pseudo multi-component reaction could be reused at least five times without the apparent loss of catalytic activity, clearly proving its recyclability and reusability. While in sixth and seventh run yield of model reaction was significantly decreased to 82 and 78% respectively.

**Scheme 4.**
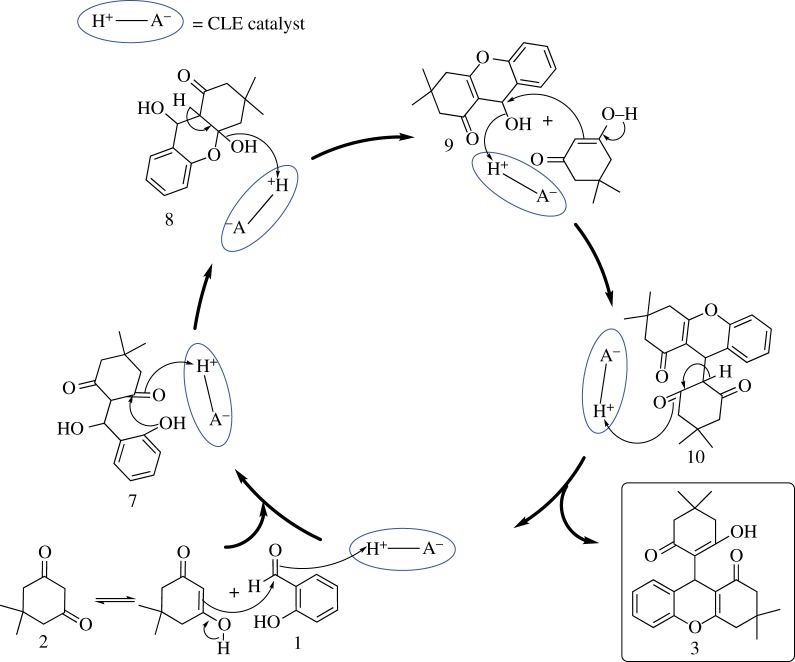
Plausible mechanism for the reaction between salicylaldehyde and 1,3-dimedone catalysed by CLE-catalyst (H^+^--A^−^).

Although diverse approaches towards the synthesis of these derivatives have been developed, use of non-hazardous, biodegradable, natural bio-catalyst is the most elegant strategy. Initially acid-catalysed Knoevenagel condensation between 2-hydroxybenzaldehyde and 1,3-diketone proceeded rapidly within 5 min, which were converted to white product 9, which was further reacted with another molecule of 1,3-diketone with shifting of equilibrium towards formation of desired product 3 (scheme 4) with excellent yield.

Comparison of the experimental results and post-experimental analysis of green parameters using CLE-catalyst with some other reported catalysts for benzopyran synthesis are shown in table 5. It furnishes high reaction yield, takes shorter reaction time, and small quantity of this inexpensive and readily available catalyst is sufficient to get good yield of expected products.

**Table 5.** CLE-catalysed synthesis of 9-aryl-3,3,6,6-tetramethyl-3,4,5,6,7,9-hexahydro-2*H*-xanthene-1,8-diones.^a^ 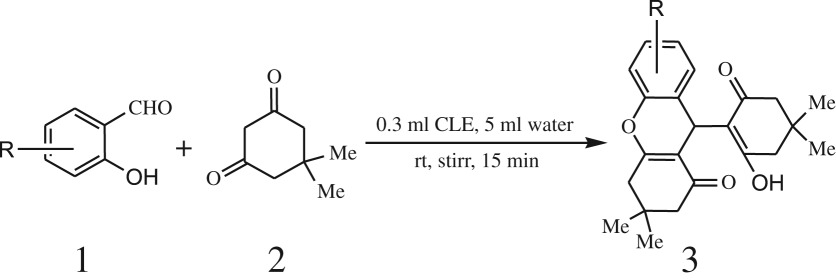

					melting point (°C)	
entry	aldehydes	product	time (min)	yield^b^ (%)	found	reported
1	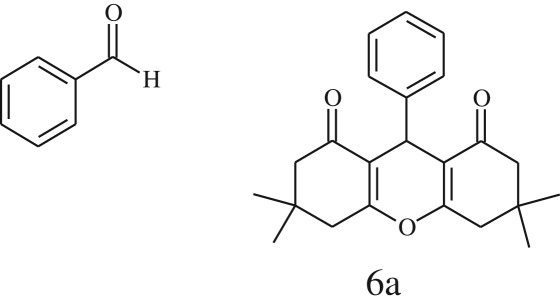		7	94	201–204	200–202 [74]
2	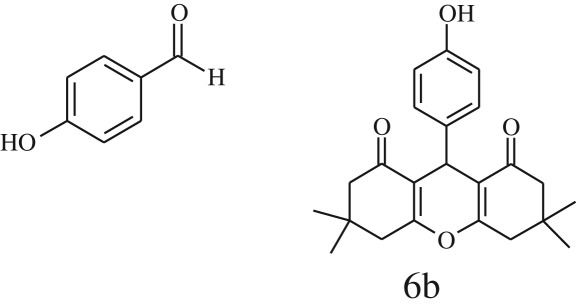		7	94	248–250	245–250 [74]
3	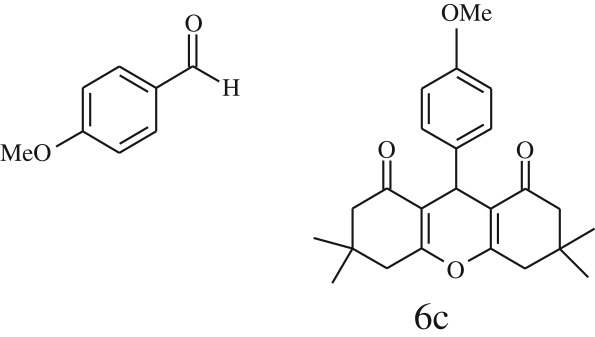		10	96	240–242	241–243 [74]
4	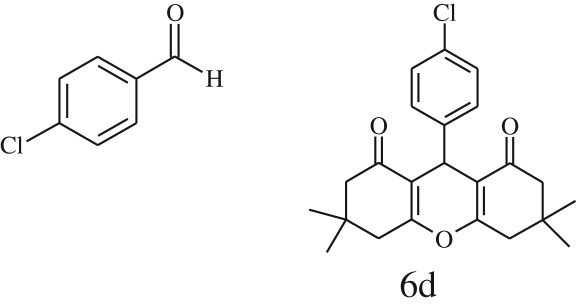		5	95	228–230	228–230 [74]
5	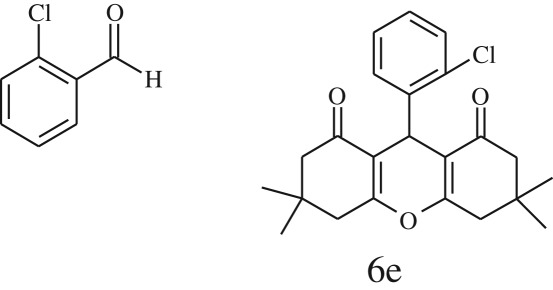		5	95	223–225	225–227 [74]
6	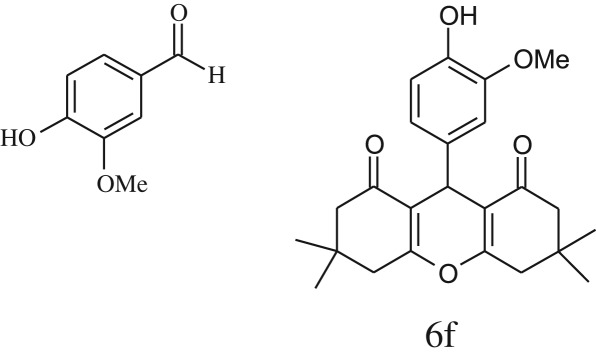		10	93	226–228	226–228 [74]
7	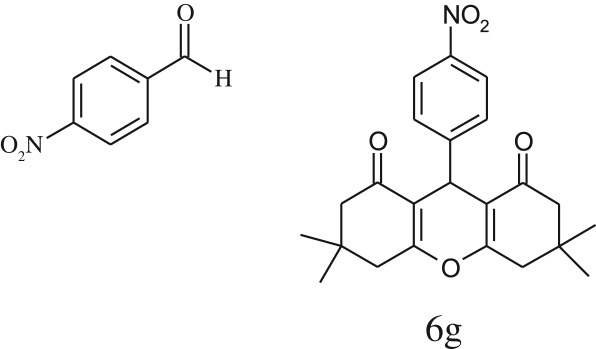		5	99	222–223	222–224 [74]
8	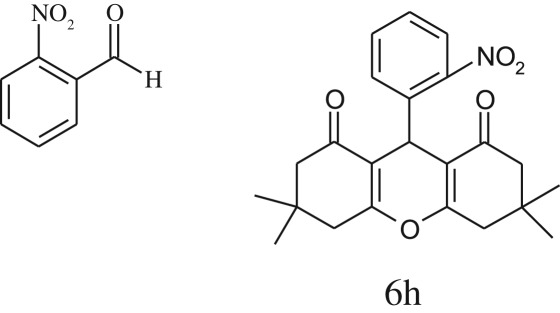		5	95	258–260	258–262 [74]
9	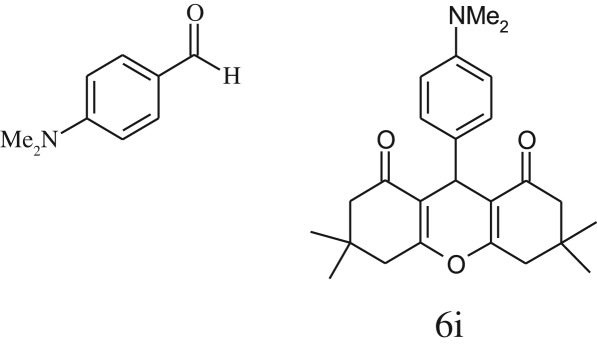		7	96	224–225	222–225 [74]
10	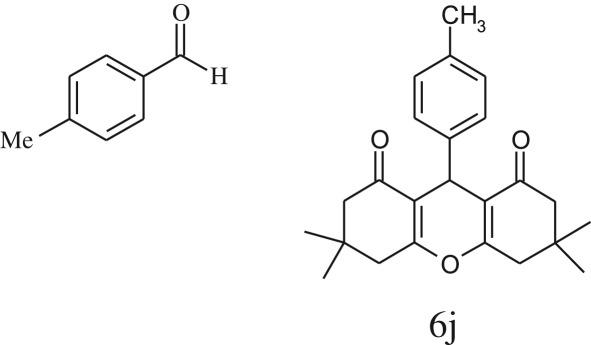		7	91	211–213	210–212 [74]

^a^All reactions were performed by taking aryl aldehydes (1.0 mmol), dimedone (2.0 mmol) and CLE-catalyst (0.3 ml), water (5 ml) room temperature.

^b^Isolated yield.

### 3.2. Post-synthesis analysis

Currently green chemistry metrics and scalability are used to evaluate environmental acceptability and applicability of novel protocols in organic transformations. To address this issue, we calculated green parameters such as atom economy (AE), E-factor, EcoScale and reaction mass efficiency (RME), and these calculated results were compared with results of reported methods. As demonstrated in table 5, the calculated results show that the presented protocol is the greenest among the previously reported methods with respect to green environmental parameters for synthesis of 3a derivative (table 6).

**Table 6.** Comparisons of green chemistry parameters of present method with other reported methods used for synthesis of 3a. 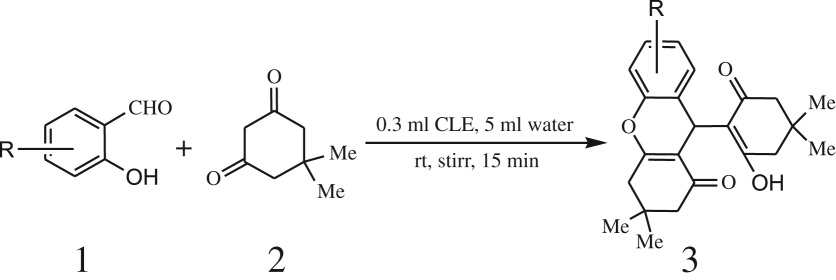

		reaction conditions							
entry	catalyst	solvent (ml)	temp (°C)	time min/h	yield (%)	RME^a^	E-factor (g g^−1^)^b^	Eco Scale^c^	ref.
1	[bmim]PF_6_-glycine	IL	25	3.5 h	82	0.374	0.215	84.00	[60]
2	p-TSA	water	90	30 min	83	0.379	0.200	80.50	[56]
3	[BMim]Br	water	50	15 h	76	0.347	0.311	76.00	[59]
4	TEBA	water	90	05 h	86	0.388	0.776	81.00	[58]
5	cellulose sulfuric acid	solvent free	R.T.	30 min	96	0.437	0.875	89.00	[61]
6	2,4,6-trichloro-1,3,5-triazine	H_2_O	120	2.5 h	93	0.425	0.070	89.50	[55]
7	KF/Al_2_O_3_	EtOH	80	1–3 h	83	0.377	0.207	79.50	[57]
8	CLE^d^	water	rt	15 min	95	0.432	0.051	96.5	—

Equations used for calculations of green parameters:

^a^RME = mass of product/sum of mass of reactants × 100,

^b^E-factor = mass of total waste/mass of product,

^c^EcoScale = 100-sum of individual penalties,

^d^Present method.

## 4. Conclusion

In conclusion, we have an elegant, highly efficient and straightforward bio-organic approach for benzopyran synthesis via Knoevenagel condensation and tandem Knoevenagel–Michael reaction which represents eco-friendly and environmentally benign system. In this article we investigated the synergistic effect of naturally sourced carboxylic acids in heterocyclization using the natural feedstock *chickpea leaf exudates* for the wide range of organic compounds. Our method is flexible, having simple work-up, requires no chromatographic purification, the excellent yields in relatively short-reaction times and at the same time the recyclability and reusability of catalyst, and it can be suitable for one-pot conversion of similar substrates into corresponding products. The use of the low-cost simple-to-handle, biodegradable, naturally sourced catalyst in replacement of toxic synthetic reagents is a promising alternative for the organic transformations. Usability of the catalyst in aqueous medium caters to a more ‘green’ and eco-friendly solution towards benzopyran formation. The catalyst system developed represents the most efficient green protocol so far toward the heterocyclization at room temperature and with excellent values of green chemistry parameters (RME, E-factor, EcoScale) as compared with reported methods, which provides efficient and safe catalyst to the synthetic chemistry community.

## Supplementary Material

Spectra

## Data accessibility

The datasets supporting this article have been uploaded as part of the electronic supplementary material.
