# Supplementary material for: Synergistic effect of natural chickpea leaf exudates acids in heterocyclization: a greener protocol for benzopyran synthesis
Source: R Soc Open Sci. 2018 Feb 7;5(2):170333. doi: 10.1098/rsos.170333 (PMC5830706; doi:10.1098/rsos.170333)
Supplement: Spectra [file rsos170333supp1.docx]

**Synergistic effect of natural Chickpea Leaf Exudates (CLE) acids in heterocyclization: a greener protocol for benzopyran synthesis**

***Snehali Mali, Sachin Shinde, Shashikant Damte, Suresh S. Patil^a*^***

*^a^Synthetic Research Laboratory, PG Department of Chemistry, P.D.V.P. College, Tasgaon, Dist. Sangli, India - 416312.*

*Email: [sanyujapatil@yahoo.com](mailto:sanyujapatil@yahoo.com)

General, procedure for collection of Chickpea Leaf Exudatesand typical procedure for benzopyran formation………………………………………………………S1

Spectroscopic data for products……………………………………………S2 –S24

^1^H NMR, ^13^C NMR spectra of the benzopyran products …….....................S25-S74

General: All commercial reagents and solvents were obtained from *Sigma-Aldrich* and used without purification. Melting points (mp) were determined with DBK-programmable melting point apparatus and are uncorrected. Spots on the TLC plates were visualized using ultraviolet (UV) light. Infrared (IR) spectra were recorded in potassium bromide pellets on a Bruker ALPHA FT-IR spectrometer. The ^1^H NMR spectra (300 MHz) and ^13^C NMR spectra (75.5 MHz) were measured with Avance-300 instrument and chemical shifts (δ) are reported in part per million in CDCl_3_ as a solvent with tetramethylsilane (TMS) as an internal standard. Desired products were obtained only by stirring reaction mixture on Magnetic stirrer (Remi, Model No. 10 ML DX Stirrer). The purity of products and completion of reaction was checked by Thin Layer Chromatography (TLC) on Merck silica gel (60 F_254_) plates. Microanalyses of C, H and N were obtained on a Carl Erba EA 1108. All spectral data (FT-IR, ^1^H and ^13^C) were given in Electronic Supplementary Information (ESI). The pH of *Cicer arietinum*exudates and aqueous solutions of organic acids were measured using Pro-Lab 3000 laboratory pH-meter.

**Procedure for collection of Chickpea Leaf Exudates:**

For quantitative collection of catalyst, *Chickpea* crops cultivated on various lands were selected and then exudates were collected manually using clean cotton napkin by absorption-wringing process (Fig. 2). The quantitative exudates were obtained when it was collected from 7-8 week old cultivated crops at early morning (5-6 am) period. The turbid exudates collected was then filtered through activated charcoal and obtained exudates was freed blur and denominated as CLE-catalyst. The collected catalyst was stored several days at 5 ^o^C and used as a catalyst for benzopyran synthesis.

**Typical procedure for synthesis of 9-(2-hydroxy-4,4-dimethyl-6-oxo-cyclohex-1-enyl)-3,3-dimethyl-2,3,4,9-tetrahydroxanthen-1-one (3a):**

A 25 mL round bottom flask was charged with salicylaldehyde (1.0 mmol), 5,5-dimethyl 1,3-cyclohexanedione (2.0 mmol), CLE-catalyst (0.3 mL) and water (5 mL), and reaction mixture was stirred at room temperature on magnetic stirrer till the completion of reaction as indicated by TLC (ethylacetate:hexane 4:6). The solid products was separated by simple filtration through a Buckner funnel, washed with cold water, and recrystalyzed from 96% ethanol (5 mL). The identity of the compound was ascertained on the basis of FT-IR, ^1^H NMR, and ^13^C NMR spectroscopy (Fig. 3a, 3b & 3c).

-S2-

**9-(2-Hydroxy-4,4-dimethyl-6-oxo-cyclohex-1-enyl)-3,3-Dimethyl-2,3,4,9-tetrahydroxanthen-1-one (3a)**

Yield: 93 %;mp=210-212^o^C; IR (KBr):ν_max_ 3153, 2958, 1622, 1488, 1376, 1312, 1233, 1185 cm^-1^; ^1^H NMR (300 MHz,CDCl_3_): δ10.50 (s, 1H, -OH), 6.93-7.16 (m,4H, Ar-H), 4.65 (s, 1H, -CH), 2.54 (q, J=17.7, 20.0 Hz, 2H, -CH_2_), 2.35 (s, 2H, -CH_2_), 2.30(s, 2H, -CH_2_), 1.93 (q, J=6.0, 16.4 Hz, 2H, -CH_2_), 1.14 (s, 3H, -CH_3_), 1.03 (s,3H, -CH_3_), 0.92-1.23 (s,6H, 2-CH_3_)ppm;^13^C NMR (300 MHz, CDCl_3_): δ200.40, 196.13, 170.53, 168.78, 151.04, 127.98, 127.52, 124.53, 118.31, 115.78, 111.07, 96.20, 50.58, 49.93, 43.24, 41.60, 32.33, 31.02, 29.85, 29.43, 27.79, 27.21,26.42 ppm;MS (ESI): 366(M^+^);Elemental Analysis: Found C, 75.38; H, 7.15; O, 17.46% C_23_H_26_O_4_ requires: C,75.26; H, 7.09%.

-S3-

**3-methoxy-9-(2-hydroxy-4,4-dimethyl-6-oxo-cyclohex-1-enyl)-3,3-dimethyl-2,3,4,9-tetrahydro-xanthen-1-one (Table 3,Entry 2)**

Yield: 86 %;mp=229-231^o^C;IR (KBr): ν_max_3213, 2952, 1641, 1581, 1483, 1375, 1313, 1271, 1230, 1209, 1095, 1024, 758 cm^-1^;^1^H NMR (300 MHz, CDCl_3_): δ10.50 (s, 1H, -OH), 6.93–7.21 (m, 4H, Ar-H), 4.63 (s, 1H, -CH), 2.54 (q, J=17.7, 20.0 Hz, 2H, -CH_2_), 2.35 (s, 2H, -CH_2_), 2.30 (s, 2H, -CH_2_), 1.93 (q, J=6.0, 16.4 Hz, 2H, -CH_2_), 3.88 (s,1H,-OCH_3_) , 1.14(s, 3H, -CH_3_), 0.93 (s, 9H, 3CH_3_)ppm;^13^C NMR (300 MHz, CDCl_3_):δ200.40, 196.521, 170.554, 168.800, 147.081, 140.658, 125.203, 124.203, 119.760, 118.149, 110.887, 110.370, 56.058, 50.623, 49.930, 43.167, 41.533, 32.288, 30.887, 29.852, 29.051, 27.755, 27.162, 26.421ppm;MS (ESI): 396(M^+^);Elemental Analysis: Found C, 72.70; H, 7.12%C_24_H_28_O_5_ requires: C, 72.61; H, 7.21%.

**-S4-**

**7-Bromo-9-(2-hydroxy-4,4-dimethyl-6-oxo-cyclohex-1-enyl)-3,3-dimethyl-2,3,4,9-tetrahydro-xanthen-1-one (Table 3,Entry 3)**

Yield: 92 %;mp=251-253^o^C; IR (KBr): ν_max_ 3103, 2963, 1618, 1475, 1374,1302, 1231, 1178, 1075, 1037, 884, 817, 657, 590, 478 cm^-1^;^1^H NMR (300 MHz, CDCl_3_): δ10.15 (s, 1H, -OH), 7.21–7.24 (dd, J=1.9, 6.8 Hz, 1H, Ar-H), 7.08 (s, 1H, Ar-H), 6.87 (d, J=8.7 Hz, 1H, Ar-H), 5.02 (s, 1H, -CH-), 2.28–2.59 (m, 6H, 3-CH_2_), 1.95 (s, 2H, -CH_2_),1.33 (s, 3H, -CH_3_), 0.99–1.05 (m, 9H, 3-CH_3_) ppm;^13^C NMR (300 MHz, CDCl_3_+DMSO): δ195.65, 164.328, 148.794, 130.563, 129.103, 127.741, 116.932, 115.413, 110.34, 50.27, 40.65, 40.33, 40.05, 39.78, 39.22, 38.94, 38.66, 31.35, 31.27, 28.93,27.56, 26.30 ppm;MS (ESI): 445 (M+1), 447 (M+2);Elemental Analysis: Found C, 62.03; H, 5.66; Br, 17.94; O, 14.37%C_23_H_25_BrO_4_ requires: C, 62.13; H, 5.54; Br, 17.89 %.

**-S5-**

**7-Chloro-9-(2-hydroxy-4,4-dimethyl-6-oxo-cyclohex-1-enyl)-3,3-dimethyl-2,3,4,9-tetrahydro-xanthen-1-one (Table 3,Entry 5)**

Yield: 91 %;mp=232–234^o^C; IR (KBr): ν_max_ 3102, 2965, 2710, 1624, 1571, 1476, 1374, 1301, 1233, 1179, 1077, 1038, 1015, 879, 819, 657, 618, 591, 549, 469 cm^-1^; ^1^H NMR (300 MHz, CDCl_3_): δ10.50(s, 1H, -OH), 7.09 (dd, J=2.2, 2.6 Hz, 1H, Ar-H), 6.91–6.97 (m, 2H, Ar-H), 4.61 (s, 1H, -CH), 2.52 (q, J= 17.3, 18.5 Hz, 2H, -CH_2_), 2.37 (d, J=4.9 Hz, 2H, -CH_2_), 2.30 (s, 2H, -CH_2_), 1.96 (s, 2H, -CH_2_), 1,14 (s, 3H, -CH_3_), 1.00–1.05 (m, 9H, 3-CH_3_)ppm;^13^C NMR (300 MHz, CDCl_3_+DMSO): 198.9, 180.9, 162.2, 152.1, 129.6, 128.4, 126.6, 125.3, 118.5, 115.7, 108.8, 51.6, 46.8, 43.9, 30.5, 30.2, 28.4, 27.5;MS (ESI): 401 (M^+1^) 403 (M+2); Elemental Analysis: Found C, 68.91; H, 6.29; Cl, 8.84; O, 15.96%C_23_H_25_ClO_4_ requires: C, 68.80;Cl,8.74; H, 6.35%.

-**S6-**

**5-Bromo-7-chloro-9-(2-hydroxy-4,4-dimethyl-6-oxo-cyclohex-1-enyl)-3,3-dimethyl-2,3,4,9-tetrahydro-xanthen-1-one (Table 3,Entry 6)**

Yield: 84 %;mp=244-246^o^C;IR (KBr): ν_max_ 3184, 2940, 1647, 1599, 1452, 1375, 1313, 1257, 1207, 1183, 1150, 1017, 887, 855, 803, 722, 662, 587, 475 cm^-1^;^1^H NMR (300 MHz, CDCl_3_ ): δ10.34(s, 1H, -OH), 7.36 (d, J=2.2 Hz, 1H,Ar-H), 6.90 (d, J=2.0 Hz, 1H, Ar-H), 4.60 (s, 1H, -CH), 2.62 (q, J=17.7,18.5 Hz, 2H, -CH_2_), 2.38 (d, J=4.5 Hz, 2H, -CH_2_), 2.31 (s, 2H, -CH_2_), 1.97 (s, 2H, -CH_2_), 1.16 (s, 3H, -CH_3_), 1.00–1.05 (m, 9H, 3-CH_3_) ppm; ^13^C NMR (300 MHz, CDCl_3_): δ196.47, 195.10, 163.43, 144.77, 128.68, 127.79, 127.41, 126.35, 112.78,109.93, 108.78, 94.79, 49.58, 49.03, 39.81, 30.77, 28.24, 26.92, 26.03, 25.64, 25.17 ppm; MS (ESI): 479 (M+1), 481 (M+2);Elemental Analysis: Found C, 57.58; H,5.04; Br, 16.65; Cl, 7.39; O, 13.34%C_23_H_24_BrClOrequires: C, 57.49, Cl, 7.39; Br, 16.57; H, 5.11%.

**-S7-**

**-(2-Hydroxy-6-oxo-cyclohex-1-enyl)-2,3,4,9-tetrahydro-xanthen-1-one(Table 3, Entry 7**)

Yield: 87 %;mp=245-247^o^C; IR (KBr): ν_max_ 2951, 2538, 1830, 1641, 1553, 1485, 1421, 1372, 1294, 1235, 1192, 1142, 1071, 993, 924, 850, 773, 564, 493 cm^-1^; ^1^HNMR (300 MHz, CDCl_3_): δ10.00 (s, 1H, -OH), 6.80–7.14 (m, 4H, Ar-H), 4.84(s, 1H, -CH), 1.60–2.40 (m, 12H, 6-CH_2_) ppm;^13^C NMR (300 MHz, CDCl_3_): δ 198.9, 180.9, 162.2, 154.0, 129.8, 126.5, 124.0, 122.9, 117.1, 116.7, 108.8, 36.8, 31.6, 28.7, 19.2, 18.9 ppm; MS (ESI): 311 (M+1);Elemental Analysis: Found C, 73.53; H, 5.85; O, 20.62%C_19_H_18_O_4_ requires: C, 73.45; H, 5.74%.

**-S8-**

**7-Bromo-9-(2-hydroxy-6-oxo-cyclohex-1-enyl)-2,3,4,9-tetrahydro-xanthen-1-one (Table 3, Entry 9)**

Yield: 88 %;mp=238-239^o^C; IR (KBr): ν_max_3105, 2955, 1640, 1596, 1477, 1374,1279, 1233, 1186, 1144, 1070, 981, 819, 763, 620, 530, 470 cm^-1^;^1^H NMR (300 MHz): δ10.75 (s, 1H, -OH), 7.25 (d, J=3.0 Hz, 1H, Ar-H), 7.09 (d, J=2.2 Hz, 1H, Ar-H), 6.89 (dd, J=5.2, 6.0 Hz, 1H, Ar-H), 4.57(s, 1H, -CH), 1.76–2.85 (m, 12H, 6-CH_2_) ppm;^13^C NMR (300 MHz,CDCl_3_): δ 202.18, 193.99, 167.43, 166.15, 148.45, 129.74, 129.28, 128.61, 126.86, 116.51, 116.10, 115.09, 47.57, 35.57, 35.02, 33.26, 26.02, 22.80, 18.95 ppm;MS (ESI): 389 (M+1) 391 (M +2 );Elemental Analysis: FoundC, 58.63; H, 4.40; Br, 20.53; O, 16.44%C_19_H_17_BrO_4_ requires:C, 58.54; Br, 20.44; H, 4.32%.

**-S9-**

**7-Chloro-9-(2-hydroxy-6-oxo-cyclohex-1-enyl)-2,3,4,9-tetrahydro-xanthen-1-one(Table3, Entry 11 )**

Yield: 94 %;mp=244-246^o^C; IR (KBr): ν_max_ 3110, 2954, 1645, 1596, 1477, 1416,1375, 1280, 1239, 1188, 1141, 1068, 984, 917, 824, 576, 460 cm^-1^;^1^H NMR (300MHz, CDCl_3_): δ10.76 (s, 1H, -OH), 7.09 (d, J=6.3 Hz, 1H, Ar-H),6.91–6.96 (m, 2H, Ar-H), 4.57(s, 1H, -CH), 1.77–2.81 (m, 12H, 6-CH_2_) ppm;^13^C NMR (300MHz, CDCl_3_):δ 201.86, 193.94, 167.23, 165.96, 147.56,127.13, 126.76, 125.61, 124.53, 115.52, 110.65, 99.74, 47.08, 35.47, 34.82, 33.05, 26.35, 22.69, 19.03 ppm;MS (ESI): 345 (M+1), 347 (M + 2); Elemental Analysis: Found C, 66.19; H, 4.97; Cl, 10.28; O, 18.56%C_19_H_17_ClO_4_ requires: C, 66.08;Cl, 10.19; H, 4.88%.

**-S10-**

**5-Bromo-7-chloro-9-(2-hydroxy-6-oxo-cyclohex-1-enyl)-2,3,4,9-tetrahydro-xanthen-1-one(Table 3, Entry 12)**

Yield: 91 %;mp=238–240^o^C;IR (KBr): ν_max_49, 2887, 2526, 1651, 1560, 1452, 1363, 1279, 1245, 1185, 1133, 1063, 1007, 857, 765, 707, 538, 500, 438 cm^-1^;^1^H NMR (300MHz, CDCl_3_): δ10.44 (s, 1H, -OH), 7.30 (d, J=2.6 Hz, 1H, Ar-H), 6.95 (d, J=1.7 Hz, 1H, Ar-H), 5.04 (s, 1H, -CH), 1.93–2.12 (m, 4H, 2-CH_2_),2.25–2.51(m,8H,4-CH_2_)ppm;^13^CNMR (300MHz,CDCl_3_):δ 195.44, 163.90, 145.45, 129.31, 128.72, 127.93, 110.62, 109.50, 50.29, 40.33, 40.05, 39.77, 39.50,39.2, 38.94, 38.76,31.45,31.3,28.97,27.56,26.69,26.20,25.75, 29 ppm;MS (ESI): 423 (M+1), 425 (M+2);Elemental Analysis: Found C, 53.86; H, 3.81; Br, 18.86; Cl, 8.37; O, 15.11%C_19_H_16_BrClO_4_ requires: C, 53.75; H, 3.70; Br, 18.72; Cl, 8.25%.

**-S11-**

**5-methoxy-2,3-Dihydro-9-(2-hydroxy-5-oxocyclopent-1-enyl)-cyclopenta[*b*]chromen-1(9*H*)-one (Table 3, Entry 14)**

Yield: 84%;mp=255-257^o^C;IR (KBr): ν_max_3438, 3024, 2971, 2939, 1682, 1637, 1579, 1480, 1445, 1380, 1322, 1273, 1255, 1237, 1170, 1125, 1076, 825, 788, 739, 716cm^-1^;^1^H NMR (300 MHz DMSO): δ 2.29-2.36 (m, 6H, 3CH_2_), 2.72~2.74 (m, 2H, CH_2_), 3.82 (s, 3H, CH_3_O), 4.58 (s, 1H, CH), 6.58~6.60 (m, 1H, ArH), 6.92 (dd, *J* = 8.0 Hz, *J*= 1.2 Hz, 1H, ArH), 6.99-7.03 (m,1H, ArH), 11.80 (b, 1H, OH);^13^C NMR (300 MHz, CDCl_3_): δ 194.0, 169.5, 152.8, 130.0, 123.9, 120.8, 112.2, 56.2, 32.9, 31.0, 30.7, 27.9, 23.7 ppm;MS (ESI): 312(M+1);Elemental Analysis: Found C, 69.22; H, 5.16; O, 25.61% C_18_H_16_O_5_ requires:C, 69.13; H, 5.07%.

**-S12-**

**7-Bromo-2,3-dihydro-9-(2-hydroxy-5-oxocyclopent-1-enyl)cyclopenta[*b*]chromen-1(9*H*)-one(Table 3, Entry 15)**

Yield: 90%;mp=280-282^o^C;^1^H NMR (300 MHz DMSO): δ 2.26~2.43 (m, 6H, 3CH_2_), 2.56~3.34(m, 2H, CH_2_), 5.01(s, 1H, CH), 7.01~7.24 (m, 2H, ArH), 7.27 (d, *J* = 8.4 Hz, 1H, ArH), 10.60 (b, 1H, OH);IR (KBr): ν_max_3505, 2932 , 2910, 1699, 1653, 1585, 1474, 1383, 1276, 1259, 1240,1198, 1160, 1126, 1071, 1018, 818, 707, 659 cm^-1^;^13^C NMR (300 MHz, CDCl_3_): δ 194.0,133.2, 131.0, 129.6, 119.3, 117.2, 109.3, 106.0, 32.9, 31.0, 30.7, 27.9, 22.7 ppm;MS (ESI): 361 (M+1) 363 (M+2);Elemental Analysis: Found C, 56.53; H, 3.63; Br, 22.12; O, 17.72%C_17_H_13_BrO_4_ requires: C, 56.34; H, 3.45; Br, 22.22%.

**-S13-**

**7-nitro-2,3-Dihydro-9-(2-hydroxy-5-oxocyclopent-1-enyl)cyclopenta[*b*]chromen-1(9*H*)-one (Table 3, Entry 16)**

Yield: 93 %;mp=268-270^o^C;IR (KBr): ν_max_2926, 1698, 1656, 1581, 1528, 1481, 1458, 1379, 1277, 1253, 1168, 1134, 1020, 929, 912, 840, 805, 748, 666 cm^-1^; ^1^H NMR (300 MHz DMSO ): δ 2.34~2.41 (m, 6H, 3CH_2_), 2.74~2.76 (m, 2H, CH_2_), 4.72(s, 1H, CH), 7.41 (d, *J* = 8.8 Hz, 1H, ArH), 7.87 (d, *J* =2.4Hz, 1H, ArH), 8.11 (dd, *J*=8.8 Hz, *J*= 2.4 Hz, 1H,ArH), 12.06 (b, 1H, OH);^13^C NMR (300 MHz, CDCl_3_): δ 206.0, 201.2, 170.3, 154.0, 129.8, 126.5, 122.9, 117.1,110.4, 103.5, 31.1,30.9,26.5 ppm; MS (ESI): 327 (M+1);Elemental Analysis: Found C, 62.39; H, 4.00; N,4.28; O, 29.33% C_17_H_13_NOrequires: C, 62.21; H, 4.11; N, 4.19%.

**-S14-**

**7-Chloro-2,3-dihydro-9-(2-hydroxy-5-oxocyclopent-1-enyl)cyclopenta[*b*]chromen-1(9*H*)-one(Table 3, Entry 17)**

Yield: 94 %;mp=271-274^o^C; IR (KBr): ν_max_3508, 2935, 2914, 1699, 1654, 1583, 1477, 1409, 1384, 1277, 1259, 1240, 1162,1126, 1018, 819, 677 cm^-1^;^1^H NMR (300 MHz DMSO): δ 2.33~2.38 (m, 6H, 3CH_2_), 2.71~2.73 (m, 2H, CH_2_), 4.60(s, 1H, CH), 7.01 (dd, *J* = 2.4 Hz, *J*= 1.2 Hz, 1H, ArH),7.18 (d, *J* = 8.8 Hz, 1H, ArH), 7.27~7.30 (m, 1H, ArH),12.00 (d, 1H, OH);^13^C NMR (300 MHz, CDCl_3_): δ 206.0, 201.2, 170.3, 154.0, 129.8, 126.5, 122.9, 117.1, 110.4, 103.5, 31.1,30.9,26.5 ppm;MS (ESI): 316 (M+1), 318 (M + 2);Elemental Analysis: Found C, 64.47; H, 4.14; Cl, 11.19; O, 20.20% C_17_H_13_ClOrequires: C, 64.38; H, 4.25;Cl, 11.09%.

**-S15-**

**3,3,6,6-Tetramethyl-9-benzene-1,8-dioxo-octahydroxanthene(6a)**

Yield: 84%;mp=255-257^o^C. IR (KBr): ν_max_3032, 2984, 1686, 1673, 1471, 1365, 1197, 1174, 1142, 1009, 745, 700 cm^-1^;^1^H NMR (CDCl_3_): δ 0.97 (s, 6 H, 2 × CH_3_), 1.11 (s, 6 H, 2 × CH_3_), 2.22 (dd, 4 H, *J* = 1.6 Hz, *J* = 2.4 Hz, 2× CH_2_, H-4, H-5), 2.47(s, 4 H, 2 ×CH2, H-2, H-7), 4.68 (s, 1 H, H-9), 7.23 (m, 5 H, Ar-H) ppm;^13^C NMR (300 MHz, CDCl_3_): δ 198.9, 155.1, 142.2, 129.4, 129.1, 128.7, 125.8, 113.9, 51.6, 44.6, 39.1, 30.6, 27.5 ppm;MS (ESI): 336 (M+1);Elemental Analysis: Found C, 78.54; H, 7.19; O, 14.27%C_22_H_24_O_3_ requires: C, 78.45; H, 7.27%.

**-S16-**

**3,3,6,6-Tetramethyl-9-(4-hydroxyphenyl)-1,8-dioxo-octahydroxanthene(6b)**

IR (KBr): ν_max_ 3362, 3027, 2982, 1797, 1727, 1702, 1634, 1615, 1527, 1392, 1377, 1263, 1235, 1203, 1197, 851, 845 cm^–1^;^1^H NMR (CDCl_3_): δ 0.98 (s, 6H, 2× CH3), 1.12 (s, 6 H, 2 × CH_3_), 2.15 (dd, 4 H, *J* = 1.6 Hz, *J* = 4.0 Hz, 2×CH2, H-4, H-5), 2.48 (s, 4 H, 2×CH2, H- 2, H-7), 4.65 (s, 1 H, H-9), 6.79 (d, 2 H, *J* = 8.0 Hz, ArH), 6.99 (d, 2 H, *J* = 8.0, ArH) ppm;^13^C NMR (300 MHz, CDCl_3_): δ 198.9, 155.5, 155.1, 134.8, 130.5, 115.8, 113.9, 51.6, 44.6, 39.1, 30.6, 27.5 ppm;MS (ESI): 352 (M+1);Elemental Analysis: Found C, 74.98; H, 6.86; O, 18.16%C_22_H_24_O_4_ requires: C, 74.81; H, 6.77%.

**-S17-**

**3,3,6,6-Tetramethyl-9-(4-methoxyphenyl)-1,8-dioxo-octahydroxanthene(6c)**

IR (KBr): ν_max_ 3027, 2981, 1687, 1662, 1621, 1515, 1451, 1377, 1362, 1262, 1237, 1172, 1145, 1035, 1005, 844cm^–1^;^1^H NMR (CDCl_3_): δ 1.02 (s, 6 H, 2×CH_3_), 1.10 (s, 6 H, 2× CH3), 2.205(dd, 4 H, *J* = 1.6 Hz, *J* = 2.0 Hz, 2CH2, H-4, H-5), 2.47 (s, 4 H, 2×CH2, H-2, H-7), 3.75 (s, 3H, CH3O), 4.72(s, 1H, H-9), 6.68–7.29 (m, 4H, ArH) ppm; ^13^C NMR (300 MHz, CDCl_3_): δ 198.7, 157.7, 155.1, 134.5, 130.1,114.2, 113.9, 55.9, 51.6, 44.6, 39.1, 30.6, 27.5 ppm;MS (ESI): 380 (M+1);Elemental Analysis: Found C, 75.76; H, 7.42; O, 16.82%C_24_H_28_O_4_ requires: C, 75.68; H, 7.23%.

**-S18-**

**3,3,6,6-Tetramethyl-9-(4-chlorophenyl)-1,8-dioxo-octahydroxanthene.(6d)**

IR (KBr): ν_max_ 3027, 2982, 1681, 1663, 1621, 1494, 1481, 1363, 1199, 1171, 1142, 1094, 1011, 1002, 855, 844 cm^–1^;^1^H NMR (CDCl_3_): δ 0.97 (s, 6 H, 2×CH3), 1.11 (s, 6 H, 2× CH_3_), 2.25(dd, 4 H, *J* = 1.6 Hz, *J* = 3.6 Hz, 2×CH_2_, H-4, H-5), 2.51 (s, 4 H, 2 CH_2_, H-2, H-7), 4.65 (s, 1 H, H-9), 7.27–7.45 (m, 4 H, ArH);^13^C NMR (300 MHz, CDCl_3_): δ 198.8, 155.1, 140.3, 131.5, 130.5, 128.8, 113.9, 51.6, 44.6, 30.6, 27.5 ppm; MS (ESI): 384 (M+1).386(M + 2); Elemental Analysis: Found C, 71.77; H, 6.55; Cl, 9.21; O, 12.47%C_23_H_25_ClO_3_ requires: C, 71.68; H, 6.46%.

**-S19-**

**3,3,6,6-Tetramethyl-9-(2-chlorophenyl)-1,8-dioxo-octahydroxanthene(6e)**

IR (KBr): ν_max_ 3033, 2982, 1681, 1667, 1621, 1497, 1472, 1361, 1202, 1171, 1145, 1101, 1005, 851, 842cm^-1^.;^1^H NMR (CDCl_3_): δ 0.99 (s, 6 H, 2×CH_3_), 1.12 (s,6 H, 2×CH_3_), 2.07 (dd, 4 H, *J* = 1.6 Hz, *J* = 3.0 Hz,2×CH_2_, H-4, H-5), 2.51 (s, 4 H, 2×CH_2_, H-2, H-7), 4.67 (s,1 H, H-9), 7.27–7.36 (m, 4 H, ArH);^13^C NMR (300 MHz, CDCl_3_): δ 198.7, 155.2, 143.6, 134.4, 130.6, 128.9, 127.2, 113.8, 51.7, 44.7, 30.0, 27.6 ppm; MS (ESI): 384 (M+1), 386 (M + 2);Elemental Analysis: Found C, 71.77; H, 6.55; Cl, 9.21; O, 12.47%C_23_H_25_ClO_3_ requires: C, 71.69;H, 6.47; Cl, 9.11%.

**-S20-**

**3,3,6,6-Tetramethyl-9-(4-hydroxy-3-methoxyphenyl)-1,8-dioxo-octahydroxanthene(6f)**

IR (KBr): ν_max_ 3445, 3031, 2987, 2197, 1686, 1662, 1582, 1502, 1455, 1402, 1235,1197, 1042, 815, 745 cm^–1^; ^1^H NMR (CDCl_3_): δ 1.01 (s, 6H, 2×CH_3_), 1.11 (s, 6 H, 2×CH_3_), 2.25 (d, 4 H, *J* = 3.6 Hz, 2×CH_2_, H-4, H-5), 2.49 (s, 4 H, 2×CH_2_, H-2, H-7), 3.95 (s, 3 H, OCH3), 4.69 (s, 1 H, H-9), 5.48 (s, 1 H, OH), 6.65 (s, 1H, ArH), 6.75 (s, 1 H, ArH), 7.05 (s, 1 H, ArH); ^13^C NMR (300 MHz, CDCl_3_): δ 198.7, 155.1, 151.3, 142.7, 135.8, 122.8, 116.8, 114.5, 133.9, 56.2, 51.6, 44.6, 30.6, 27.5 ppm;MS (ESI): 396 (M+1);Elemental Analysis: Found C, 72.71; H, 7.12; O, 20.18%C_24_H_28_O requires: C, 72.63; H, 7.03%.

**-S21-**

**3,3,6,6-Tetramethyl-9-(4-nitrophenyl)-1,8-dioxo-octahydroxanthene(6g)**

IR (KBr): ν_max_ 3035, 2984, 1667, 1651, 1625, 1531, 1361, 1344, 1201, 1072, 1043, 1007, 876, 835cm^-1^;^1^H NMR (CDCl_3_): δ 1.10 (s, 6 H, 2 CH3), 1.10 (s, 6 H, 2×CH_3_), 2.14 (dd, 4 H, *J* = 1.6 Hz, *J* = 3.6 Hz, 2×CH_2_, H-4, H-5), 2.42 (s, 4 H, 2×CH_2_, H-2, H-7), 4.47 (s, 1 H, H-9),7.51–7.65 (m, 2 H, ArH), 8.07–8.17 (m, 2 H, ArH);^13^C NMR (300 MHz, CDCl_3_): δ 198.9, 155.1, 148.4, 145.3, 130.0, 121.0, 113.9, 51.6, 46.6, 30.6, 27.5 ppm; MS (ESI): 395 (M+1);Elemental Analysis: Found C, 69.86; H, 6.37; N, 3.54; O, 20.23%C_23_H_25_NO_5_ requires: C, 69.77; H, 6.28; N, 3.44%.

**-S22-**

**3,3,6,6-Tetramethyl-9-(2-nitrophenyl)-1,8-dioxo-octahydroxanthene(6h)**

IR (KBr): ν_max_ 3037, 2981, 1687, 1675, 1621,1535, 1361, 1212, 1175, 1154, 1011, 865, 810, 781, 747, 705 cm^–1^;^1^H NMR (CDCl_3_): δ 0.97 (s, 6 H, 2×CH_3_), 1.11 (s,6 H, 2×CH_3_), 2.18 (dd, 4 H, *J* = 1.6 Hz, *J* = 3.6 Hz, 2×CH_2_, H-4, H-5), 2.44 (s, 4 H, 2×CH_2_, H-2, H-7), 5.65 (s,1 H, H-9), 7.37–7.46 (m, 4 H, ArH);^13^C NMR (300 MHz, CDCl_3_): δ 198.9, 155.1, 149.1, 134.8, 131.9, 130.0, 126.7, 121.0, 113.9, 51.6, 44.6, 30.6, 27.5 ppm;MS (ESI): 395 (M+1); Elemental Analysis: Found C, 69.86; H, 6.37; N, 3.54; O, 20.23%, C_23_H_25_NO_5_ requires: C, 69.77; H, 6.48; N, 3.46%.

**-S23-**

**3,3,6,6-Tetramethyl-9-(4-dimethylaminophenyl)-1,8-dioxo-octahydroxanthene(6i)**

IR (KBr): ν_max_ 3037, 2981, 2197,1688, 1667, 1581, 1502, 1455, 1408, 1236, 1197, 1041, 815, 745 cm^–1^;^1^H NMR (CDCl_3_): δ 1.03 (s, 6 H, 2×CH_3_), 1.12(s, 6 H, 2×CH_3_), 2.23 (dd, 4 H, *J* = 1.6 Hz, *J* = 2.4 Hz, 2×CH_2_, H-4, H-5), 2.47 (s, 4 H, 2×CH_2_, H-2, H-7), 2.89 [s,6 H, -N(CH_3_)_2_], 4.68 (s, 1H, H-9), 6.65 (s, 2H, ArH), 7.17 (s, 2H, ArH);^13^C NMR (300 MHz, CDCl_3_): δ 198.8, 155.1, 146.6, 140.3, 131.7, 130.0, 113.9, 51.6, 44.6, 30.6, 27.5 ppm;MS (ESI): 393 (M+1);Elemental Analysis: Found C, 76.30; H, 7.94; N, 3.56; O, 12.20%C_25_H_31_NO_3_ requires: C, 76.21; H, 7.75;N, 3.47%.

**-S24-**

**3,3,6,6-Tetramethyl-9-(4-methylphenyl)-1,8-dioxo-octahydroxanthene(6j)**

IR (KBr): ν_max_ 3036, 2981, 1686, 1667, 1635, 1516, 1472, 1366, 1202, 1167, 1141, 791, 776 cm^-1^; ^1^H NMR(CDCl_3_): δ 1.02 (s, 6 H, 2×CH_3_), 1.10 (s, 6 H, 2×CH_3_), 2.06 (dd, 4 H, *J* = 1.6 Hz, *J* = 2.4 Hz, 2×CH_2_, H-4, H-5),2.41 (s, 4 H, 2×CH_2_, H-2, H-7), 2.45 (s, 3 H, CH_3_Ar), 4.70 (s, 1H, H-9), 6.81–7.29 (m, 4 H, ArH);^13^C NMR (300 MHz, CDCl_3_): δ 198.9, 155.1, 139.2, 135.4, 129.0, 113.9, 51.6, 44.6, 30.6, 27.5, 24.3 ppm; MS (ESI): 364 (M+1);Elemental Analysis: Found C, 79.09; H, 7.74; O, 13.17%C_24_H_28_O_3_ requires: C, 79.17; H, 7.55%.

| **S25-** |
| --- |
| 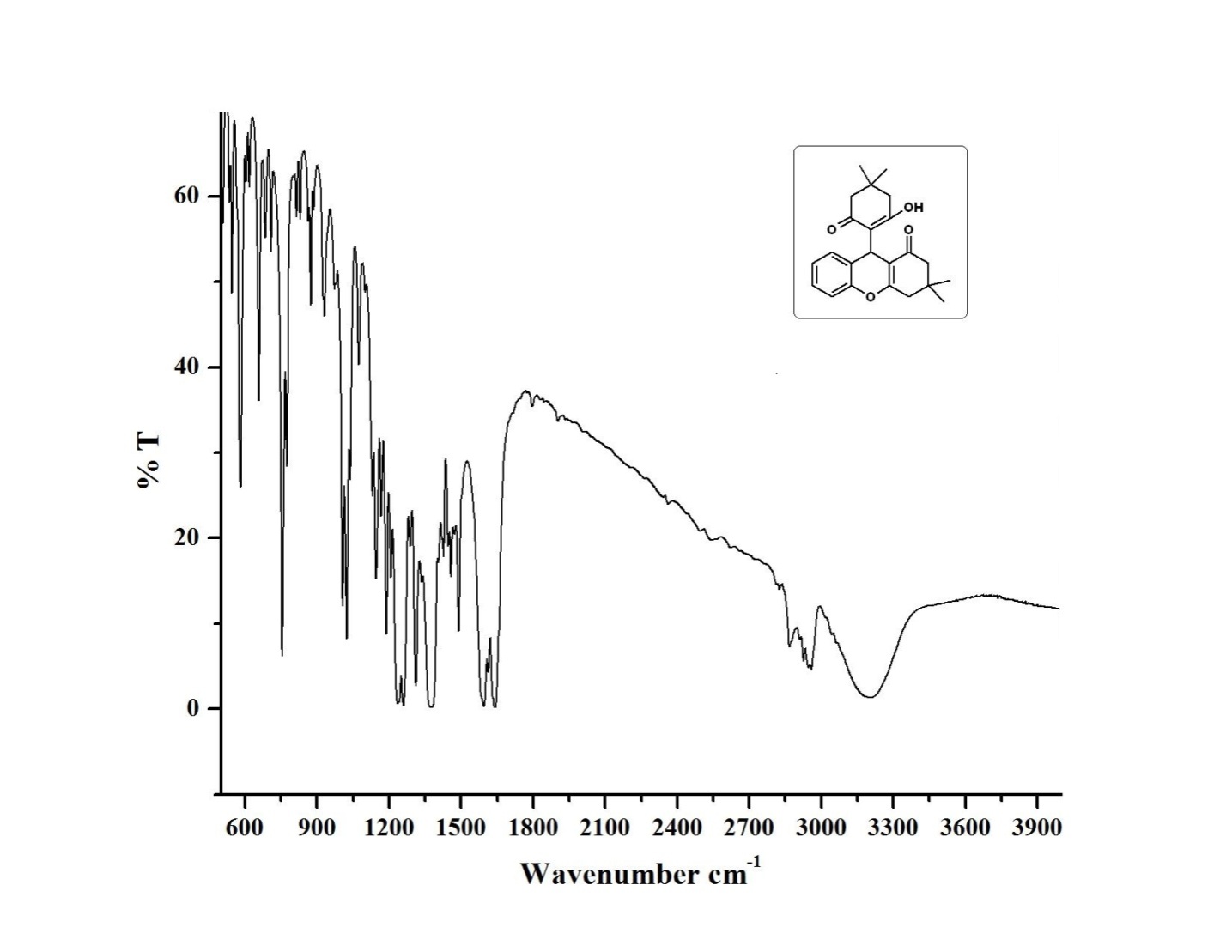 |
| Supplementary Figure S25 from FT-IR spectrum of 3a |
| **-S26-** |
| 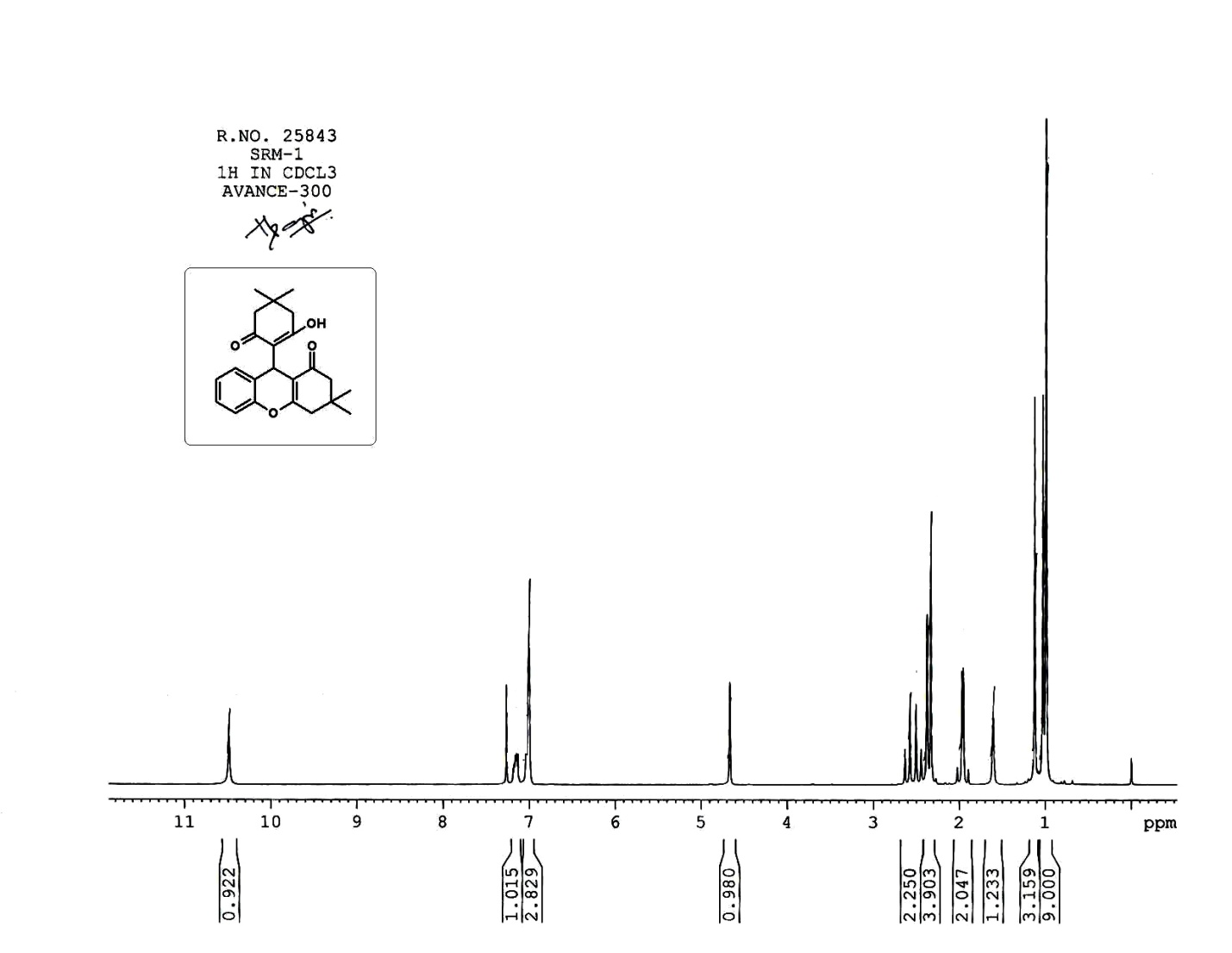 |
| Supplementary Figure S26 from ^1^HNMRspectrum of compound 3a |
| **-S27-** |
| 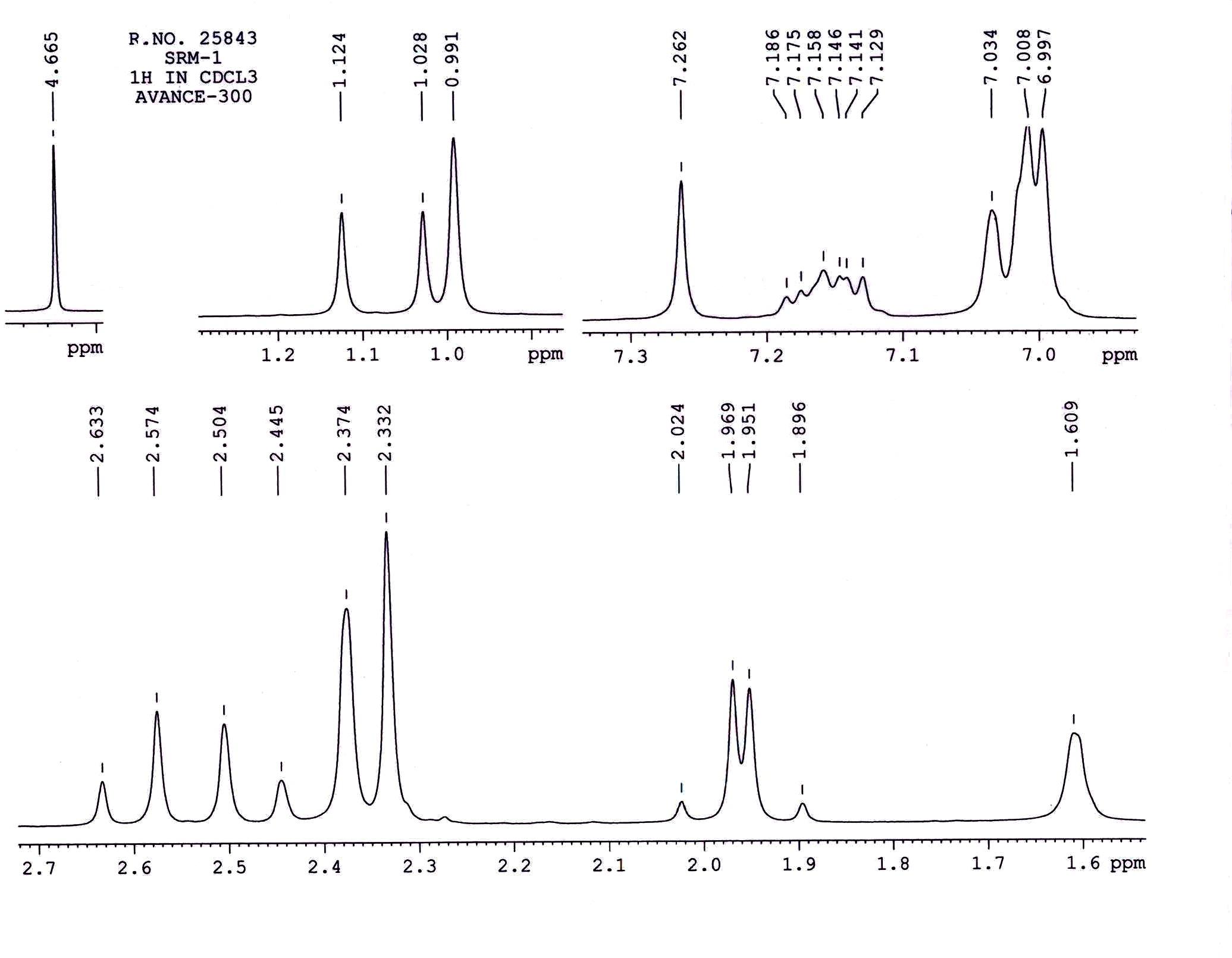 |
| Supplementary Figure S27 from Resolved ^1^HNMR spectrum of compound of 3a |
| **-S28-** |
| 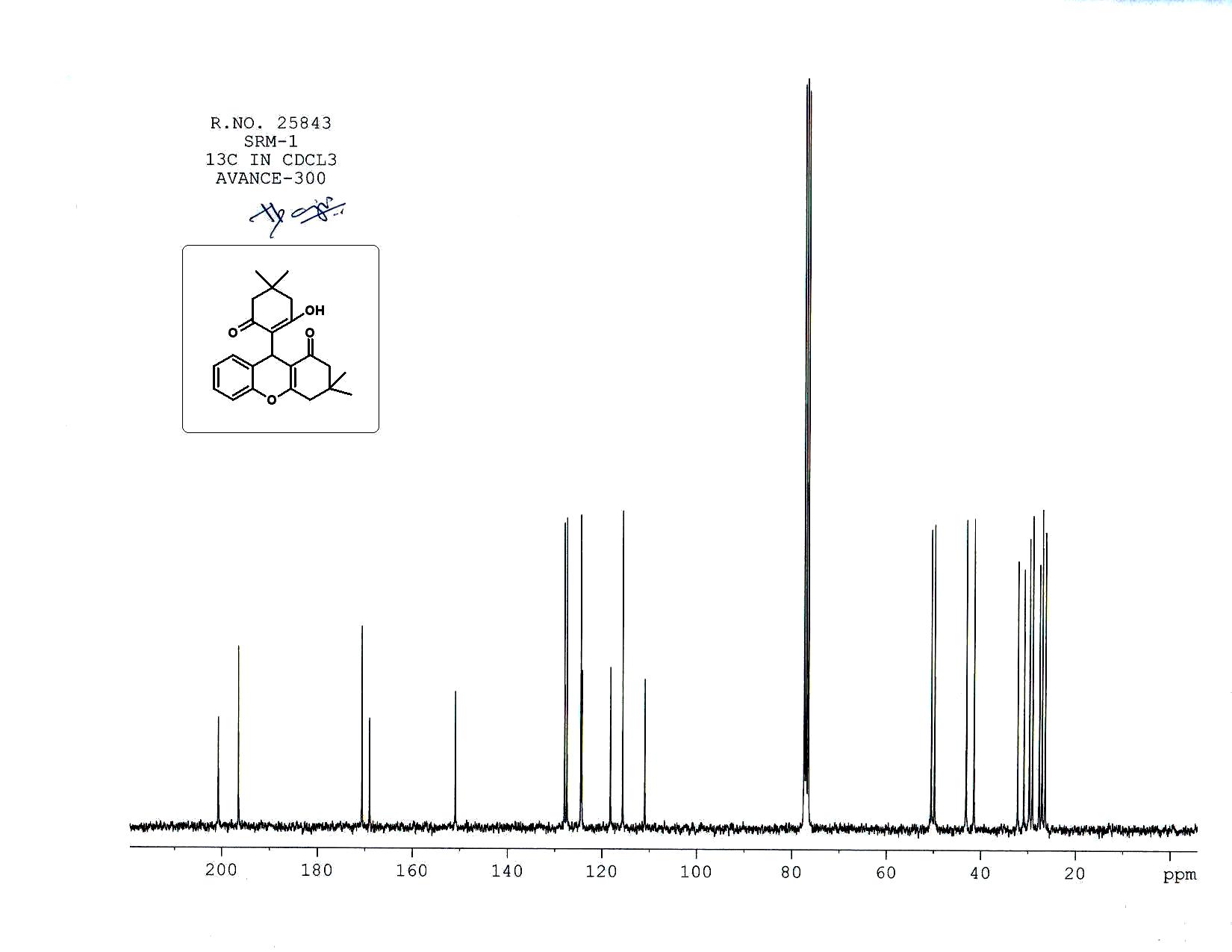 |
| Supplementary Figure S28 from ^13^CNMR spectrum of compound of 3a |
| **-S29-** |
| 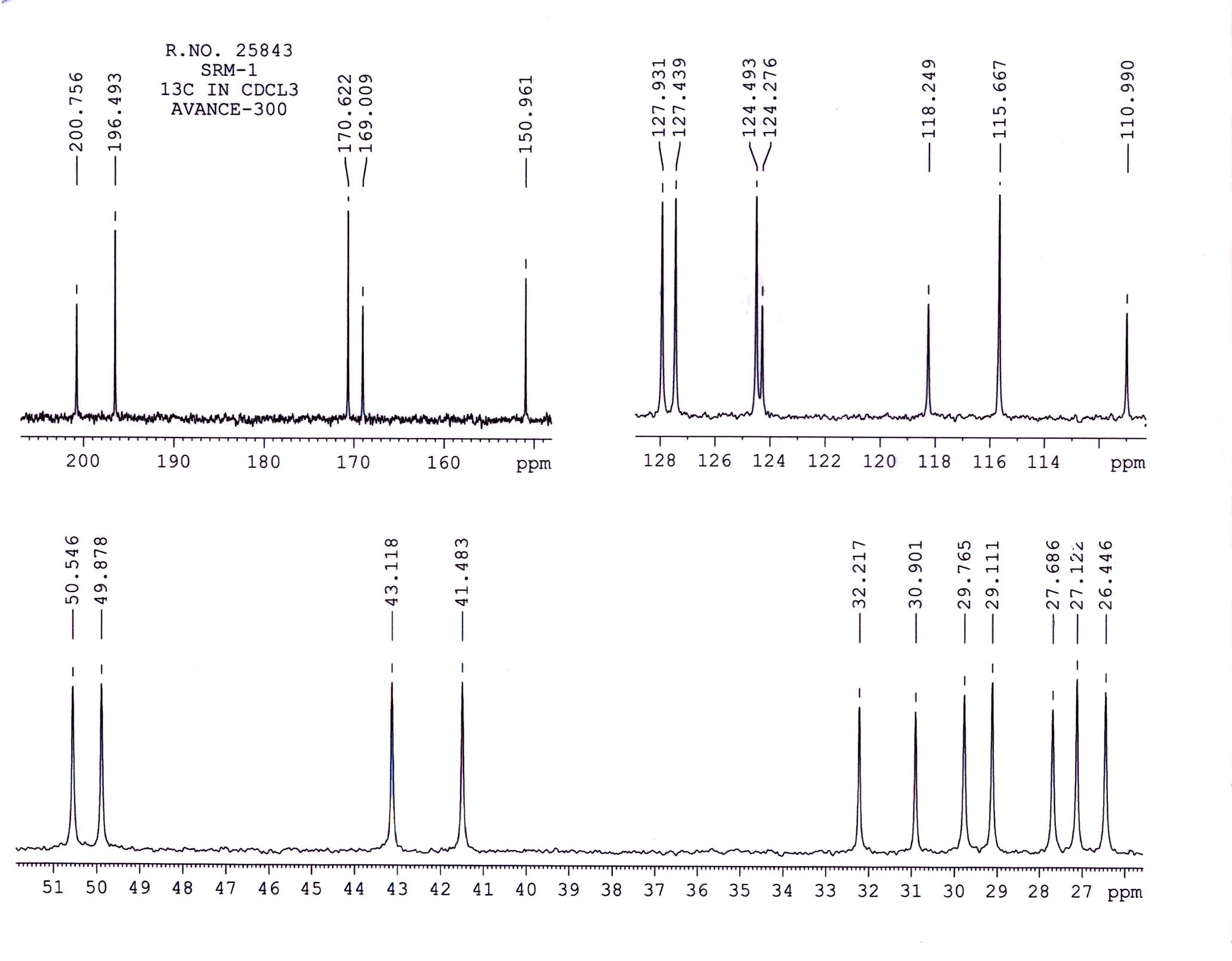 |
| Supplementary Figure S29 from Resolved ^13^C NMR spectrum of compound of 3a |
| **-S30-** |
| 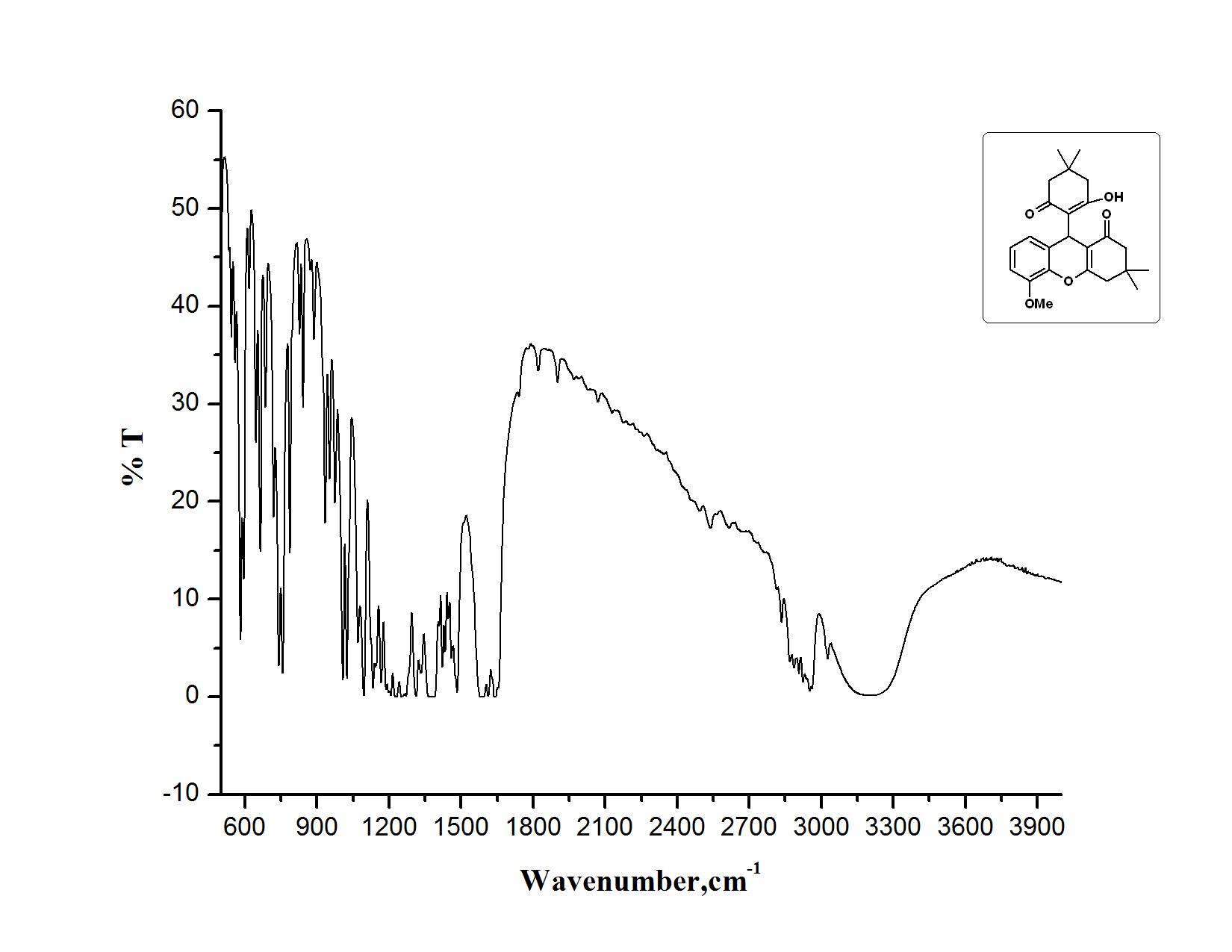 |
| Supplementary Figure S30 from FT-IR spectrum of 3b |
| **-S31-** |
| 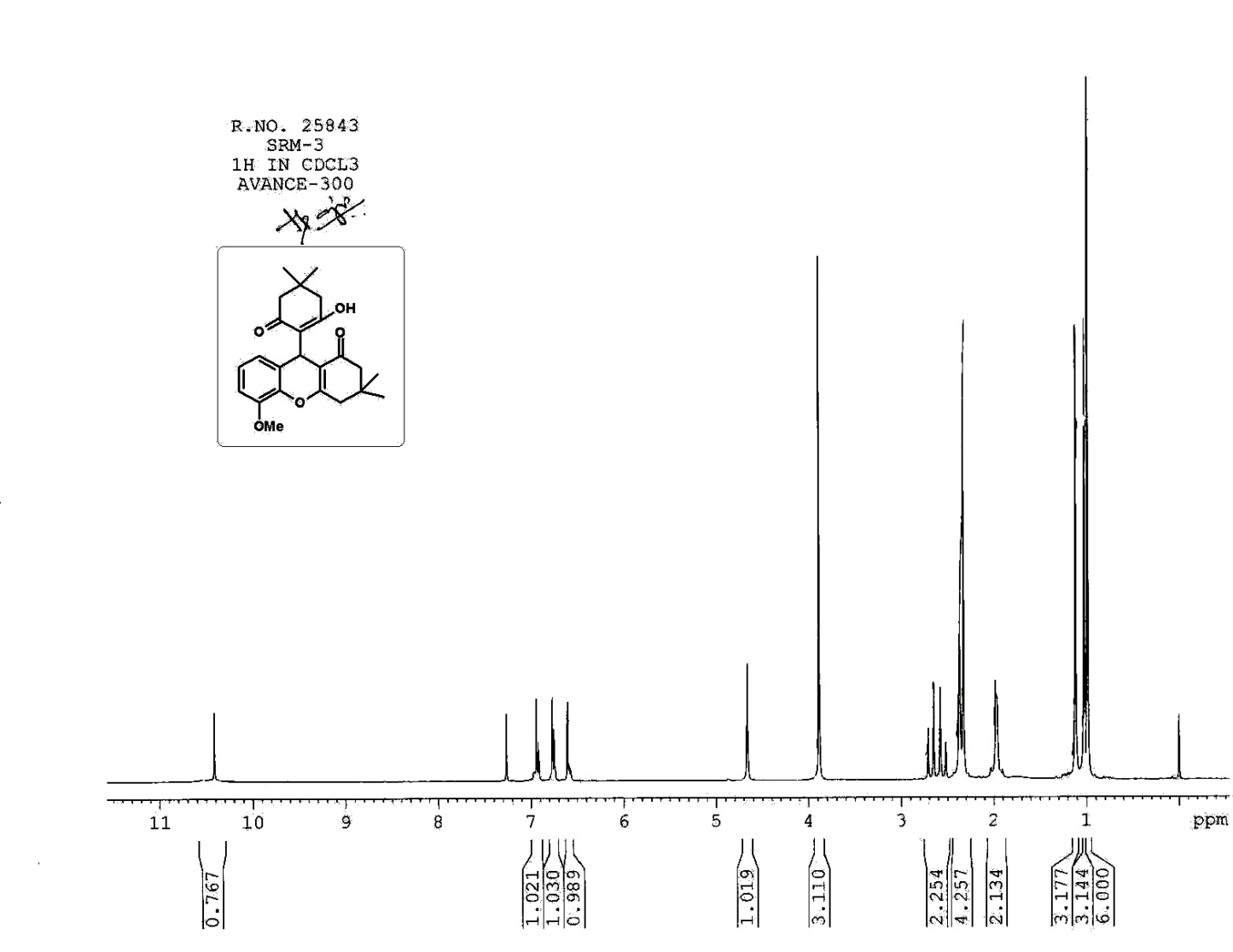 |
| Supplementary Figure S31 from ^1^HNMR spectrum of compound 3b |
| **-S32-** |
| 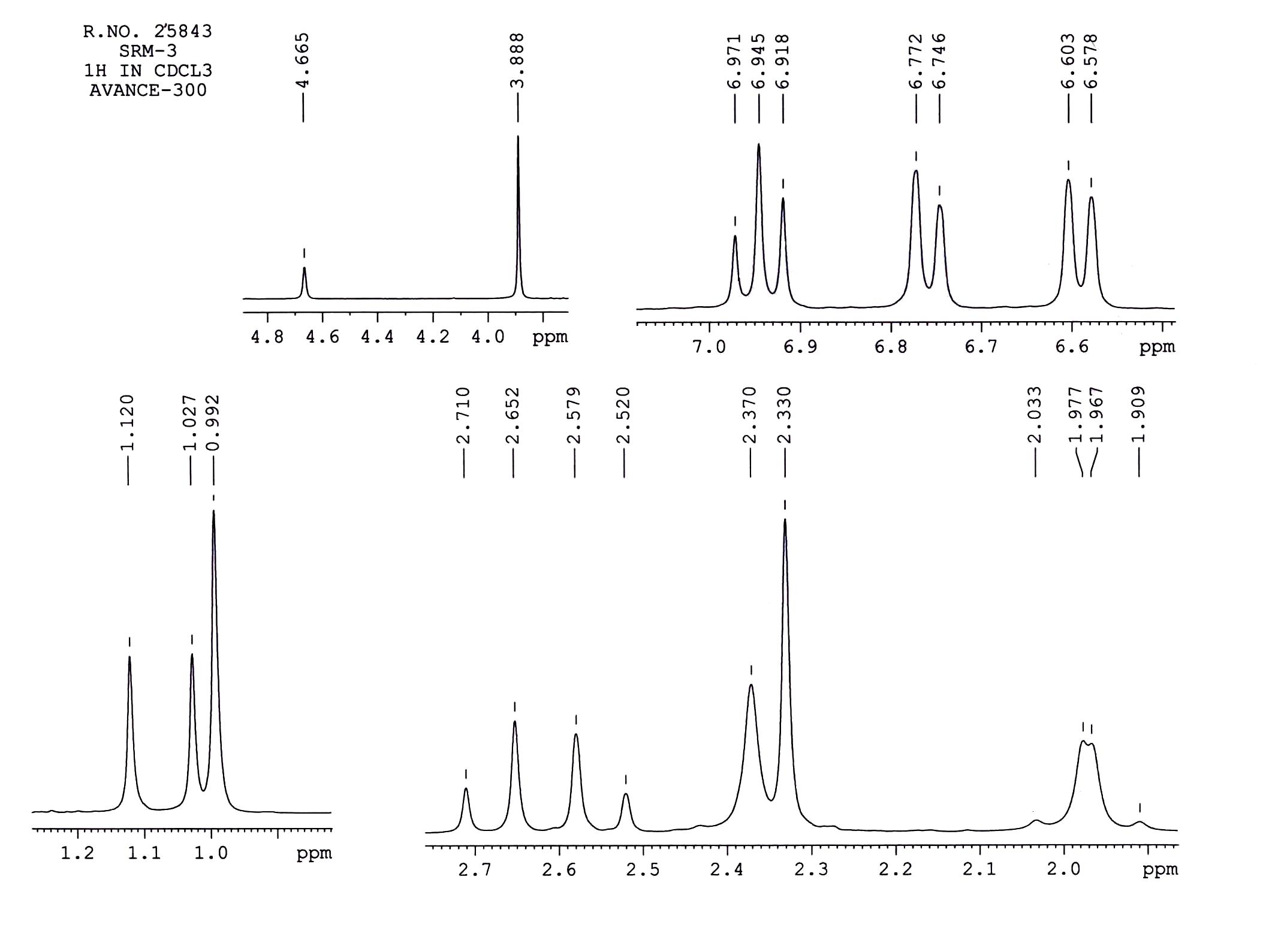 |
| Supplementary Figure S32 from Resolved^1^HNMR spectrum of compound of 3b |
| **-S33-** |
| 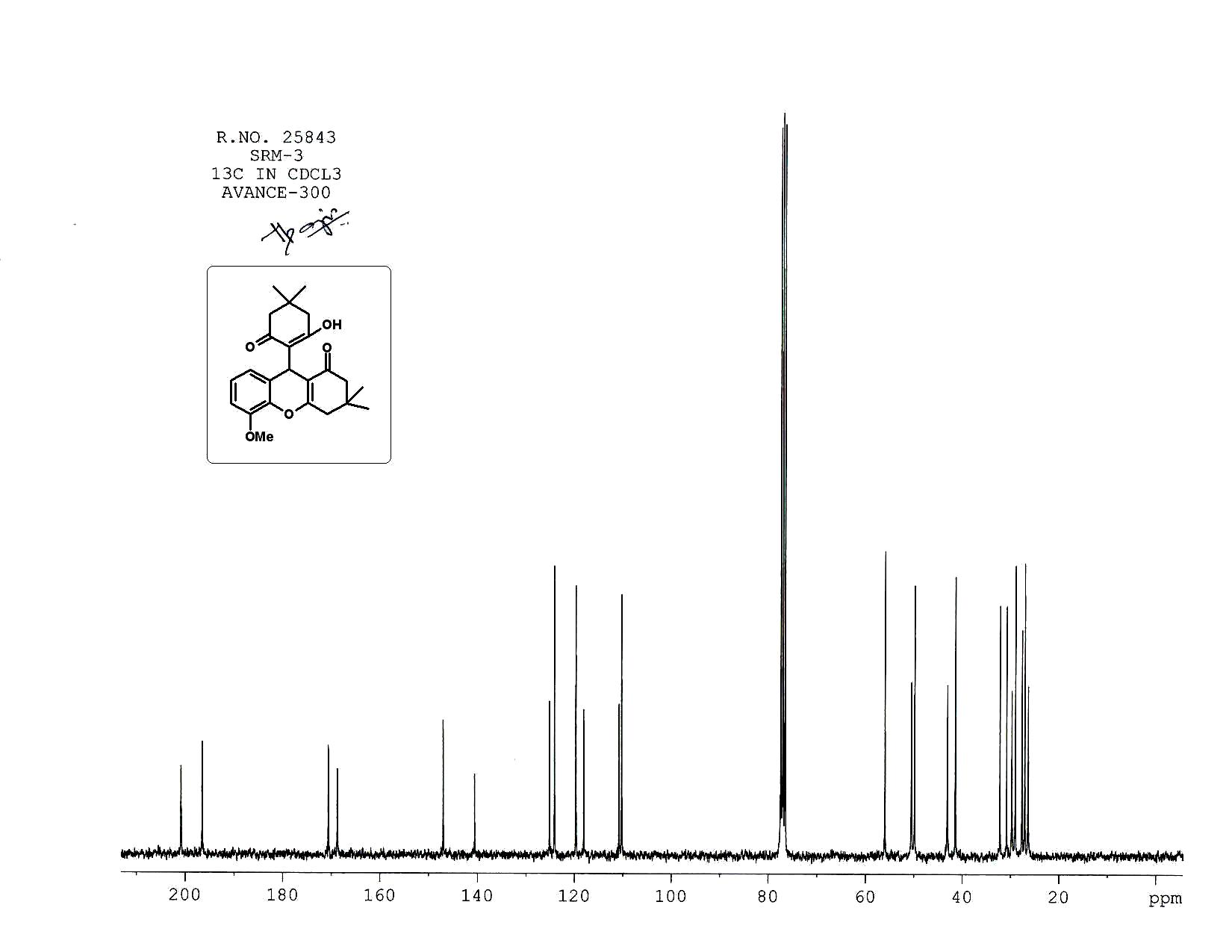 |
| Supplementary Figure S33 from ^13^CNMR spectrum of compound of 3b |
| **-S34-** |
| 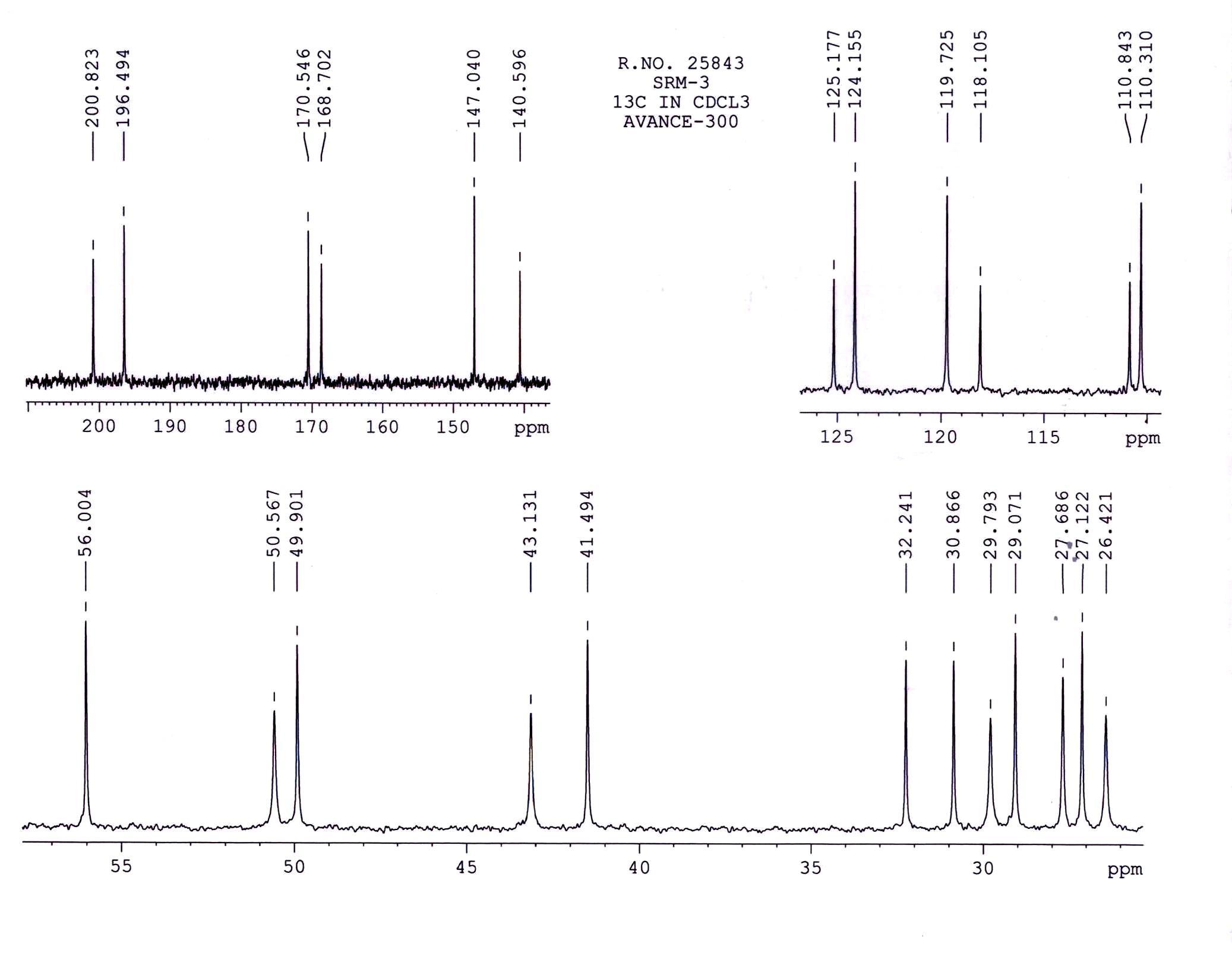 |
| Supplementary Figure S34 from Resolved ^13^C NMR spectrum of compound of 3b |
| **-S35-** |
| 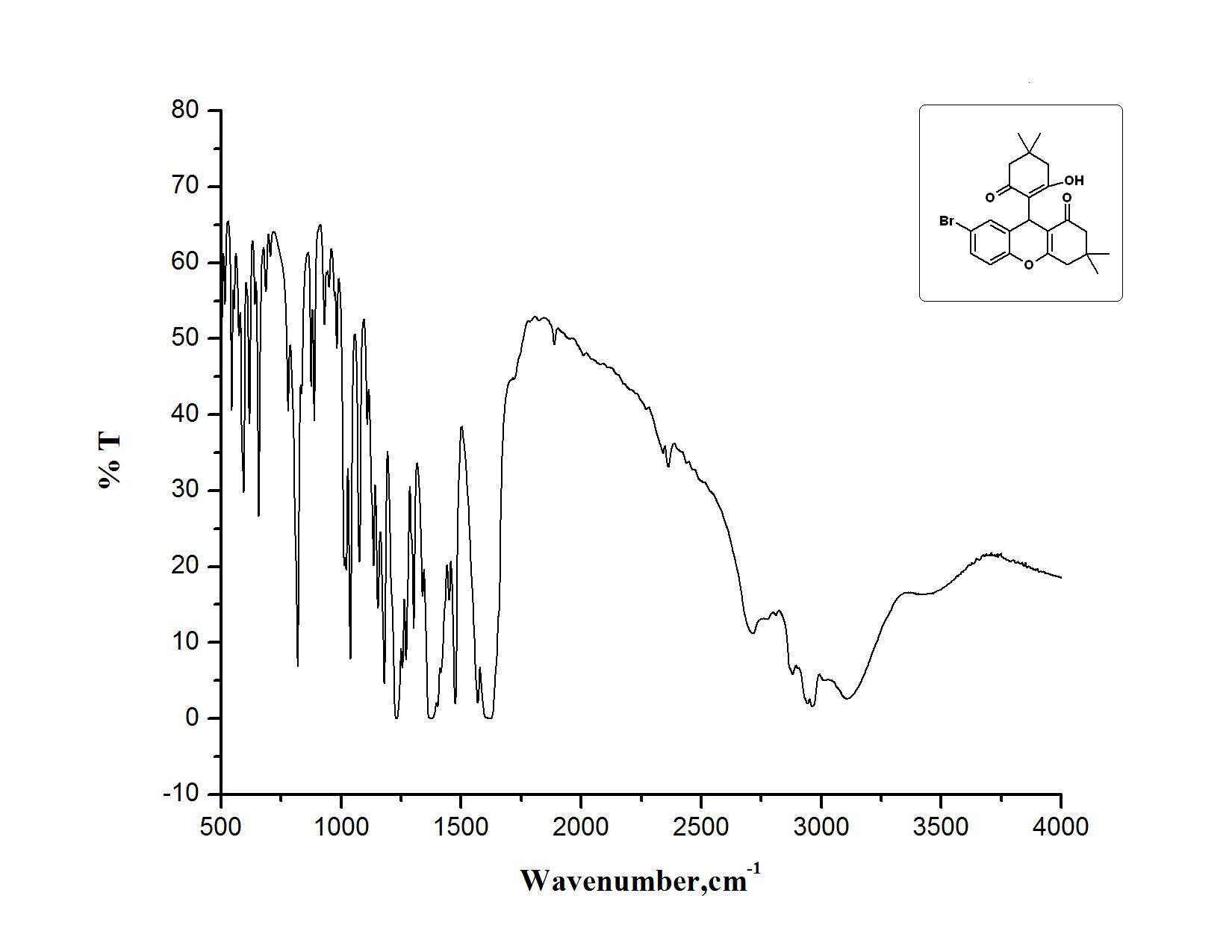 |
| Supplementary Figure S35 from FT-IR spectrum of compound 3c |
| **-S36-** |
| 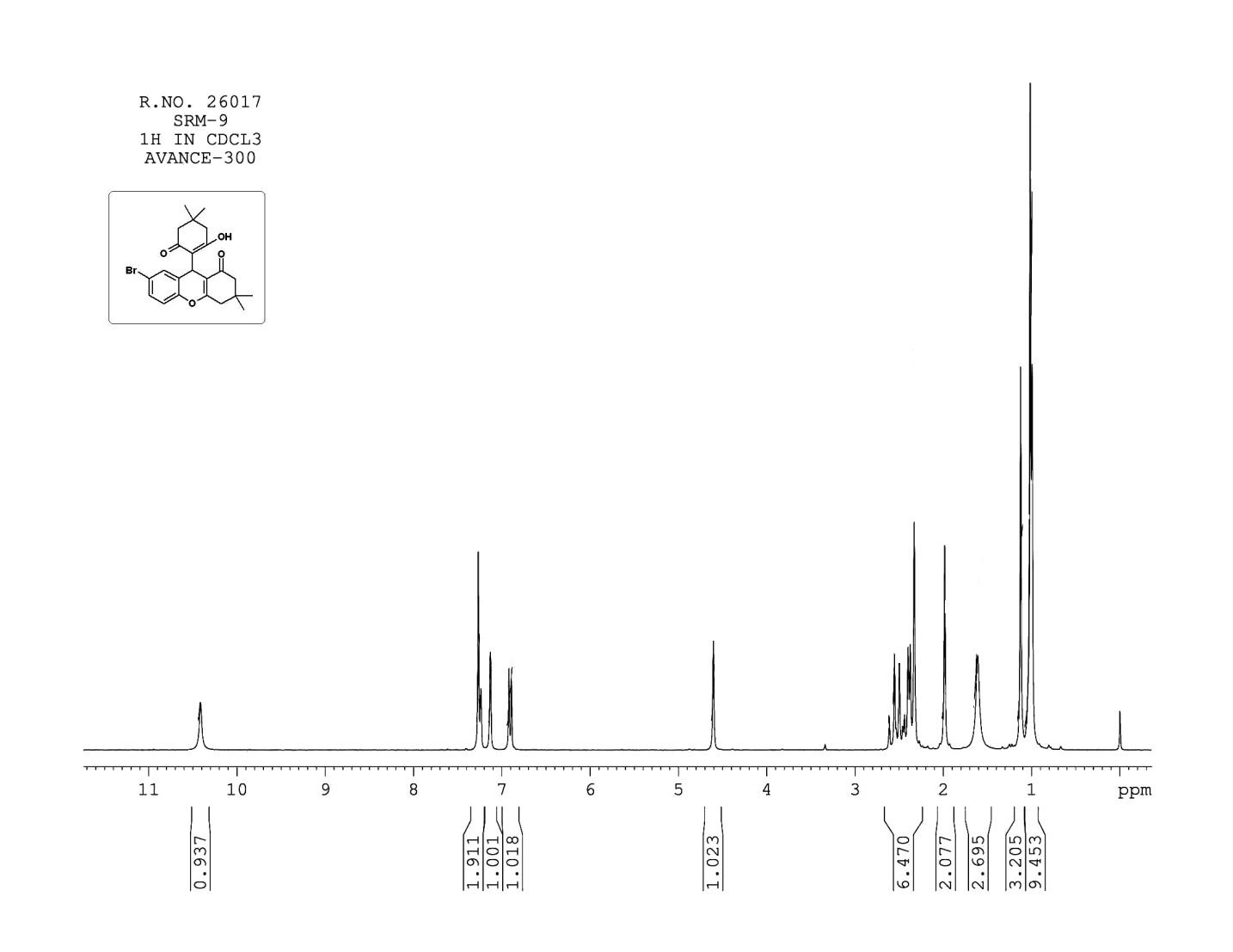 |
| Supplementary Figure S36 from ^1^HNMR spectrum of compound 3c |
| **-S37-** |
| 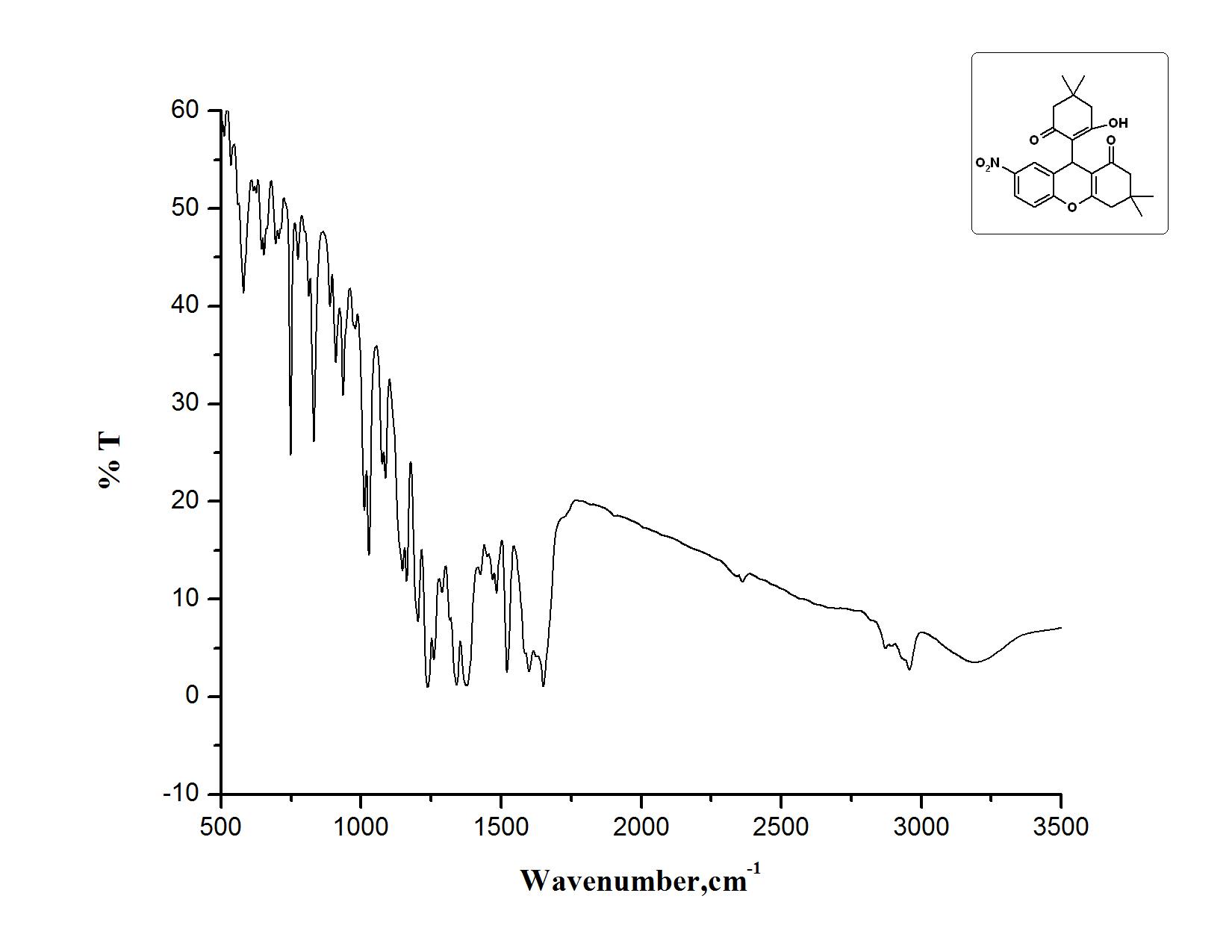 |
| Supplementary Figure S37 from FT-IR spectrum of 3d |
| **-S38-** |
| 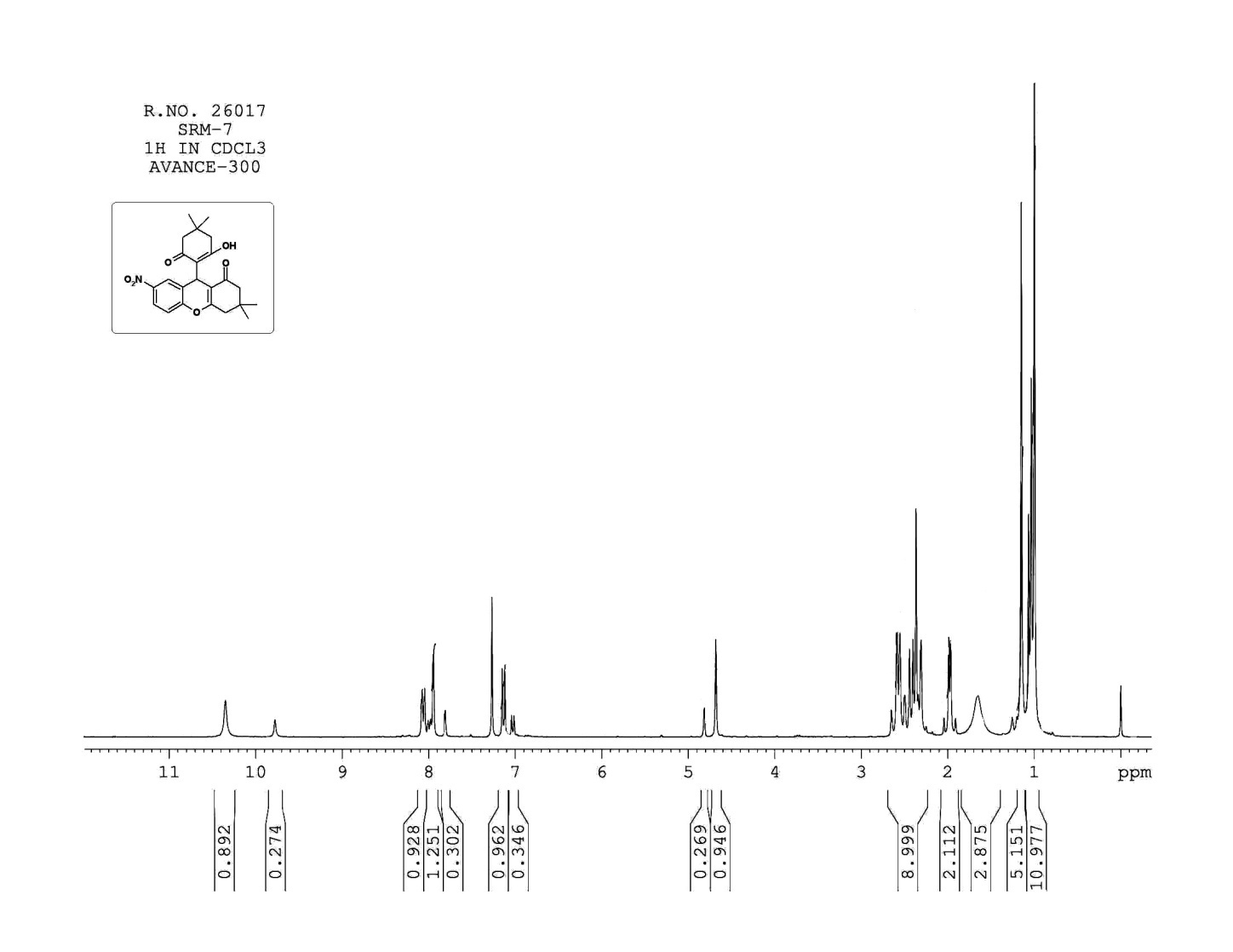 |
| Supplementary Figure S38 from ^1^HNMR spectrum of compound 3d |
| **-S39-** |
| 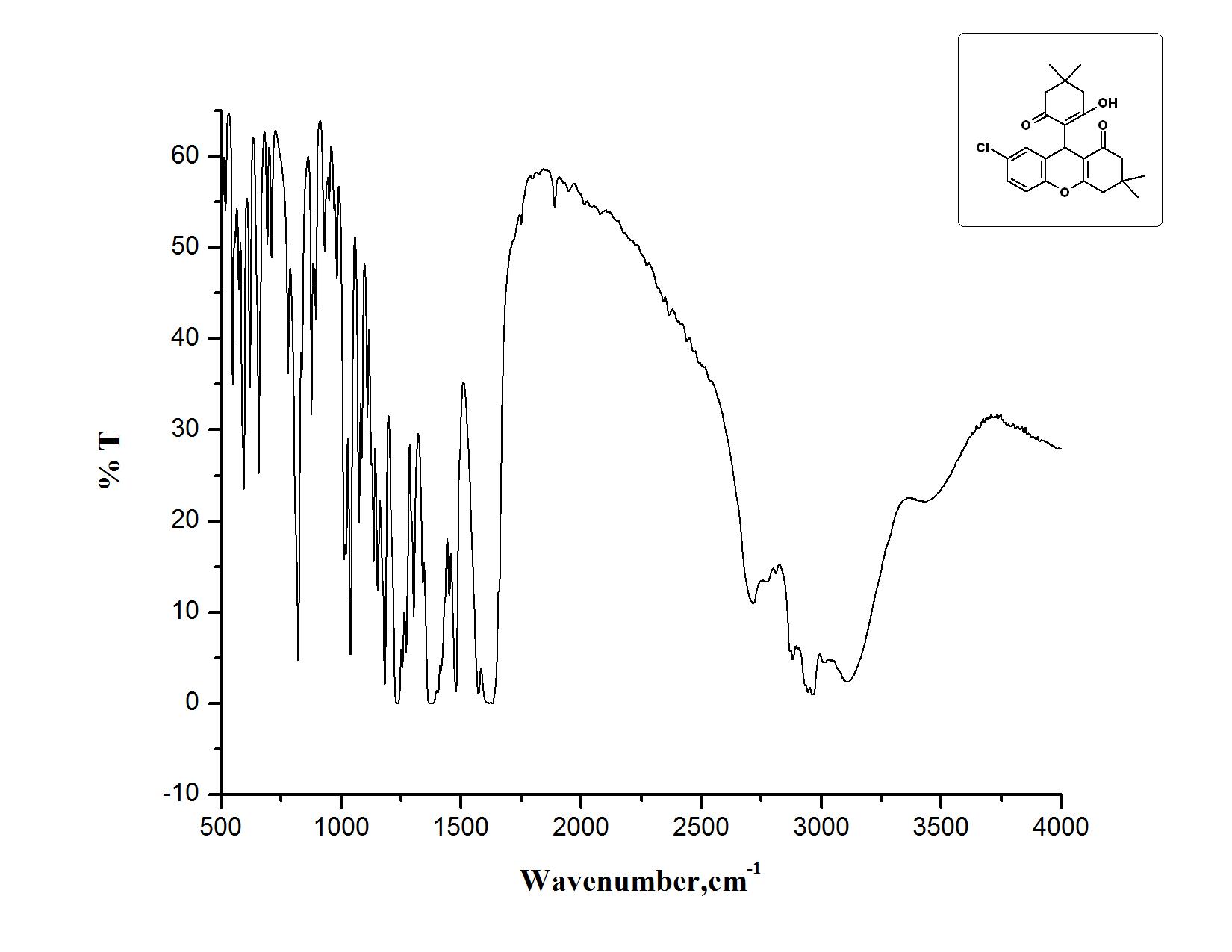 |
| Supplementary Figure S39 from FT-IR spectrum of compound 3e |
| **-S40-** |
| 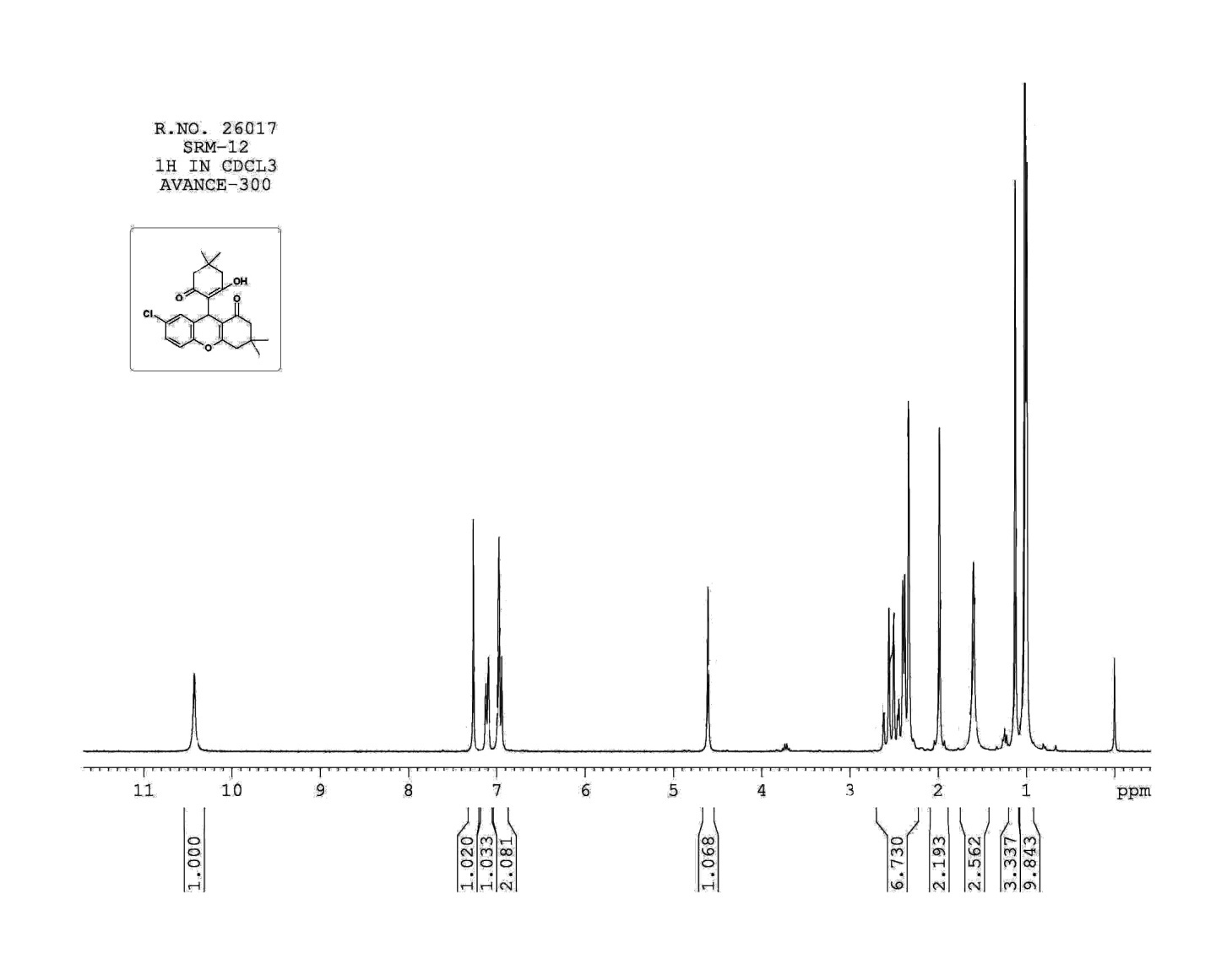 |
| Supplementary Figure S40 from ^1^HNMR spectrum of compound 3e |
| **-S41-** |
| 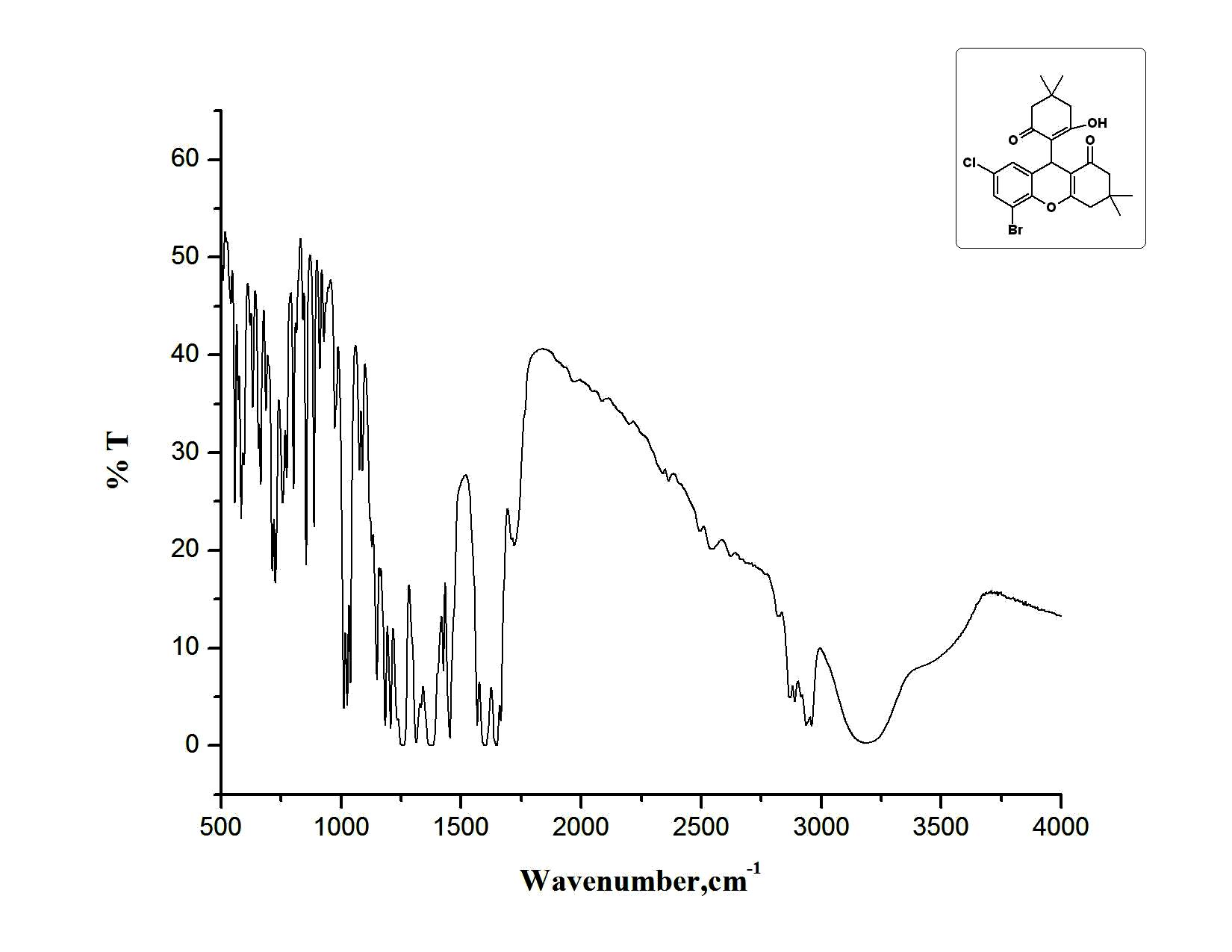 |
| Supplementary Figure S41 from FT-IR spectrum of compound 3f |
| **-S42-** |
| 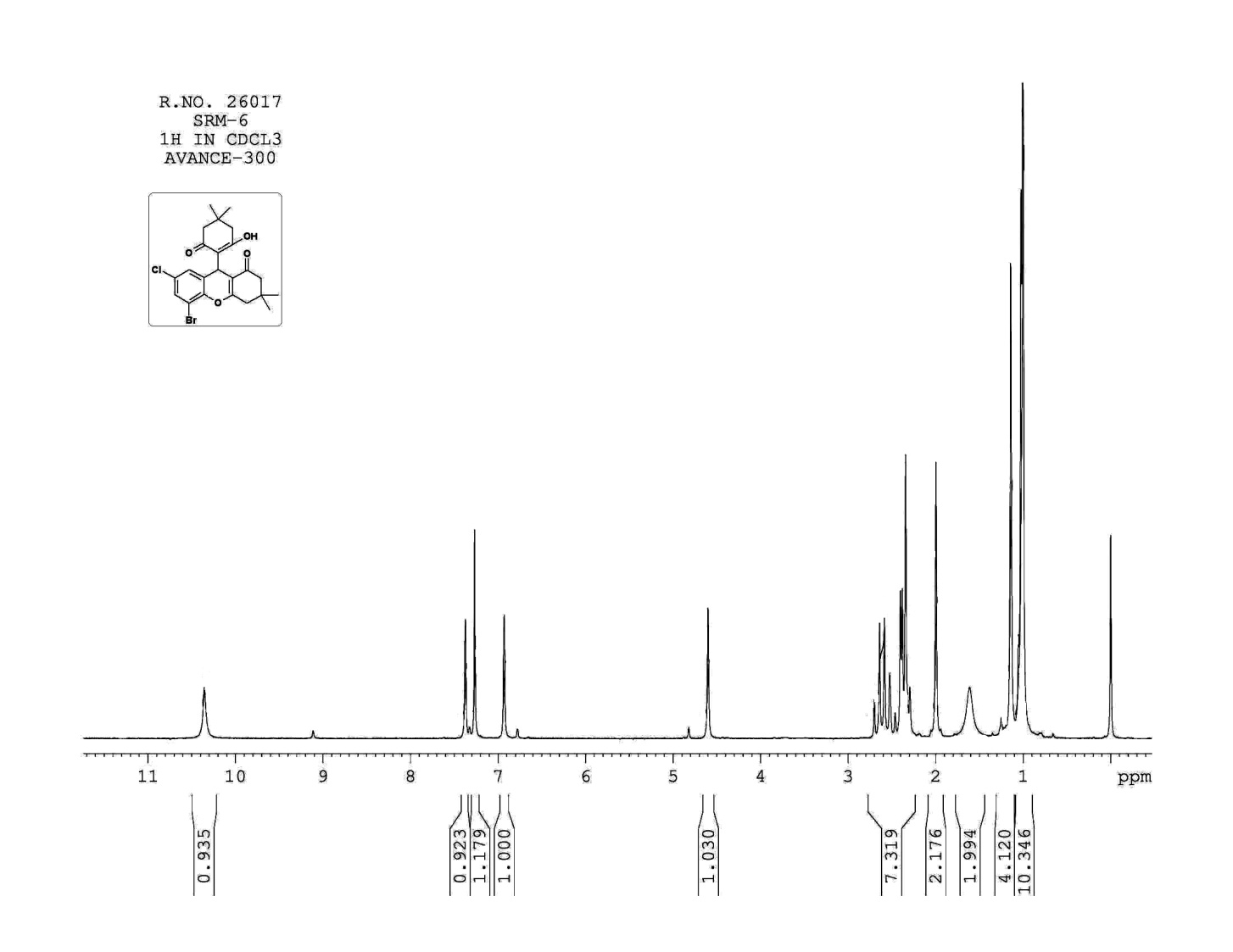 |
| Supplementary Figure S42 from ^1^HNMR spectrum of compound 3f |
| **-S43-** |
| 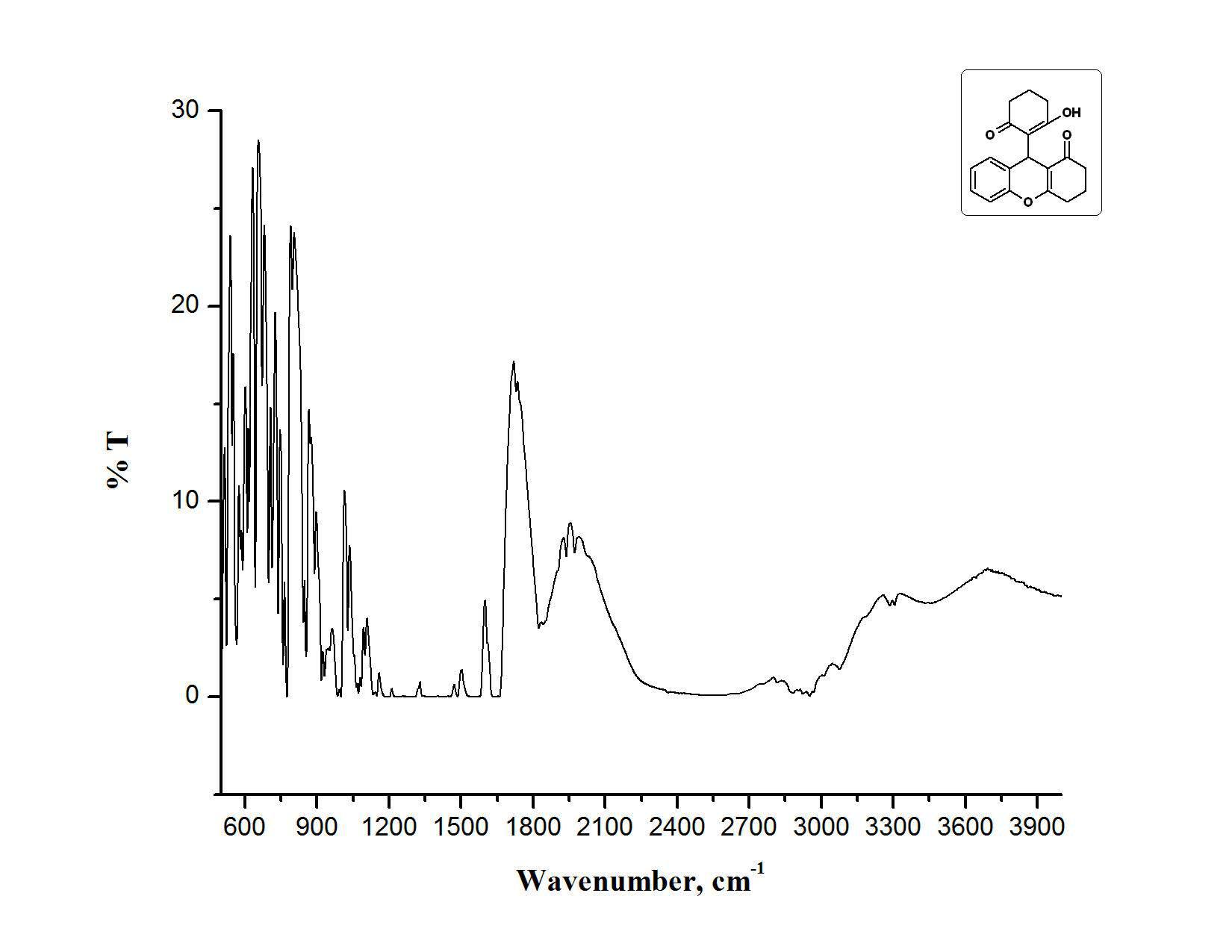 |
| Supplementary Figure S43 from FT-IR spectrum of compound 3g |
| **-S44-** |
| 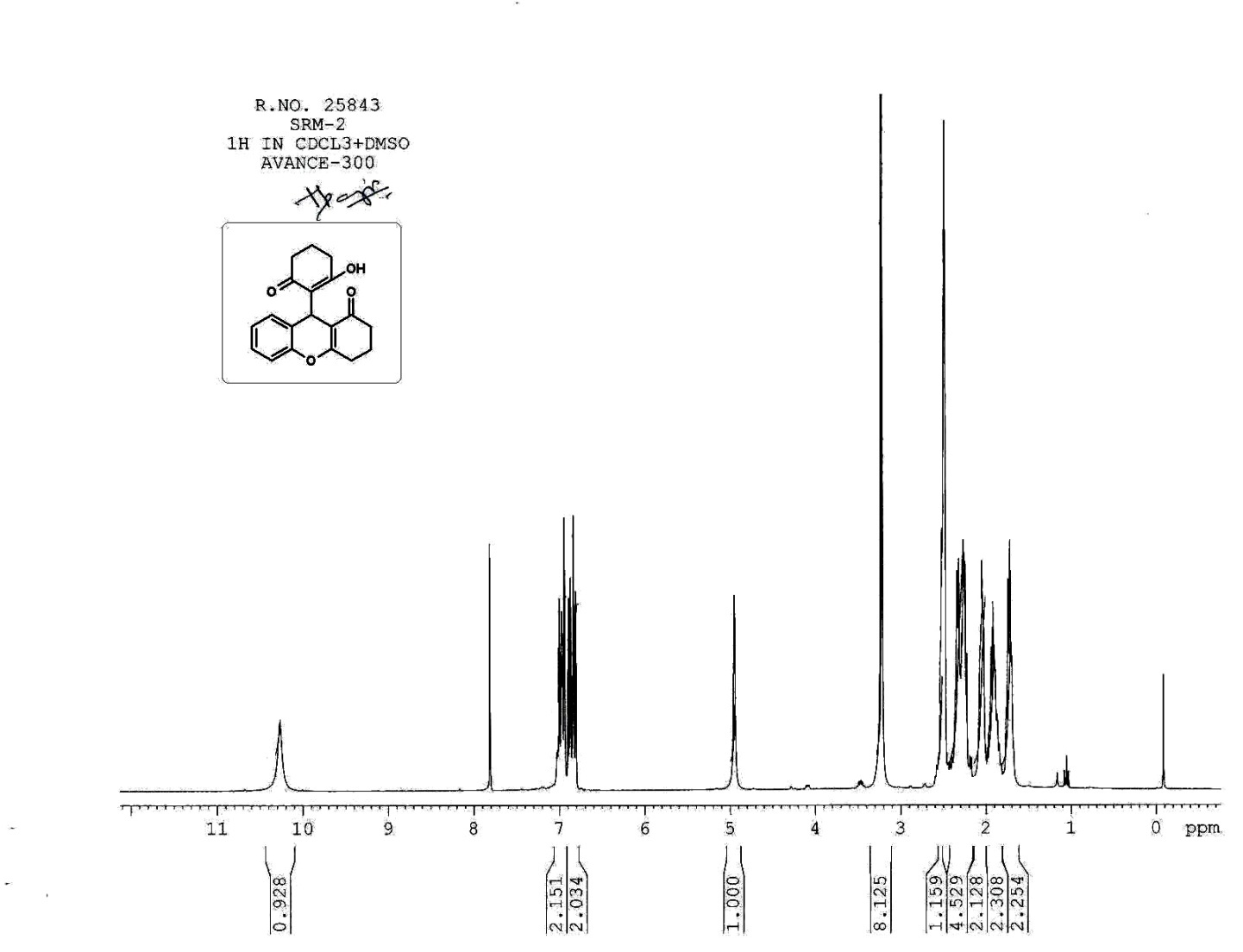 |
| Supplementary Figure S44 from ^1^HNMR spectrum of compound 3g |
| **-S45-** |
| 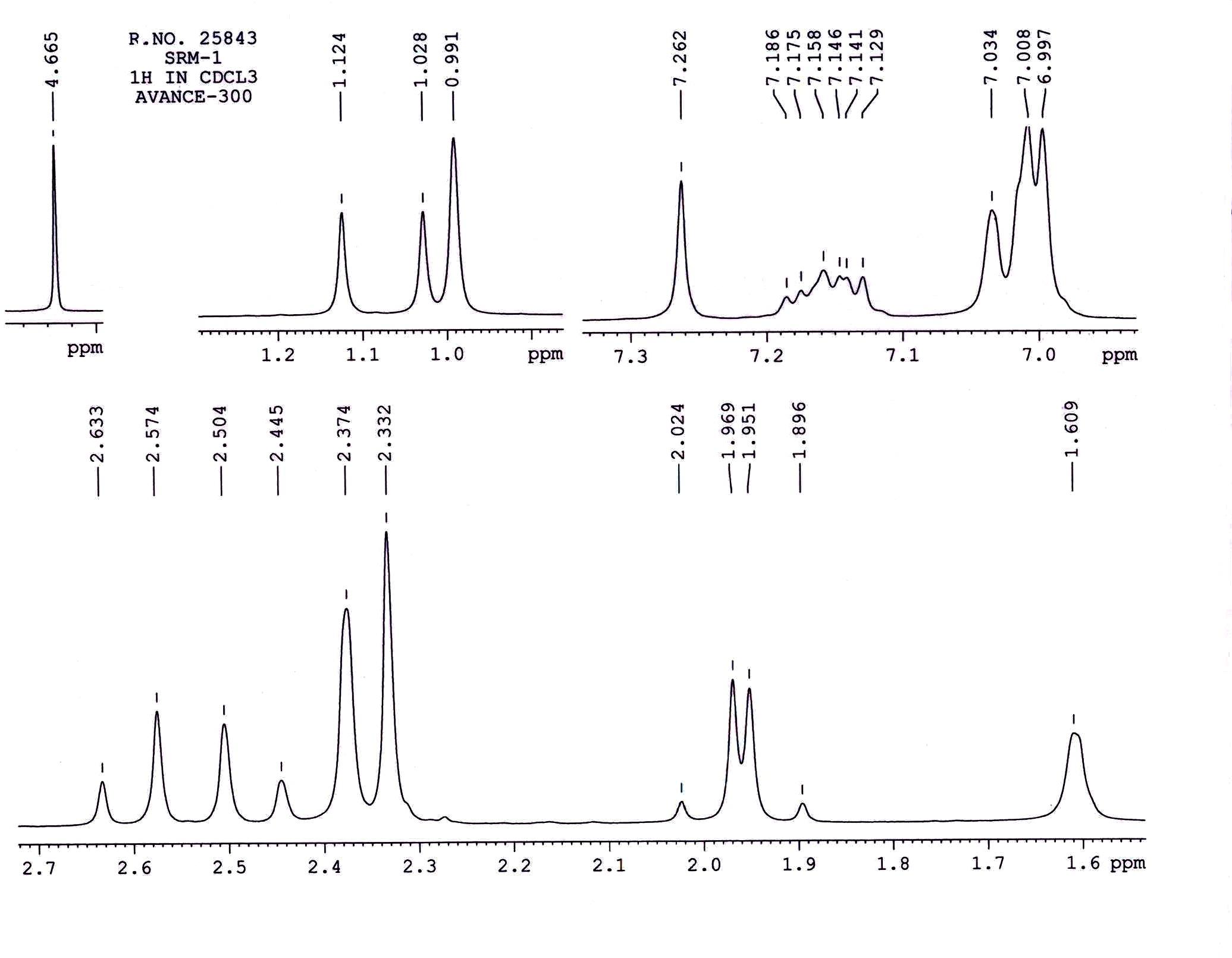 |
| Supplementary Figure S45 from Resolved^1^HNMR spectrum of compound of 3g |
| **-S46-** |
| 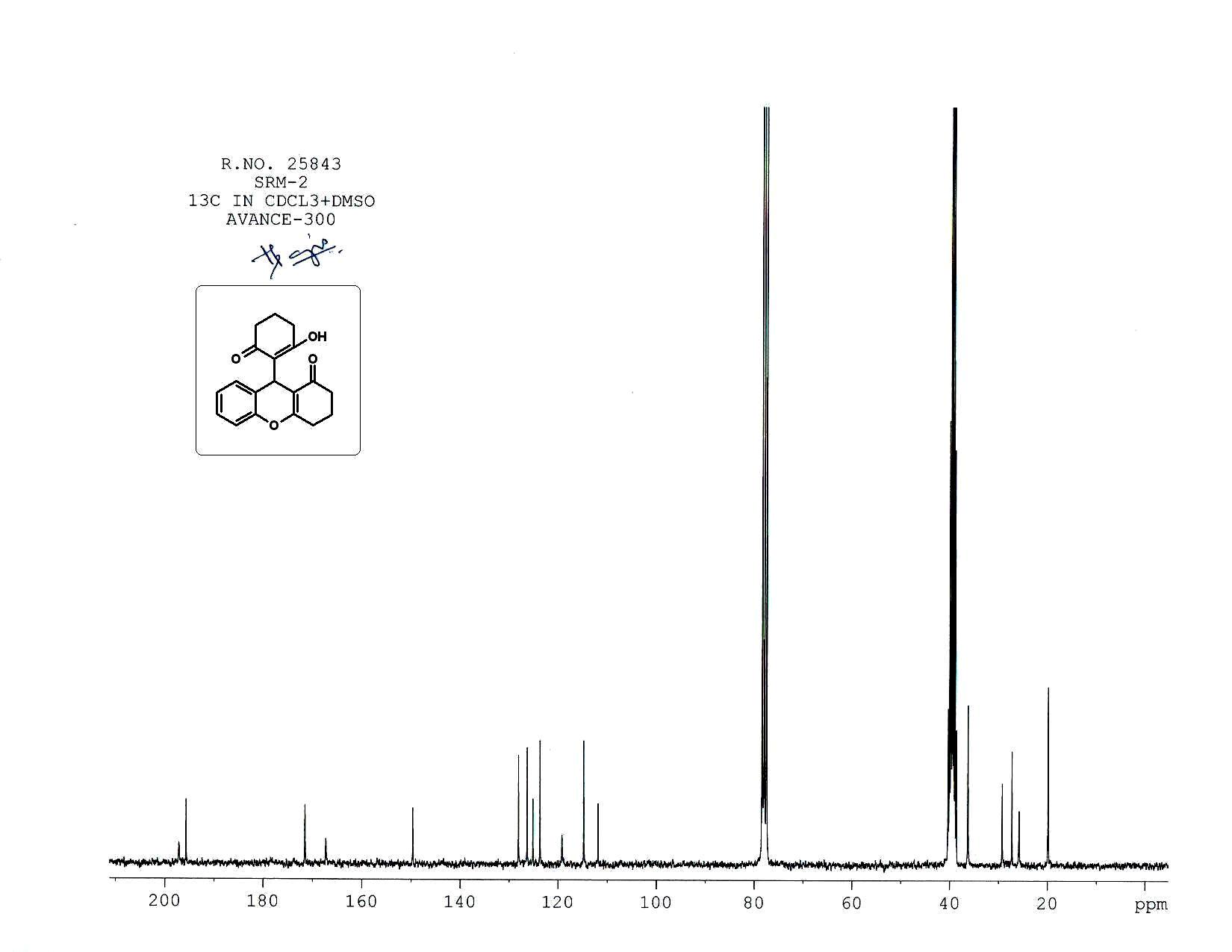 |
| Supplementary Figure S46 from ^13^CNMR spectrum of compound of 3g |
| **-S47-** |
| 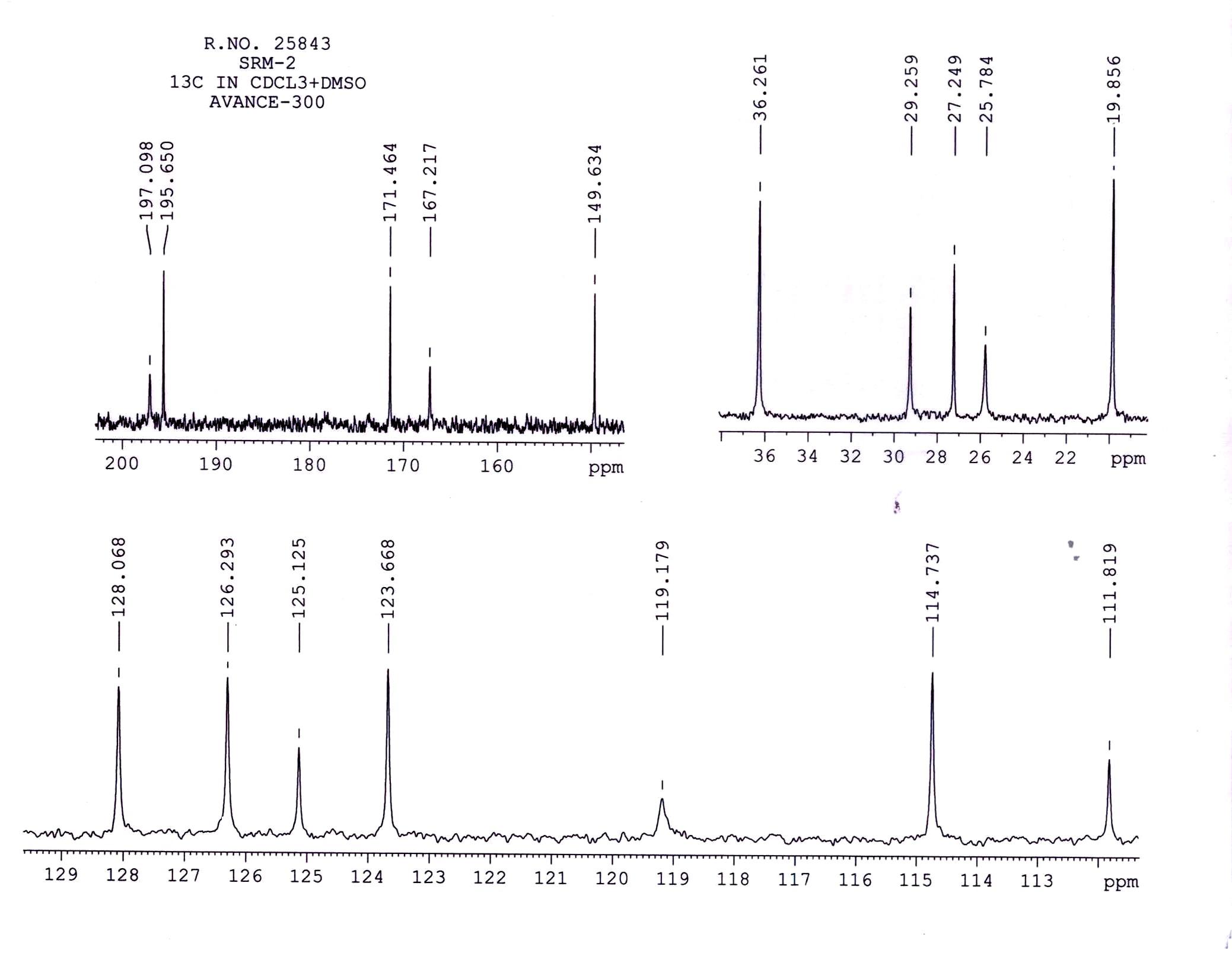 |
| Supplementary Figure S47 from Resolved ^13^C NMR spectrum of compound of 3g |
| **-S48-** |
| 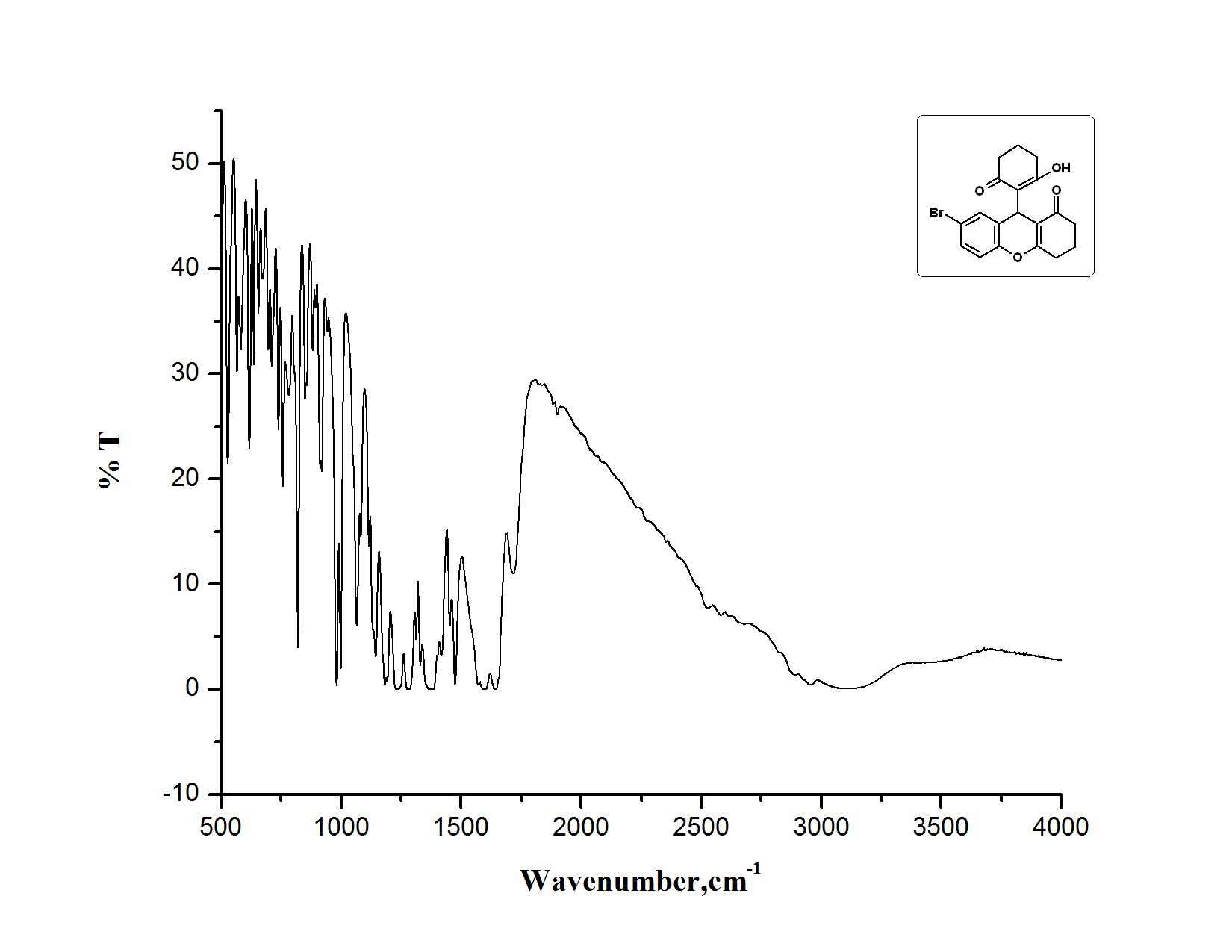 |
| Supplementary Figure S48 from FT-IR spectrum of 3i |
| **-S49-** |
| 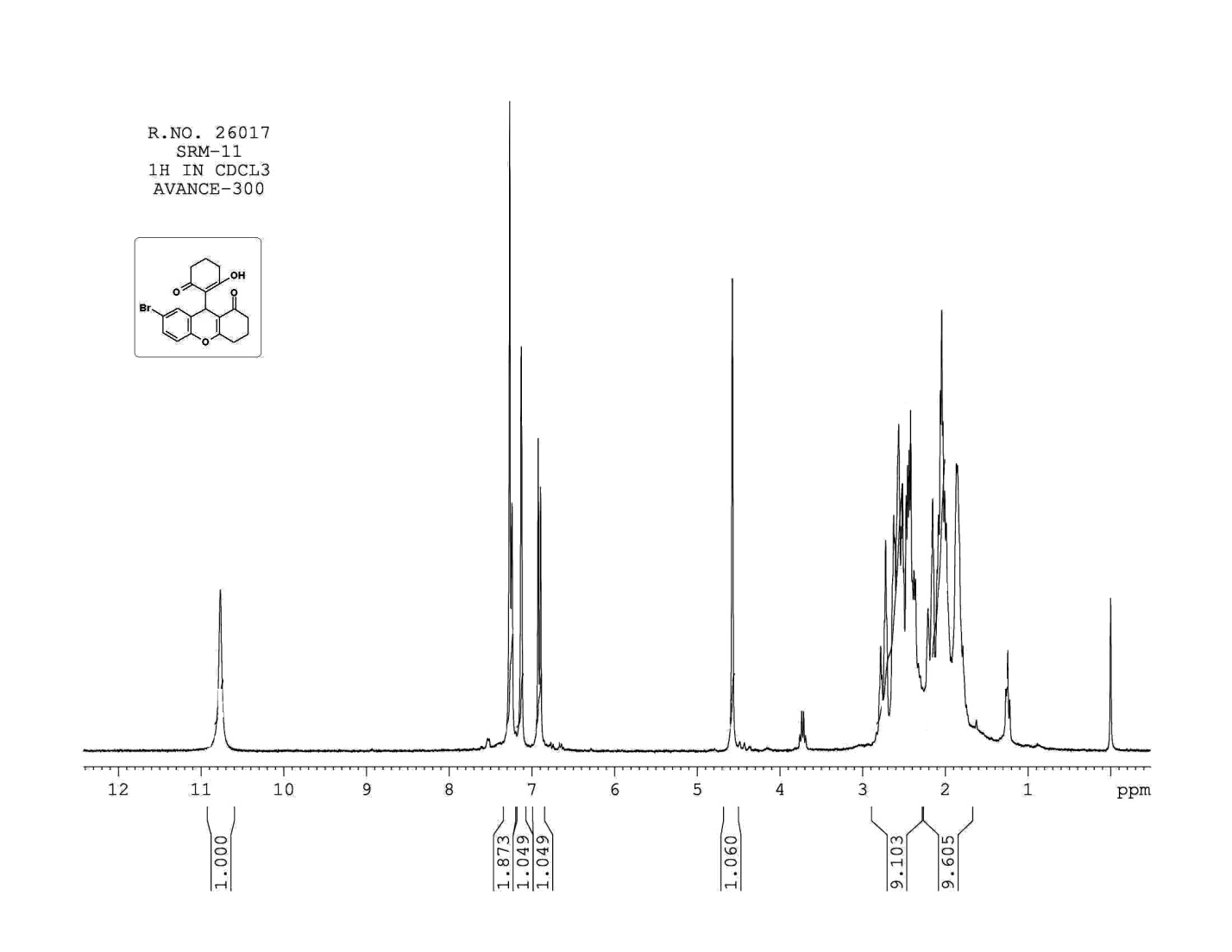 |
| Supplementary Figure S49 from ^1^HNMR spectrum of compound 3i |
| **-S50-** |
| 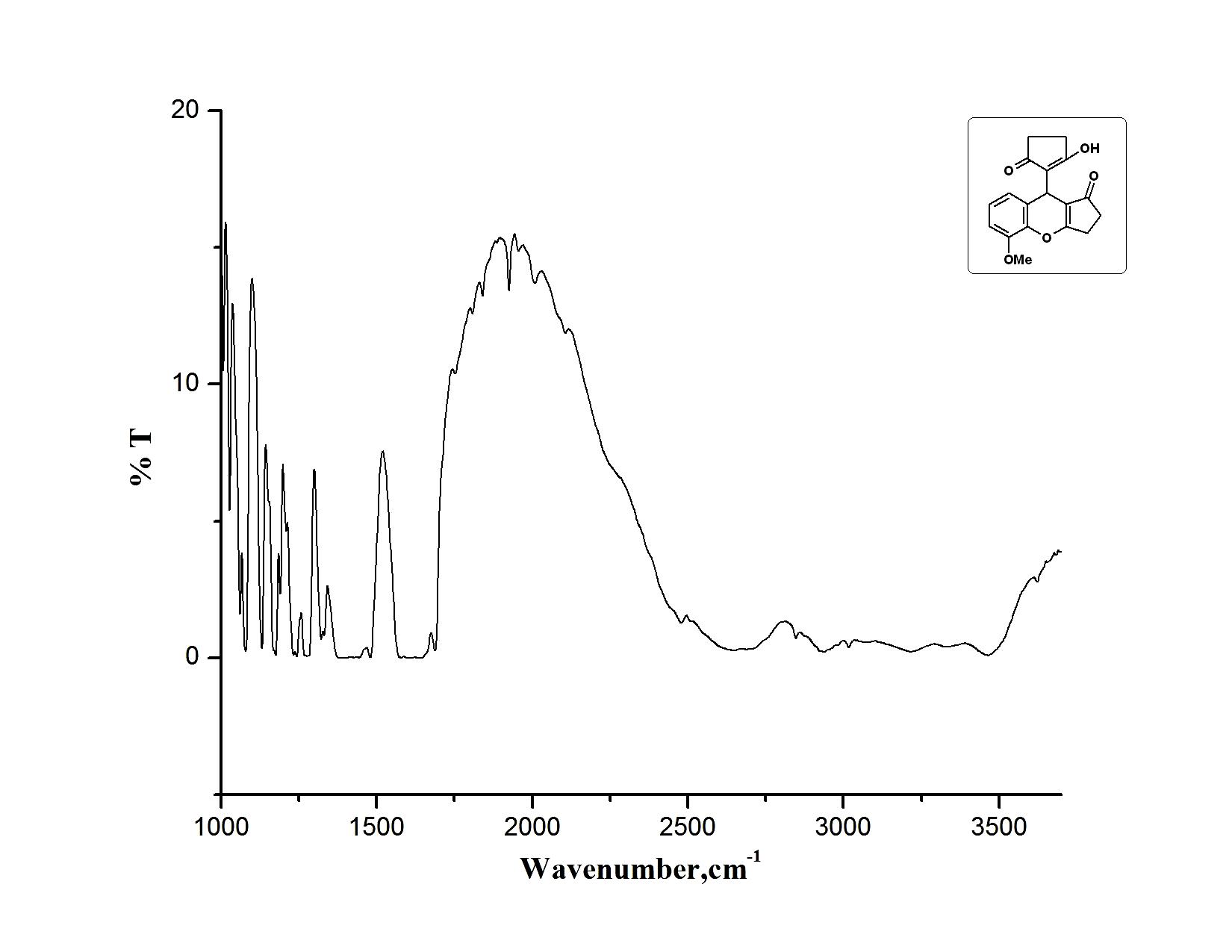 |
| Supplementary Figure S50 from FT-IR spectrum of compound 3o |
| **-S51-** |
| 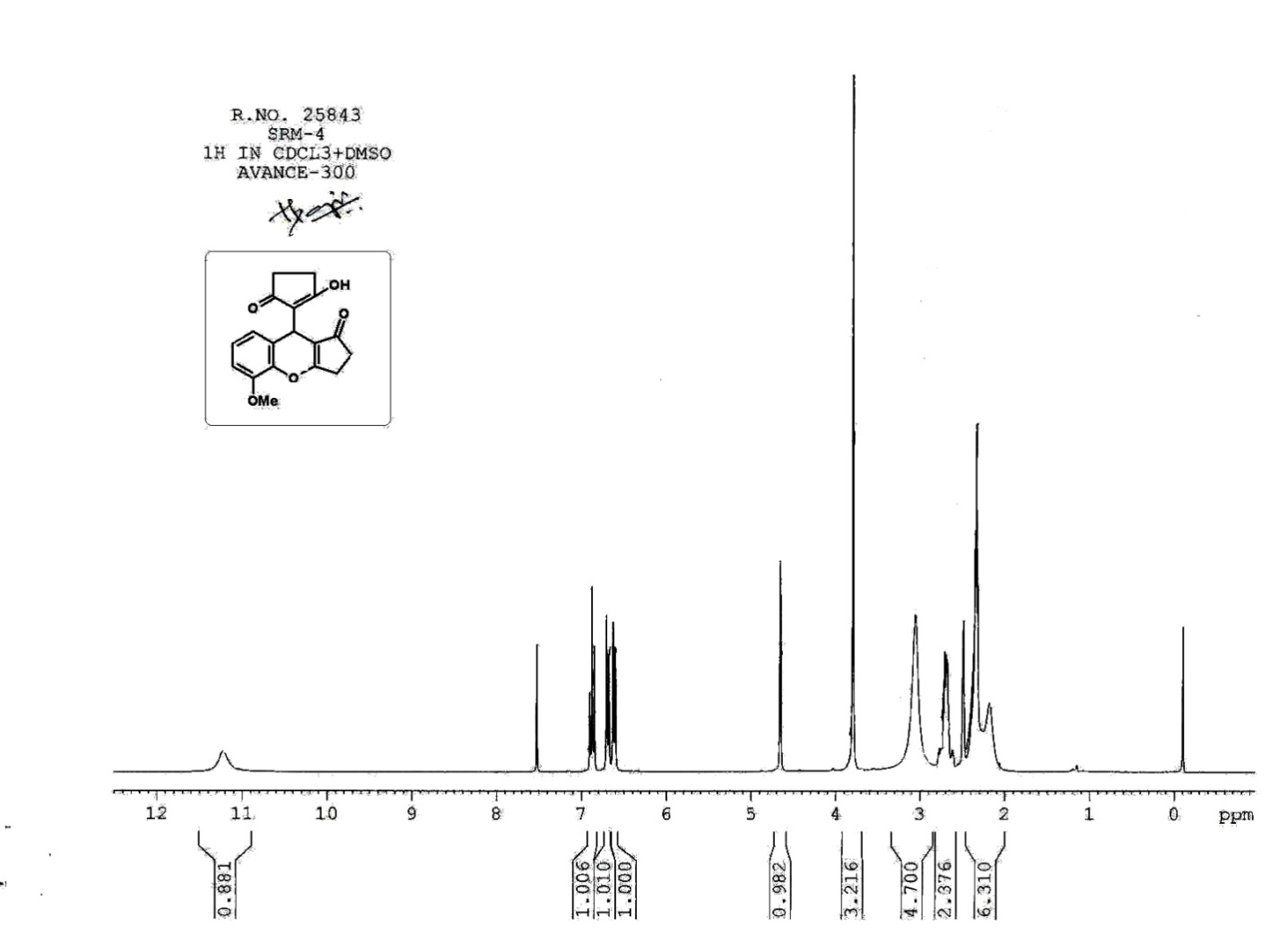 |
| Supplementary Figure S51 from ^1^HNMR spectrum of compound 3o |
| **-S52-** |
| 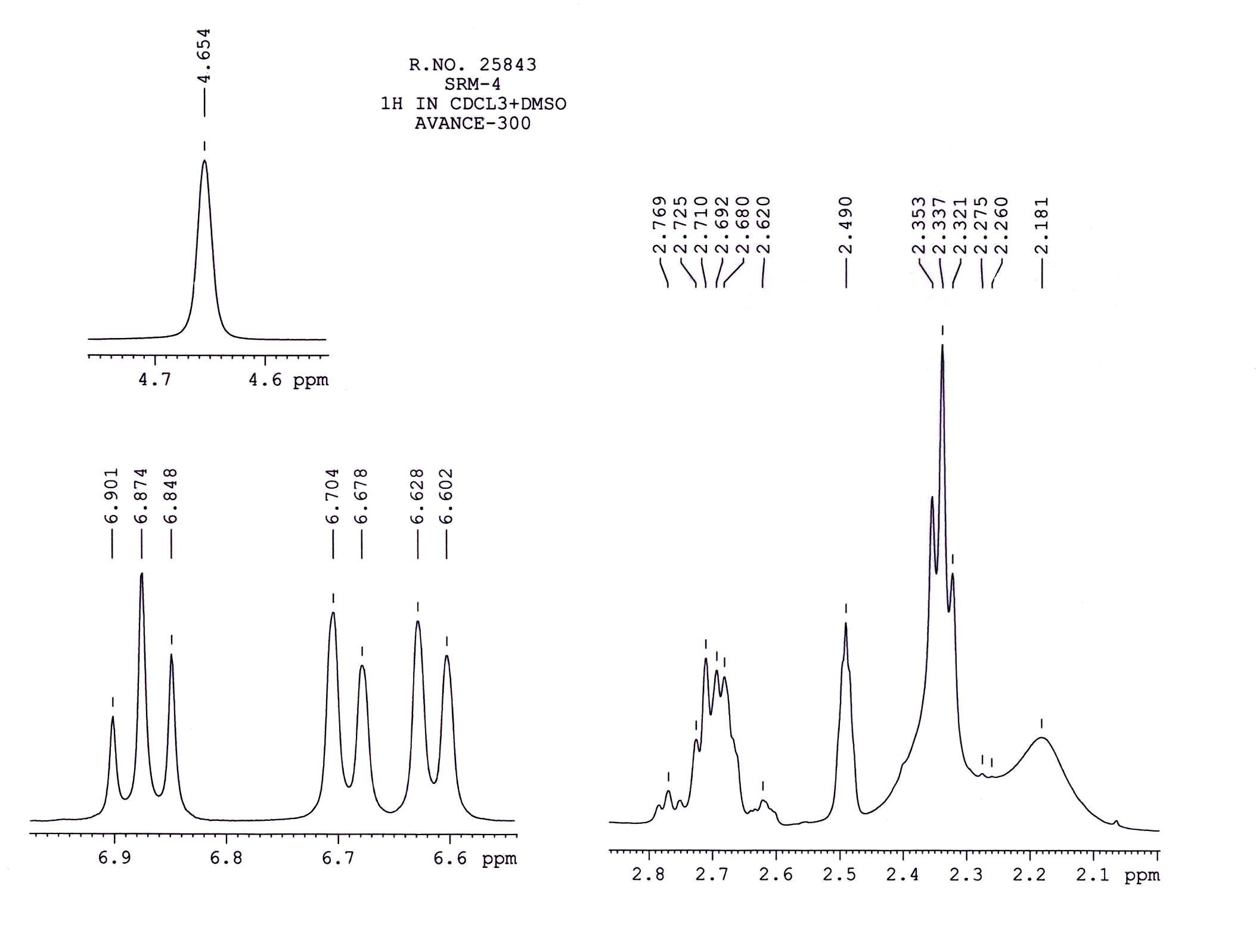 |
| Supplementary Figure S52 from Resolved^1^HNMR spectrum of compound 3o |
| **-S53-** |
| 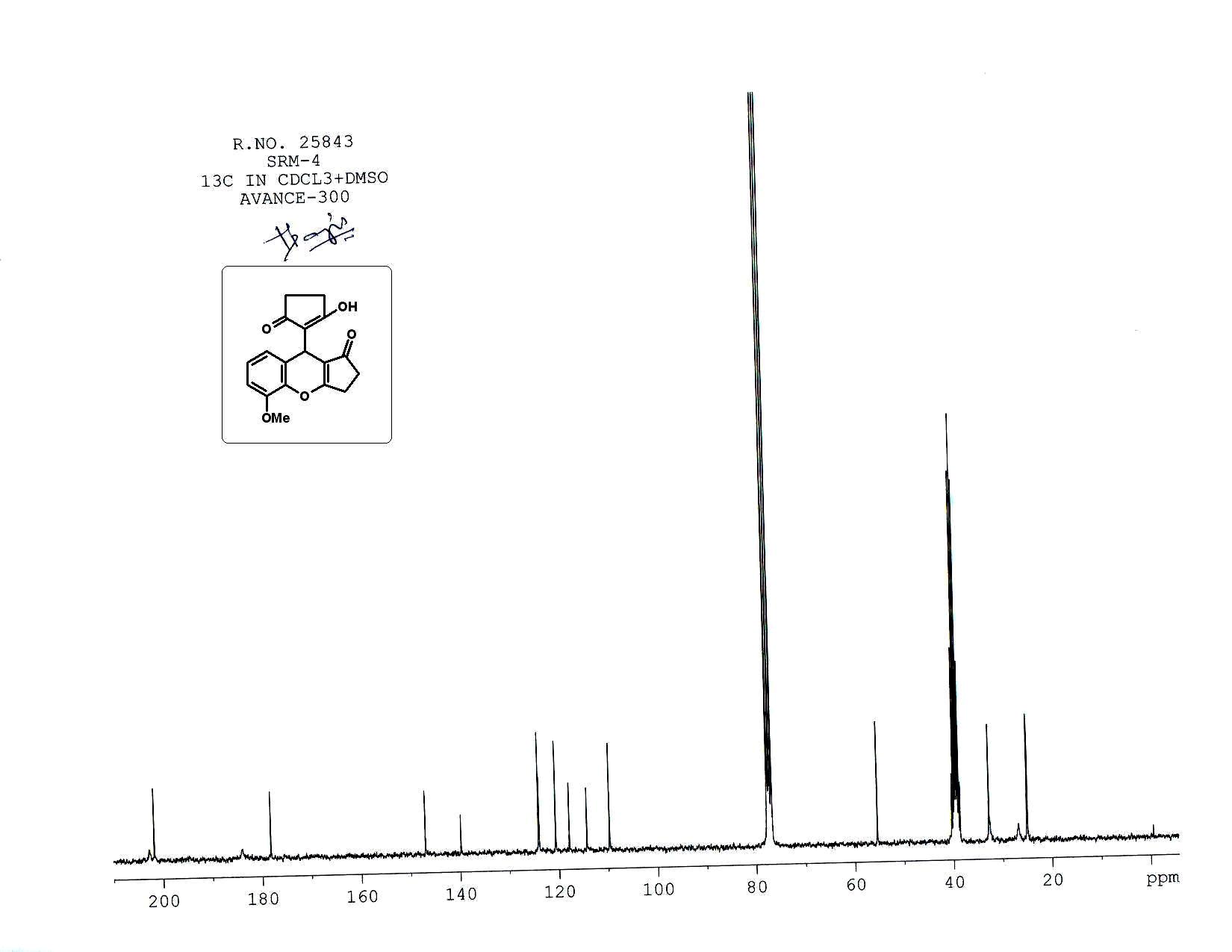 |
| Supplementary Figure S53 from ^13^CNMR spectrum of compound of 3o |
| **-S54-** |
| 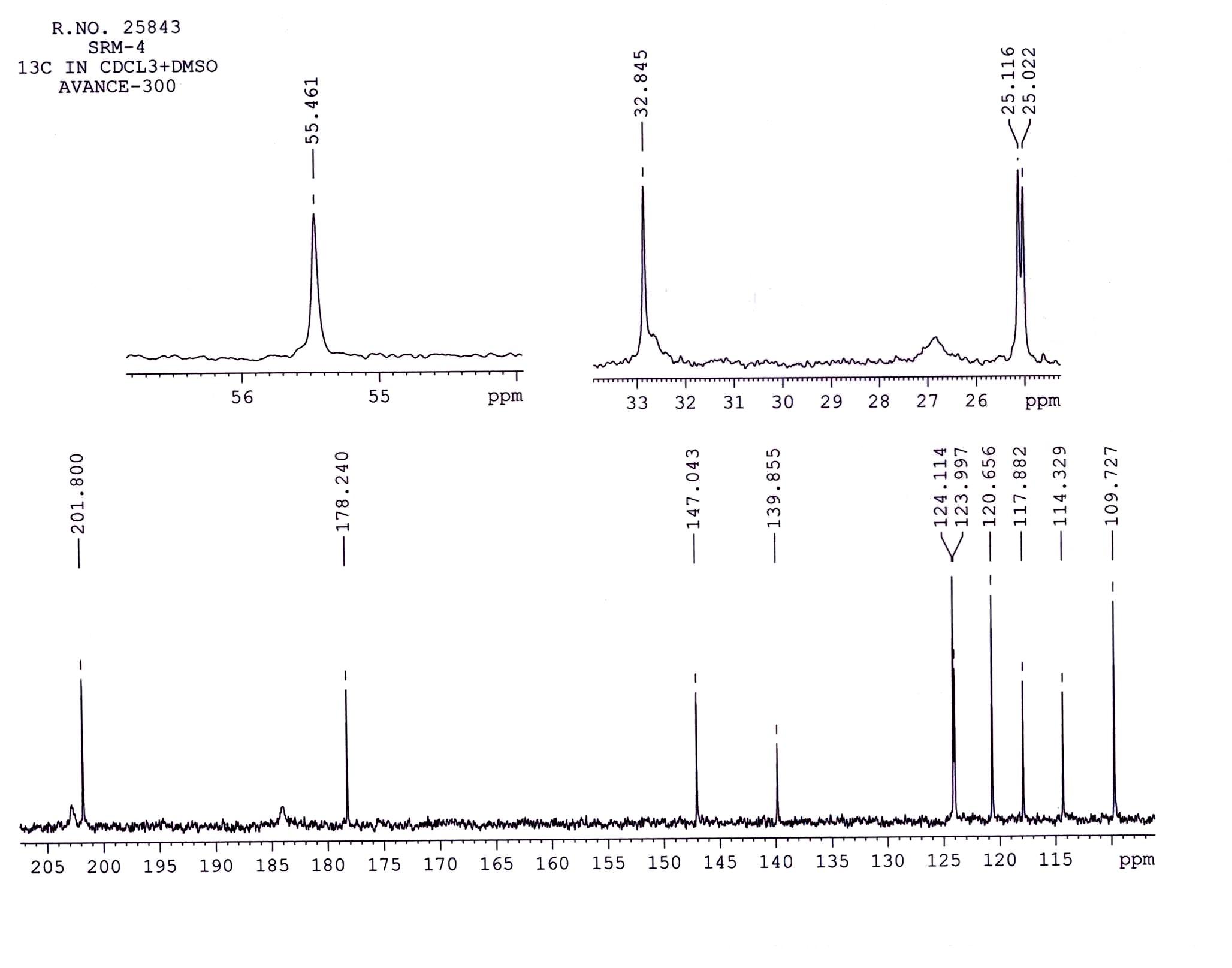 |
| Supplementary Figure S54 from Resolved ^13^C NMR spectrum of compound 3o |
| **-S55-** |
| 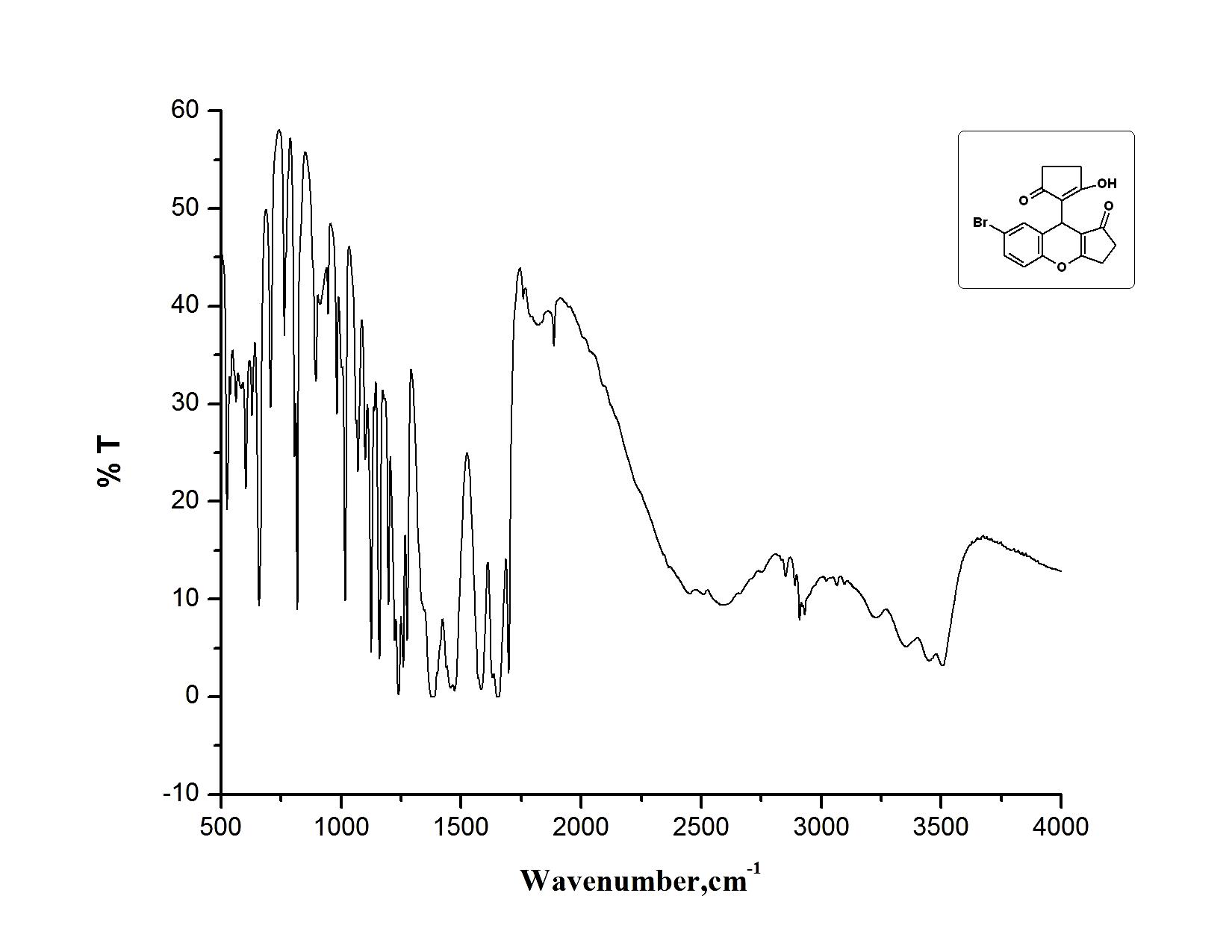 |
| Supplementary Figure S55 from FT-IR spectrum of compound 3p |
| **-S56-** |
| 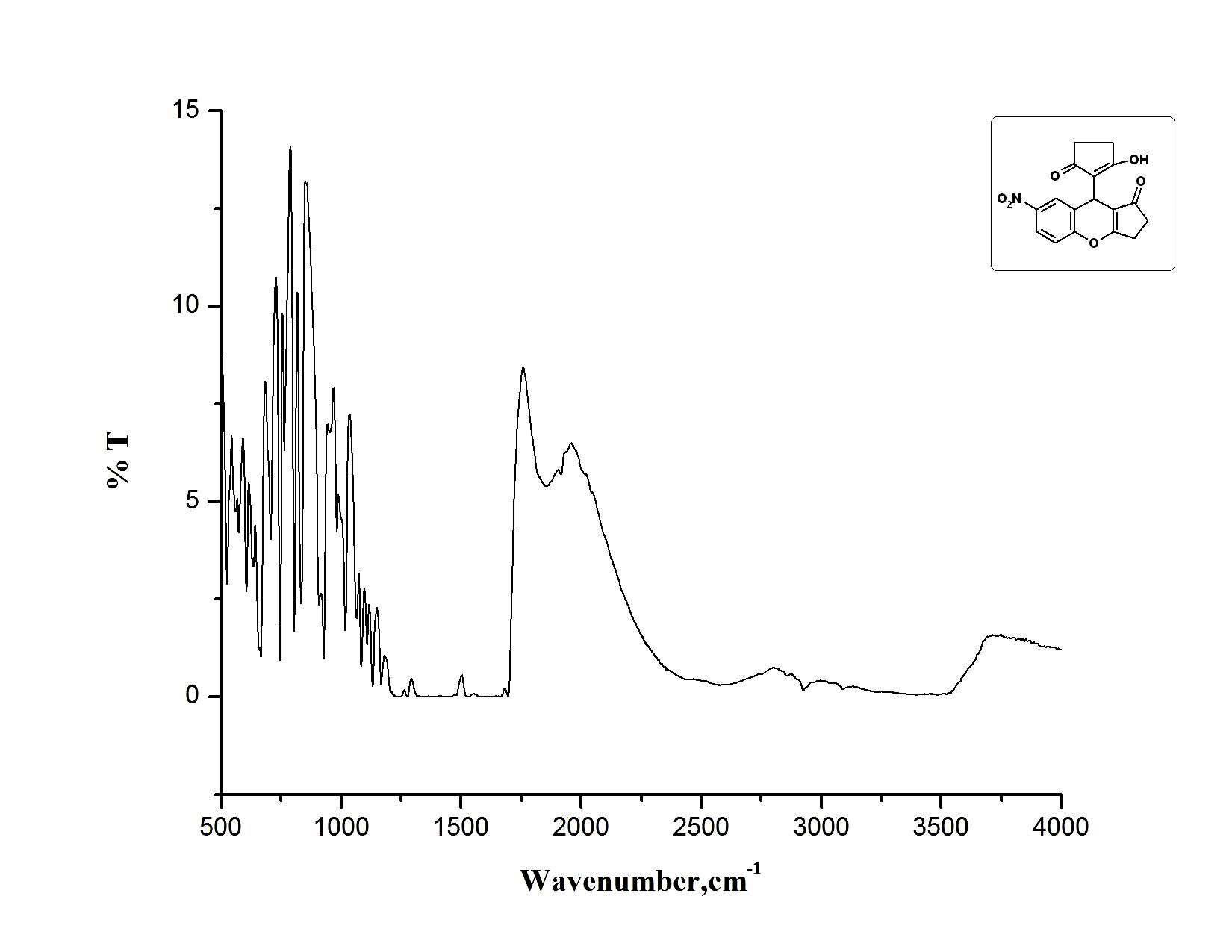 |
| Supplementary Figure S56 from FT-IR spectrum of compound 3q |
| **-S57-** |
| 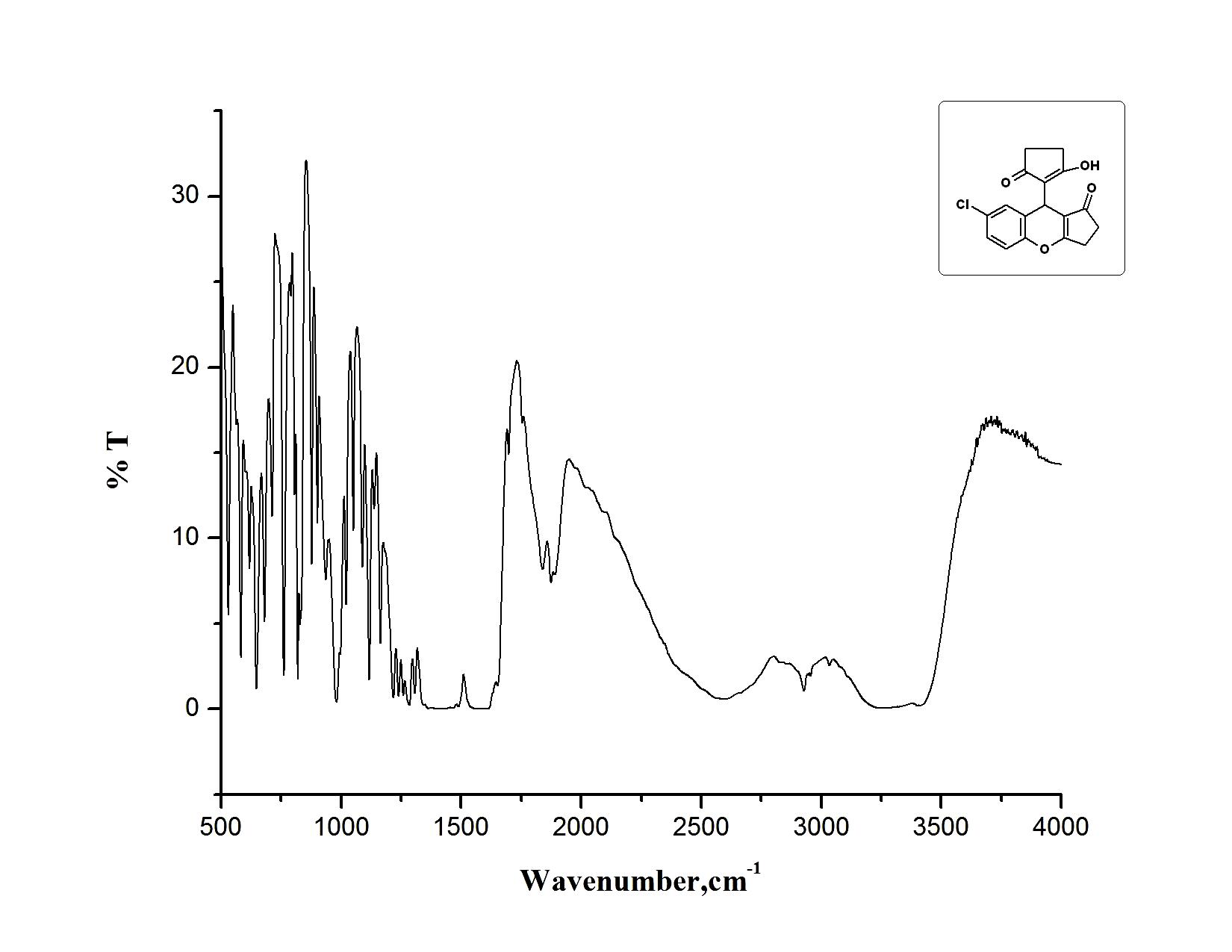 |
| Supplementary Figure S57 from FT-IR spectrum of compound 3r |
| **-S58-** |
| **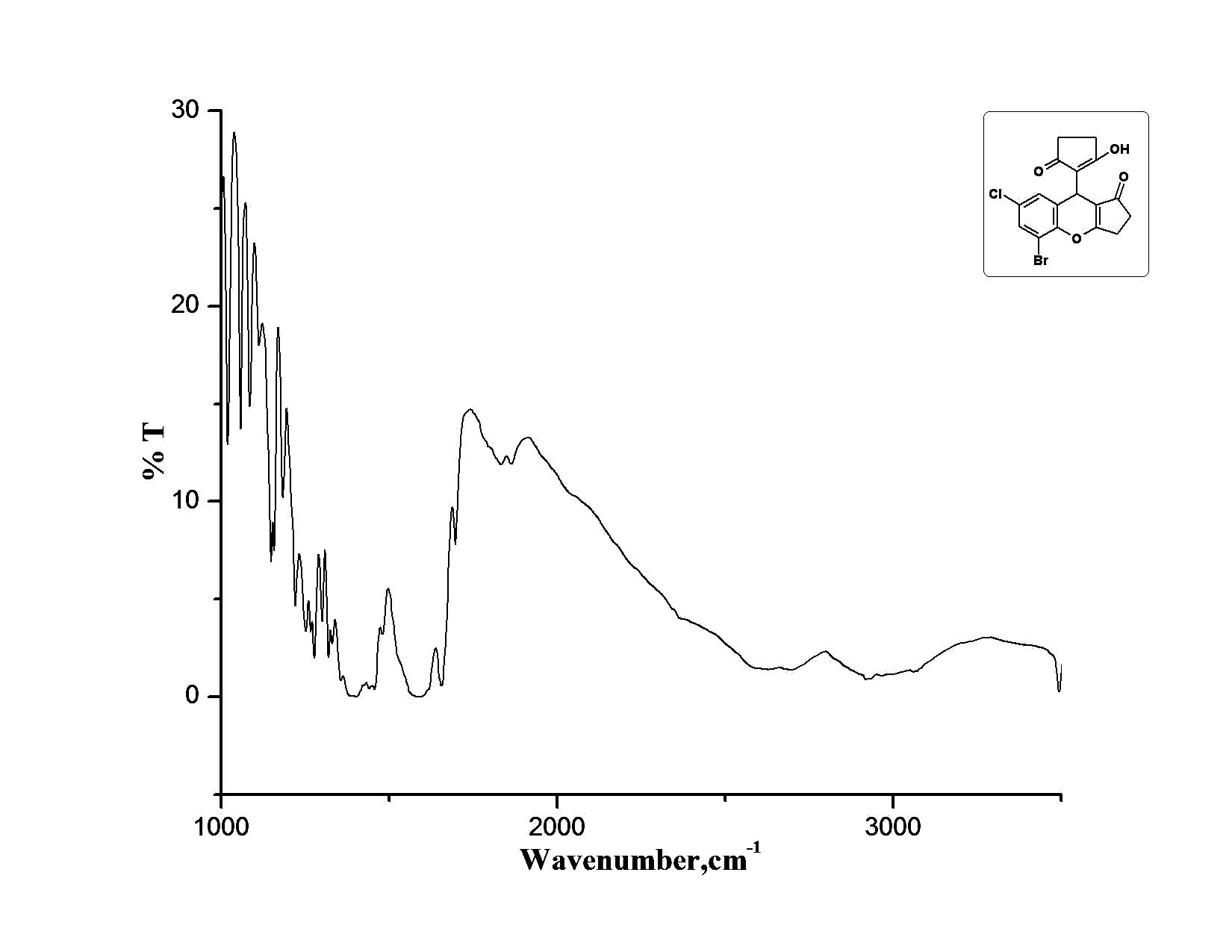** |
| Supplementary Figure S58 from FT-IR spectrum of compound 3s |
| **-S59-** |
| **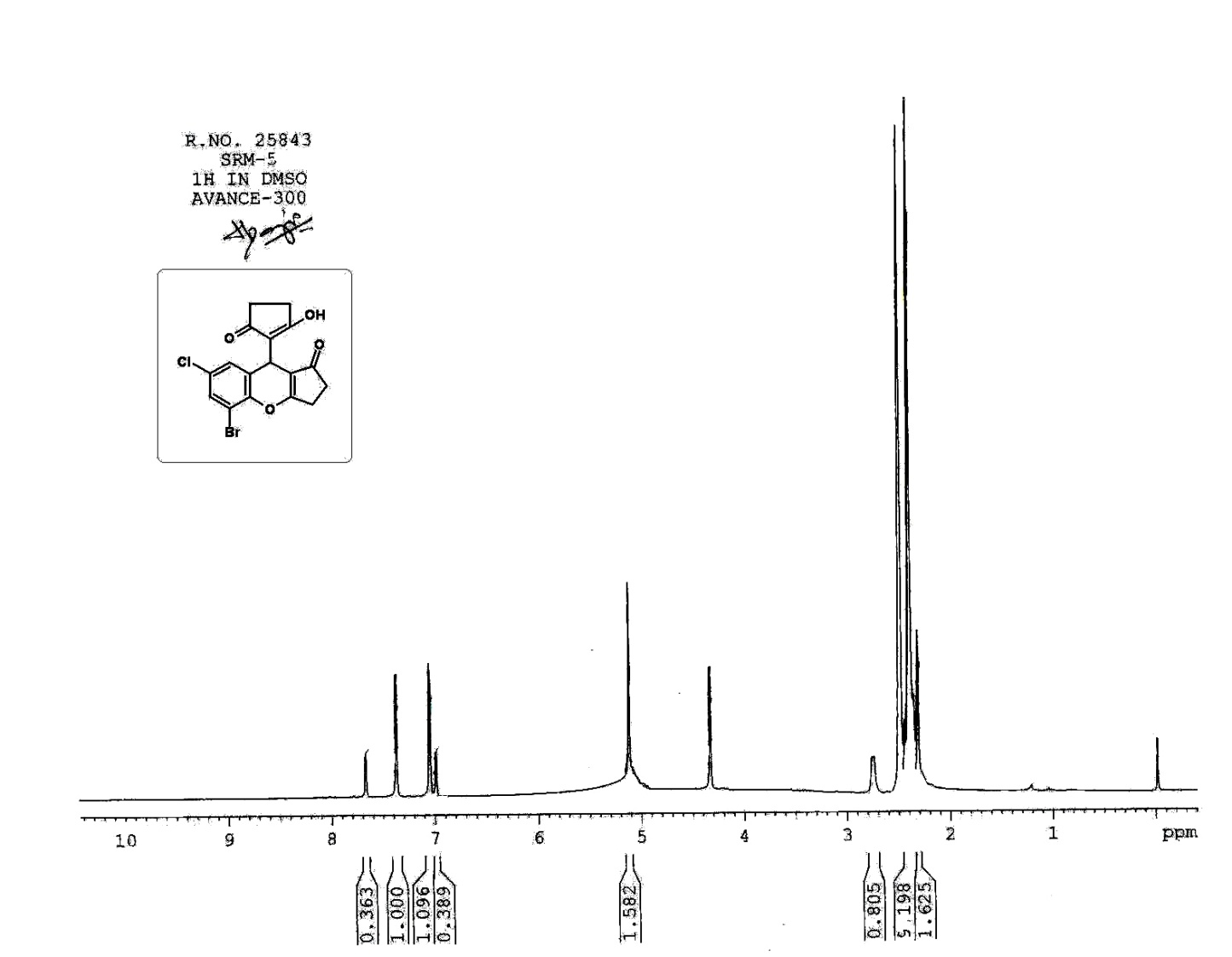** |
| Supplementary Figure S59 from ^1^HNMR spectrum of compound 3s |
| **-S60-** |
| **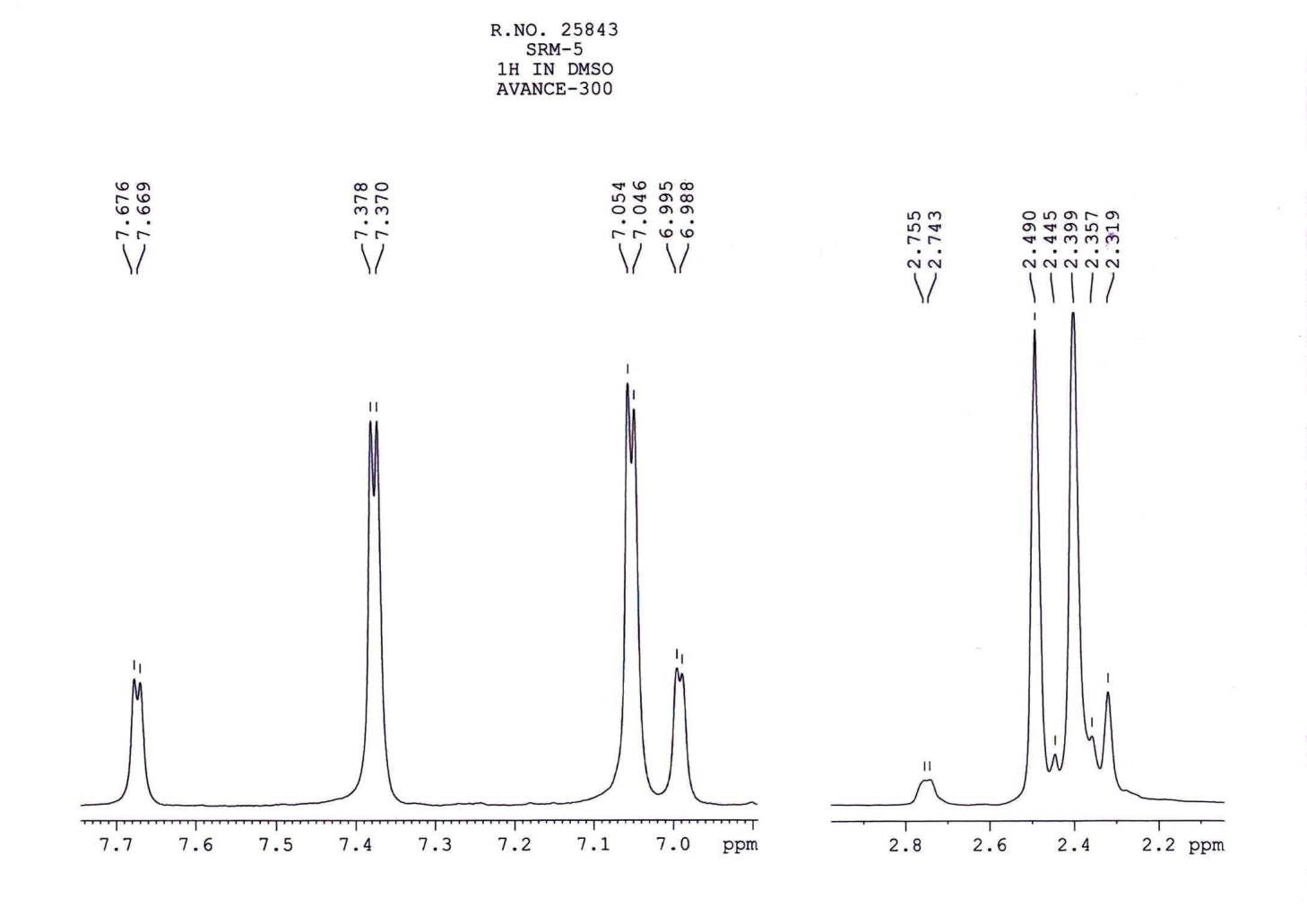** |
| Supplementary Figure S60 from Resolved^1^HNMR spectrum of compound of 3s |
| **-S61-** |
| **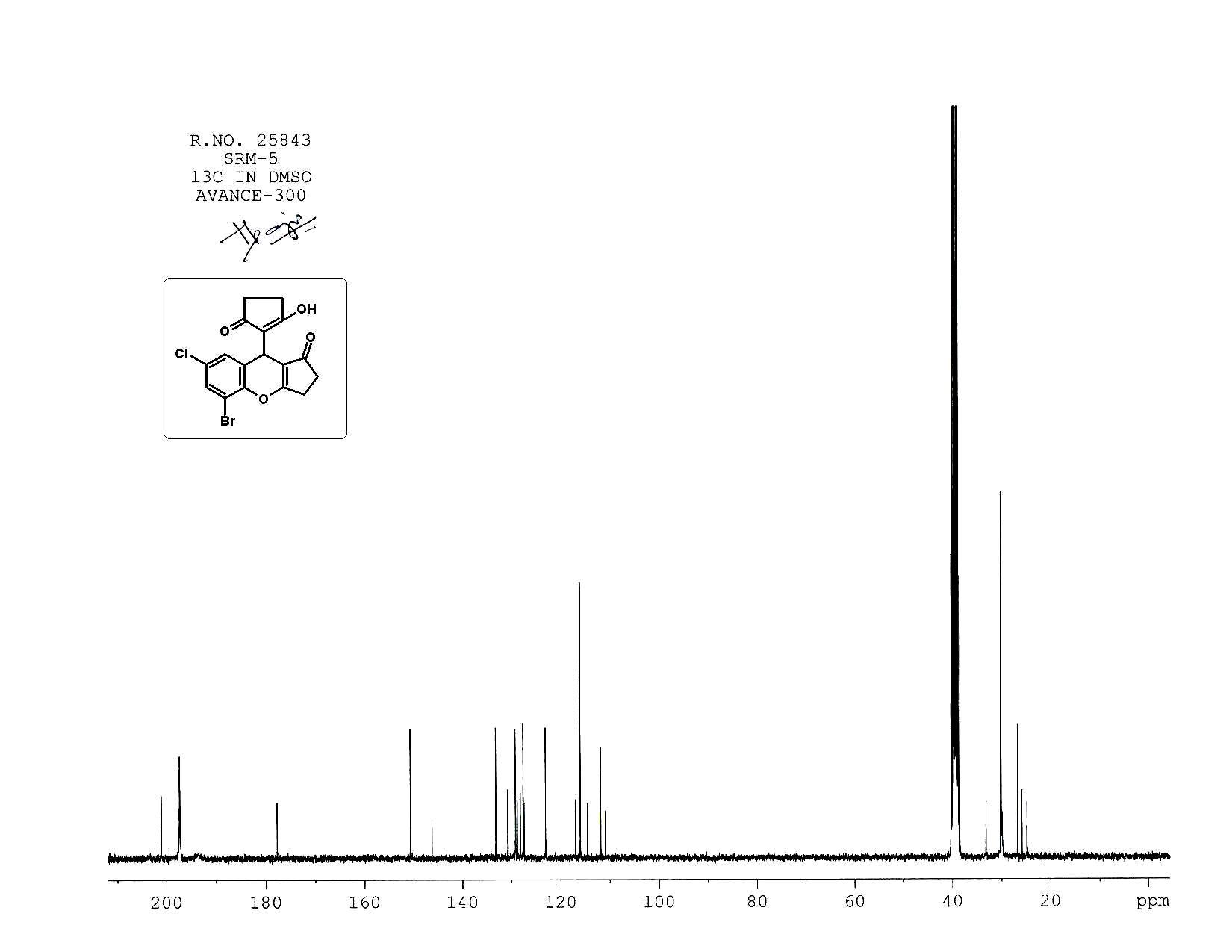** |
| Supplementary Figure S61 from ^13^CNMR spectrum of compound 3s |
| **-S62-** |
| **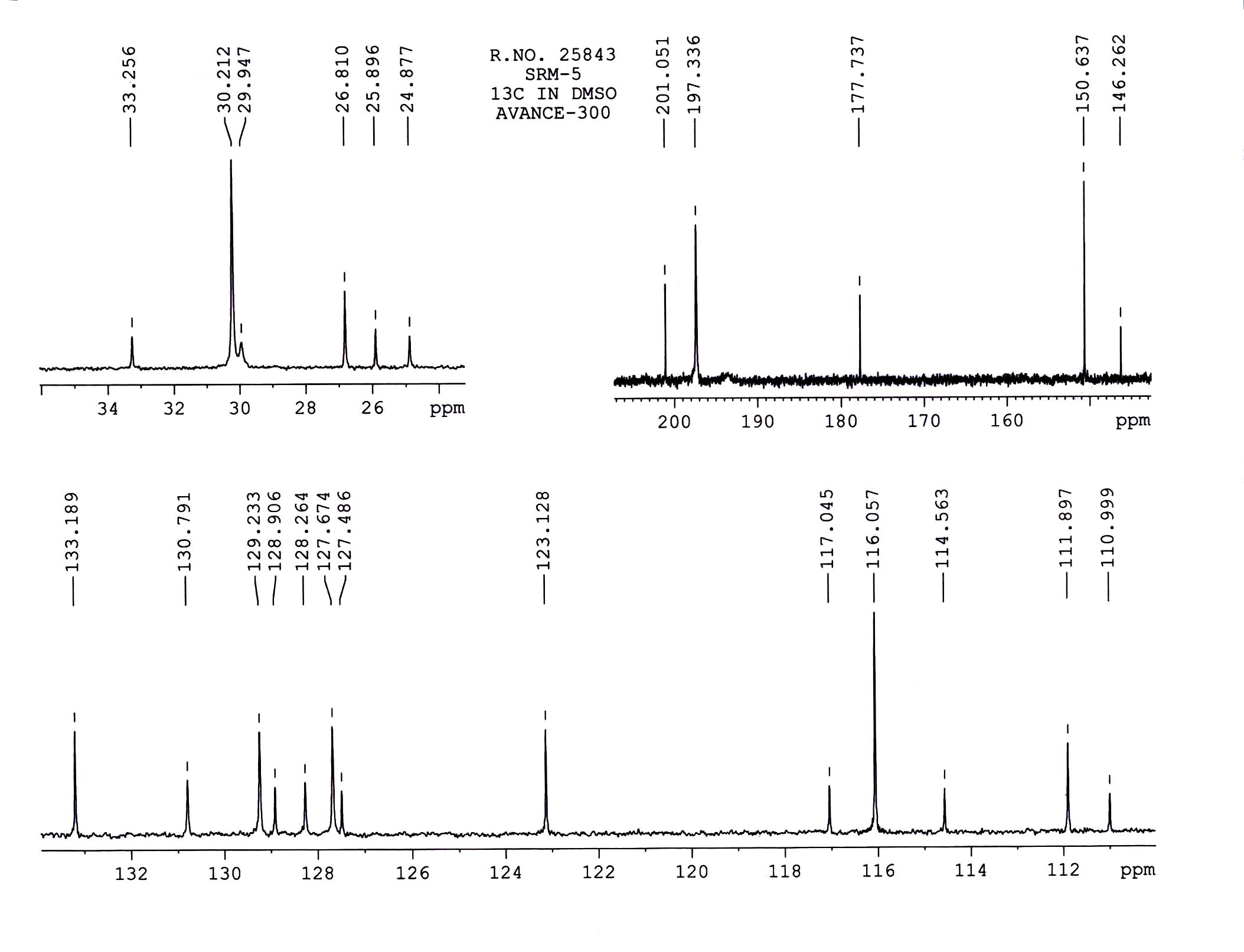** |
| Supplementary Figure S62 from Resolved ^13^C NMR spectrum of compound of 3s |
| **-S63-** |
| **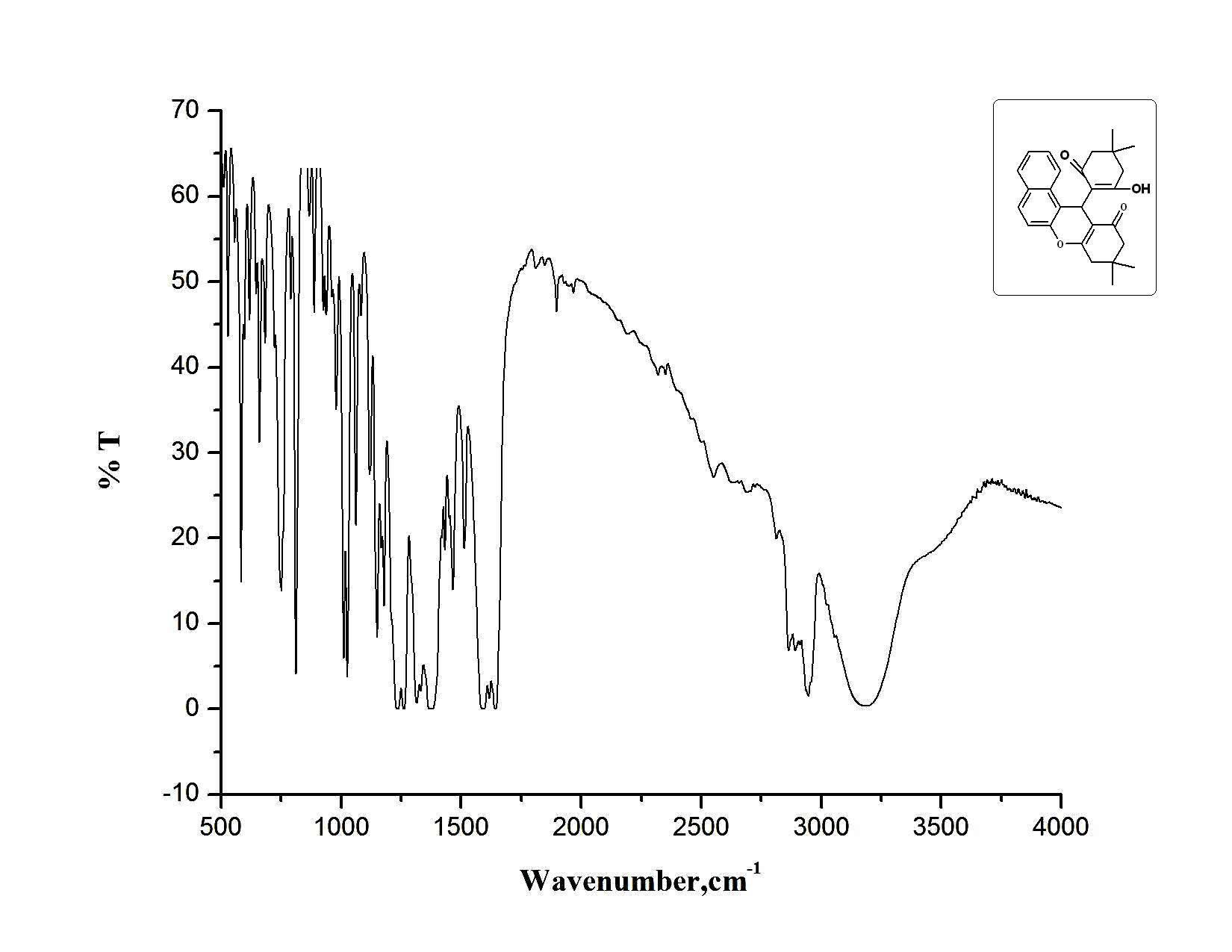** |
| Supplementary Figure S63 from FT-IR spectrum of 5a |
| **-S64-** |
| **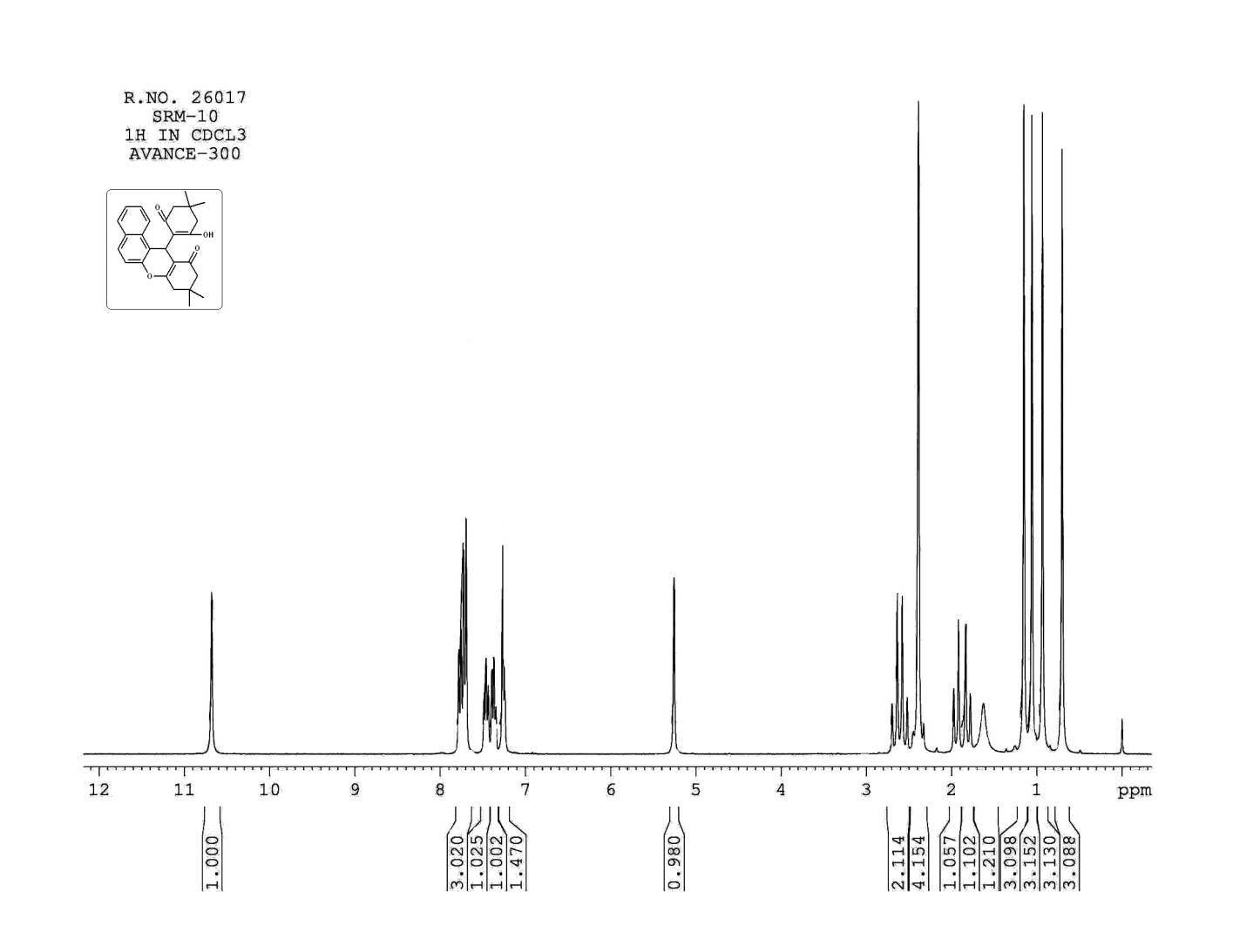** |
| Supplementary Figure S64 from ^1^HNMR spectrum of compound 5a |
| **-S65-** |
| **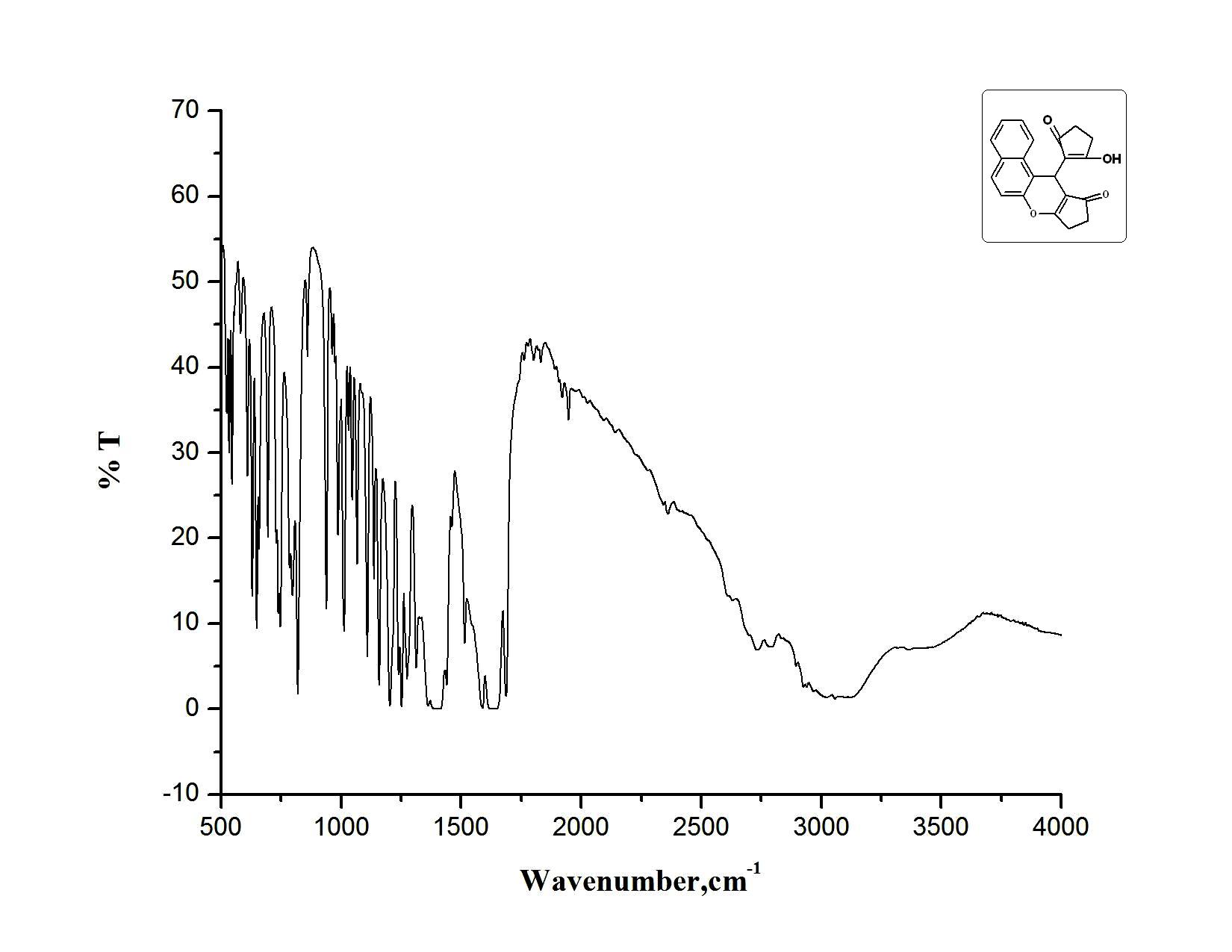** |
| Supplementary Figure S65 from FT-IR spectrum of 5c |
| **-S66-** |
| **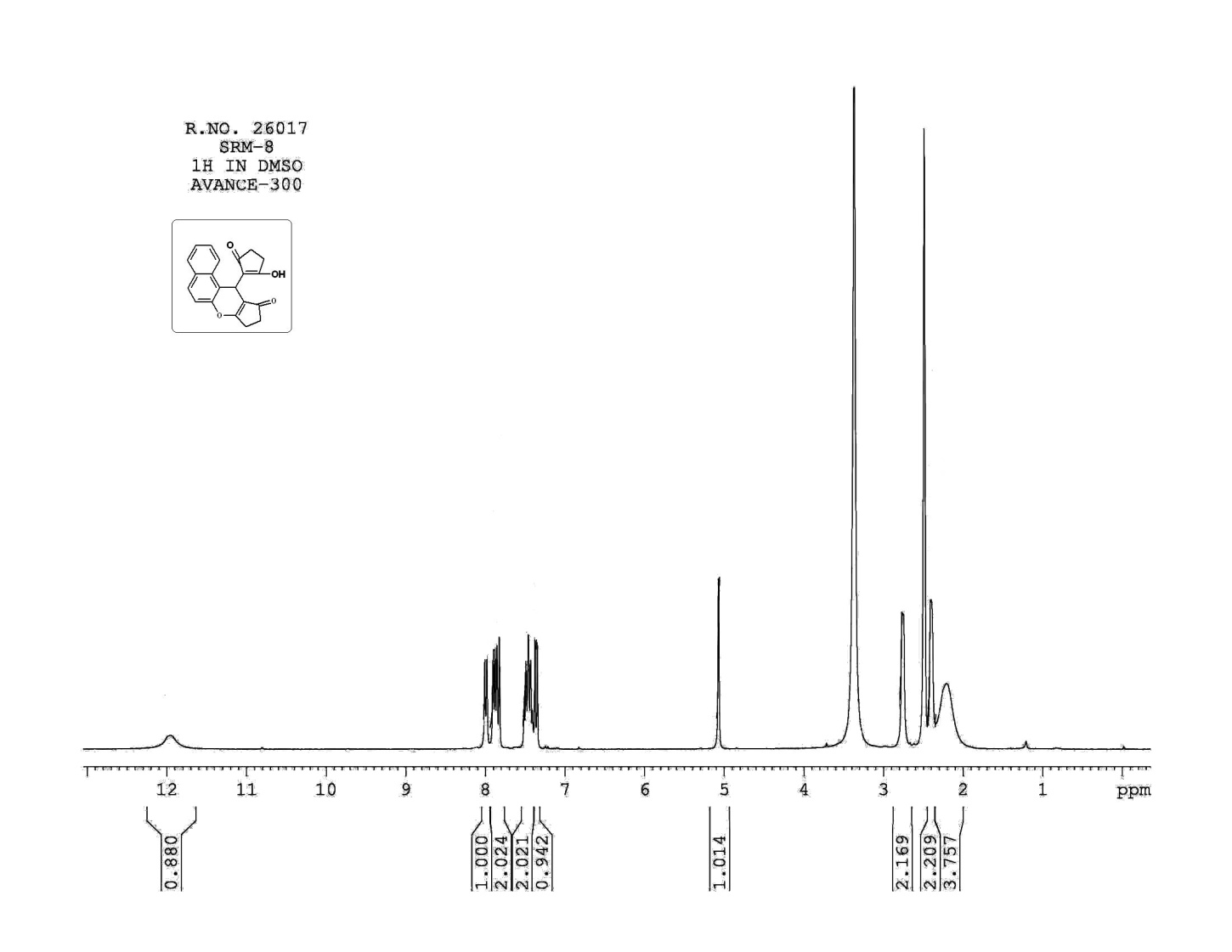** |
| Supplementary Figure S66 from ^1^H NMR spectrum of compound 5c |
|  |
|  |
|  |
| **-S67-** |
| ^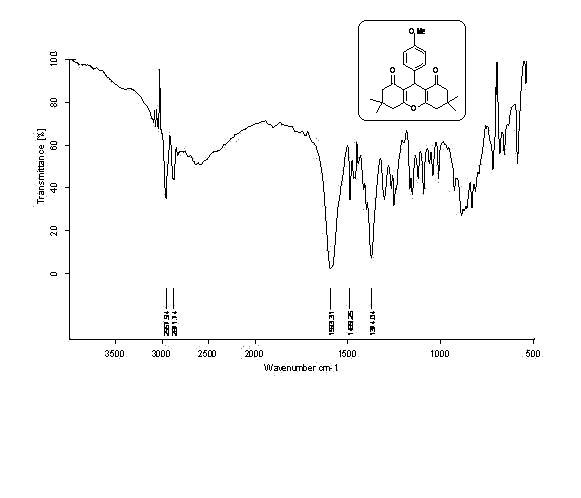^ |
| Supplementary Figure S67 from FT-IR spectrum of 6c |
| **-S68-** |
| ^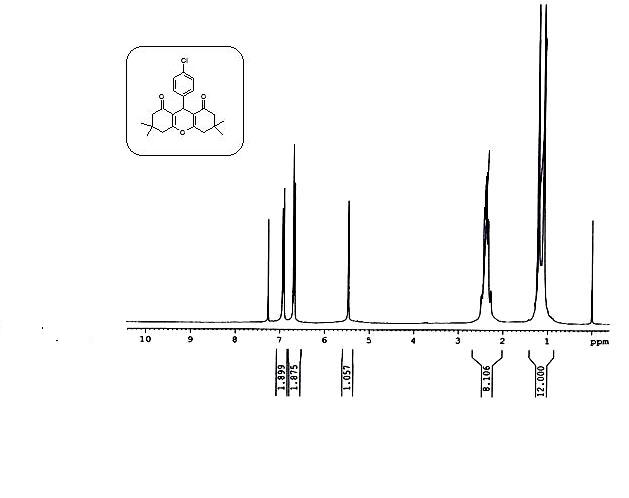^ |
| Supplementary Figure S68 from ^1^H NMR spectrum of compound 6d |
| **-S69-** |
| ^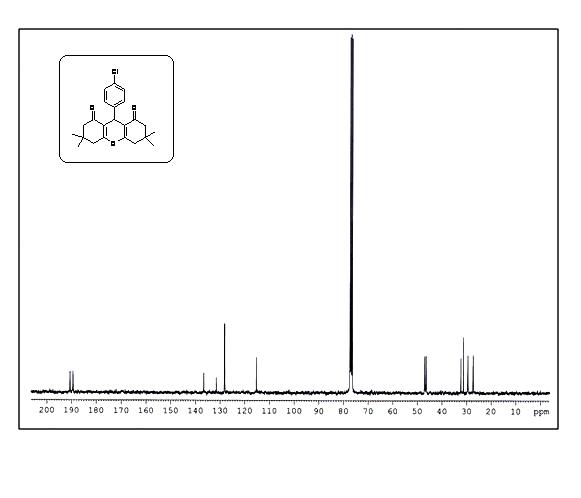^ |
| Supplementary Figure S69 from ^13^CNMR spectrum of compound 6d |
| **-S70-** |
| ^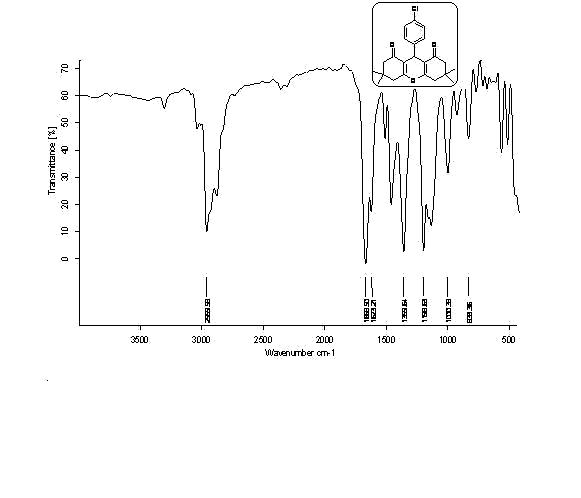^ |
| Supplementary Figure S70 from FT-IR spectrum of 6d |
| **-S71-** |
| ^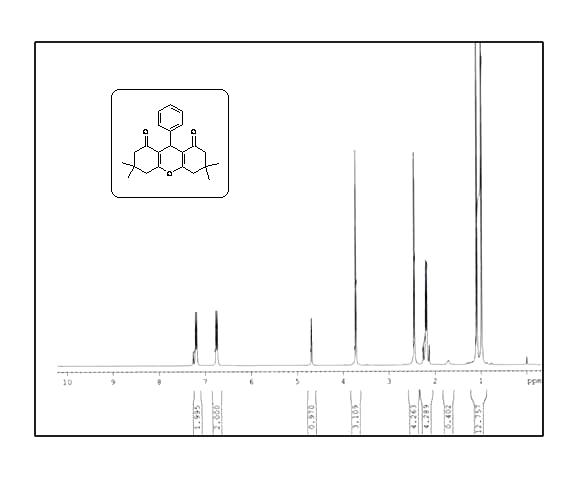^ |
| Supplementary Figure S71 from ^1^H NMR spectrum of compound 6a |
| **-S72-** |
| ^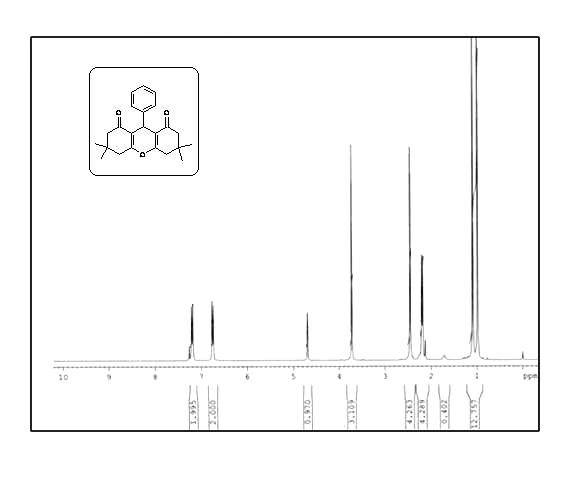^ |
| Supplementary Figure S72 from ^13^CNMR spectrum of compound 6a |
| **-S73-** |
| ^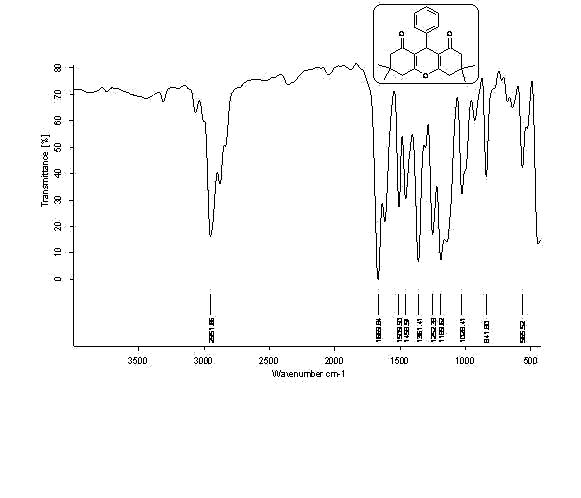^ |
| Supplementary Figure S73 from FT-IR spectrum of 6a |
| **-S74-** |
| ^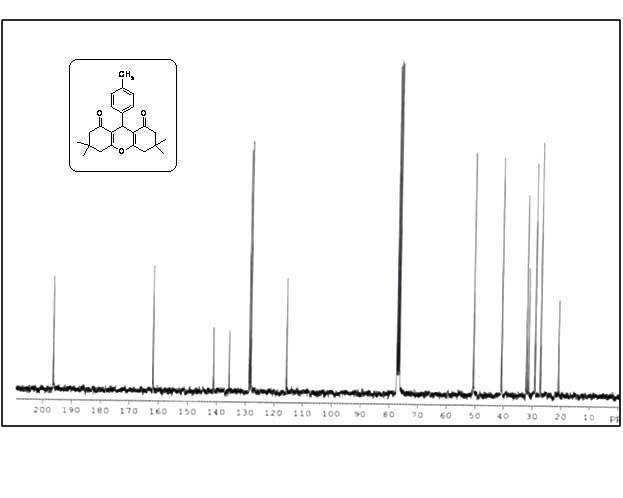^ |
| Supplementary Figure S74 from ^13^CNMR spectrum of compound 6k |
